# Photoresponsive Nanocarriers Based on Lithium Niobate Nanoparticles for Harmonic Imaging and On-Demand Release of Anticancer Chemotherapeutics

**DOI:** 10.1021/acsnanoscienceau.1c00044

**Published:** 2022-06-03

**Authors:** Adrian Gheata, Geoffrey Gaulier, Gabriel Campargue, Jérémy Vuilleumier, Simon Kaiser, Ivan Gautschi, Florian Riporto, Sandrine Beauquis, Davide Staedler, Dario Diviani, Luigi Bonacina, Sandrine Gerber-Lemaire

**Affiliations:** † Institute of Chemical Sciences and Engineering, Ecole Polytechnique Fédérale de Lausanne, Group for Functionalized Biomaterials, EPFL SB ISIC SCI-SB-SG, Station 6, Lausanne CH-1015, Switzerland; ‡ Department of Applied Physics, Université de Genève, 22 Chemin de Pinchat, Genève CH-1211, Switzerland; § Department of Biomedical Sciences, Université de Lausanne, 7 Rue du Bugnon, Lausanne CH-1005, Switzerland; ∥ SYMME, Université Savoie Mont-Blanc, Annecy F-74000, France

**Keywords:** controlled drug release, erlotinib derivative, EGFR-positive cells, harmonic nanoparticles, light-triggered uncaging, second harmonic generation, surface functionalization

## Abstract

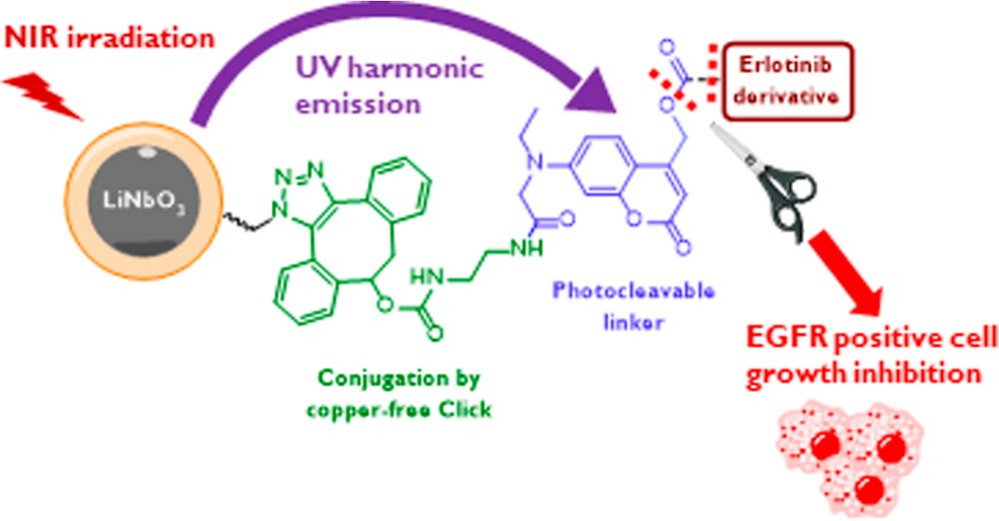

Nanoparticle-based drug delivery systems have the potential for increasing the efficiency of chemotherapeutics by enhancing the drug accumulation at specific target sites, thereby reducing adverse side effects and mitigating patient acquired resistance. In particular, photo-responsive nanomaterials have attracted much interest due to their ability to release molecular cargos on demand upon light irradiation. In some settings, they can also provide complementary information by optical imaging on the (sub)cellular scale. We herein present a system based on lithium niobate harmonic nanoparticles (LNO HNPs) for the decoupled multi-harmonic cell imaging and near-infrared light-triggered delivery of an erlotinib derivative (**ELA**) for the treatment of epidermal growth factor receptor (EGFR)-overexpressing carcinomas. The **ELA** cargo was covalently conjugated to the surface of silica-coated LNO HNPs through a coumarinyl photo-cleavable linker, achieving a surface loading of the active molecule of 27 nmol/mg NPs. The resulting nanoconjugates (**LNO-CM-ELA** NPs) were successfully imaged upon pulsed laser excitation at 1250 nm in EGFR-overexpressing human prostate cancer cells DU145 by detecting the second harmonic emission at 625 nm, in the tissue transparency window. Tuning the laser at 790 nm resulted in the uncaging of the **ELA** cargo as a result of the second harmonic emission of the inorganic HNP core at 395 nm. This protocol induced a significant growth inhibition in DU145 cells, which was only observed upon specific irradiation at 790 nm, highlighting the promising capabilities of **LNO-CM-ELA** NPs for theranostic applications.

Mutations in the epidermal growth factor receptor (EGFR) gene leading to the overexpression of the transmembrane protein have been associated with a variety of human carcinomas,^1^ including non-small-cell lung cancer^2^ (NSCLC, more than 60% of the cases), glioblastomas^3^ (50%), and epithelial cancers^4^ (80–100%). This led to the identification of the EGFR gene as a proto-oncogene and relevant therapeutic target for these cancer types.^5^ Amplification of the EGFR protein causes its uncontrolled activation and is correlated with tumor cell proliferation, metastasis, and angiogenesis.^6^ Tyrosine kinase (TK) inhibitors emerged as potential chemotherapeutics for the treatment of EGFR-mutated malignancies. Their mode of action consists in blocking the adenosine triphosphate (ATP) binding site of the EGFR, resulting in the inhibition of its auto-phosphorylation and downstream signaling mechanism, thereby limiting cell growth.^7^ Notably, erlotinib (Tarceva) was developed as a small-molecule TK inhibitor and approved for the treatment of NSCLC and pancreatic cancer.^8^ Despite its potency and selectivity as a TK inhibitor, erlotinib exhibits a low bioavailability, which requires the daily administration of a significant dose (150 mg for NSCLC treatment), over a long period of time, to achieve therapeutic effects.^9,10^ Such treatment modality may lead to many potential adverse effects, commonly including rashes, diarrhea, and fatigue, and in rarer cases, gastrointestinal tract perforations, Stevens–Johnson syndrome, or pulmonary damages, which can even be fatal.^11,12^ Another important limitation to erlotinib treatments, which is common to most small-molecule TK inhibitors, is patient acquired resistance.^13^ The latter most frequently results from mutation of the EGFR ATP binding pocket and typically affects patients after 9 to 15 months of treatment.^14^

The development of nanoparticle-based formulations for this class of chemotherapeutics emerged as an appealing strategy to increase their efficiency at lower dosages due to enhanced drug accumulation at the target site. Various nanotechnology-oriented delivery systems were recently disclosed to increase the therapeutic efficiency of erlotinib by limiting dose-related side effects and potentially delaying or even eliminating acquired resistance.^15,16^ Most of the nanodevice-based strategies reported involve encapsulation in polymer-based nanocarriers,^17,18^ liposomes,^19^ and lipid–polymer hybrid nanoparticles.^20^

A different strategy which, to the best of our knowledge, has not yet been extensively investigated in the case of similar TK inhibitors is drug delivery through systems, which combine optically active inorganic nanomaterials [quantum dots, plasmonic nanoparticles, and upconversion nanoparticles (UCNPs)]^21−23^ with photo-cleavable scaffolds (*ortho*-nitrobenzyl, *para*-hydroxyphenacyl, and coumarinyl derivatives).^24−26^ This approach offers precise control over the release, both in time and space,^27^ while adding complementary bioimaging capabilities for theranostic applications.^28^ Most photo-responsive caging moieties rely on high-energy UV light excitation, which severely limits their application in biomedical setups due to phototoxicity and poor tissue penetration depth.^29^ Nanocarriers based on lanthanide-doped UCNPs can overcome these shortcomings due to their ability to convert two or more low-energy near-infrared (NIR) excitation photons into UV or visible emission. The favorable properties of NIR light, including comparatively large penetration and decreased photodamage in living cells and tissues, has prompted the development of UCNP-based delivery systems for the in vitro and in vivo targeted release of biomolecules,^30^ small interfering RNA,^31^ and anticancer chemotherapeutics.^32^

Harmonic nanoparticles (HNPs)^33^ share with UCNPs the ability to convert excitation photons to shorter wavelengths; however, the underlying photo-physical process is substantially different. In UCNPs, the emission follows a series of sequential absorption events proceeding through the real energy levels of lanthanide ions, while harmonic generation in HNPs is an inherently nonlinear process occurring through virtual states, which exist exclusively during the interaction of the particle with an intense electromagnetic field. The approach therefore relies on ultrafast (femtosecond) laser excitation to reach large peak intensities at comparatively low pulse energies. This stringent technical requirement definitively makes HNPs a more niche approach than UCNPs. On the other hand, HNPs provide some complementary advantages that are worth investigating. While UCNP emission is typically associated with long lifetimes,^34^ the process of harmonic generation is practically instantaneous and therefore compatible with scanning (three-dimensional) approaches. The distinctive features of HNPs include their coherent emission,^35^ absence of bleaching/blinking when excited within the material transparency range, and wide spectral flexibility.^36−38^ The nonlinear generation efficiency is material- and wavelength-dependent; the orientation-averaged second-order susceptibility of lithium niobate (LNO) nanocrystals under 700–1300 nm excitation was reported by Riporto et al.^39^ The possibility to colocalize the simultaneously emitted second and third harmonic signals upon excitation in the shortwave infrared^40^ windows make HNPs a promising approach for selected imaging applications, such as cell monitoring in tissues.^41−44^ The tunable response of HNPs offers an opportunity for decoupled imaging modality and photo-triggered uncaging of surface-conjugated molecular cargos. We previously demonstrated that the harmonic emission of bismuth ferrite (BiFeO_3_, BFO) or LNO HNPs can be used as a local trigger for the controlled release of caged l-tryptophan^45^ and anticancer drug chlorambucil.^46^

Herein, we disclosed the controlled release of an analogue of the EGFR TK inhibitor erlotinib (**ELA**) from the surface of LNO-based nanoconjugates. Making use of a coumarinyl (CM) tether, the **ELA** cargo was covalently immobilized on silica-coated LNO HNPs through copper-free azide for strained alkyne [3 + 2] cycloaddition. The **ELA**-functionalized nanocarriers were first assessed for their nonlinear optical imaging properties upon incubation with the EGFR-overexpressing DU145 human androgen-dependent prostate cancer cells. Then, we evaluated their cytotoxic effect in vitro by tuning the NIR irradiation to 790 nm, which induced the release of **ELA** and led to a marked decrease in cancer cell viability. These decoupled imaging and NIR light-triggered delivery protocols demonstrated the capability of harmonic nanoconjugates to act as efficient exogenous optical probes and photoresponsive drug delivery systems.

## Results and Discussion

### Synthesis of the Caged Erlotinib Derivative for Conjugation to LNO HNPs

**Scheme 1:**
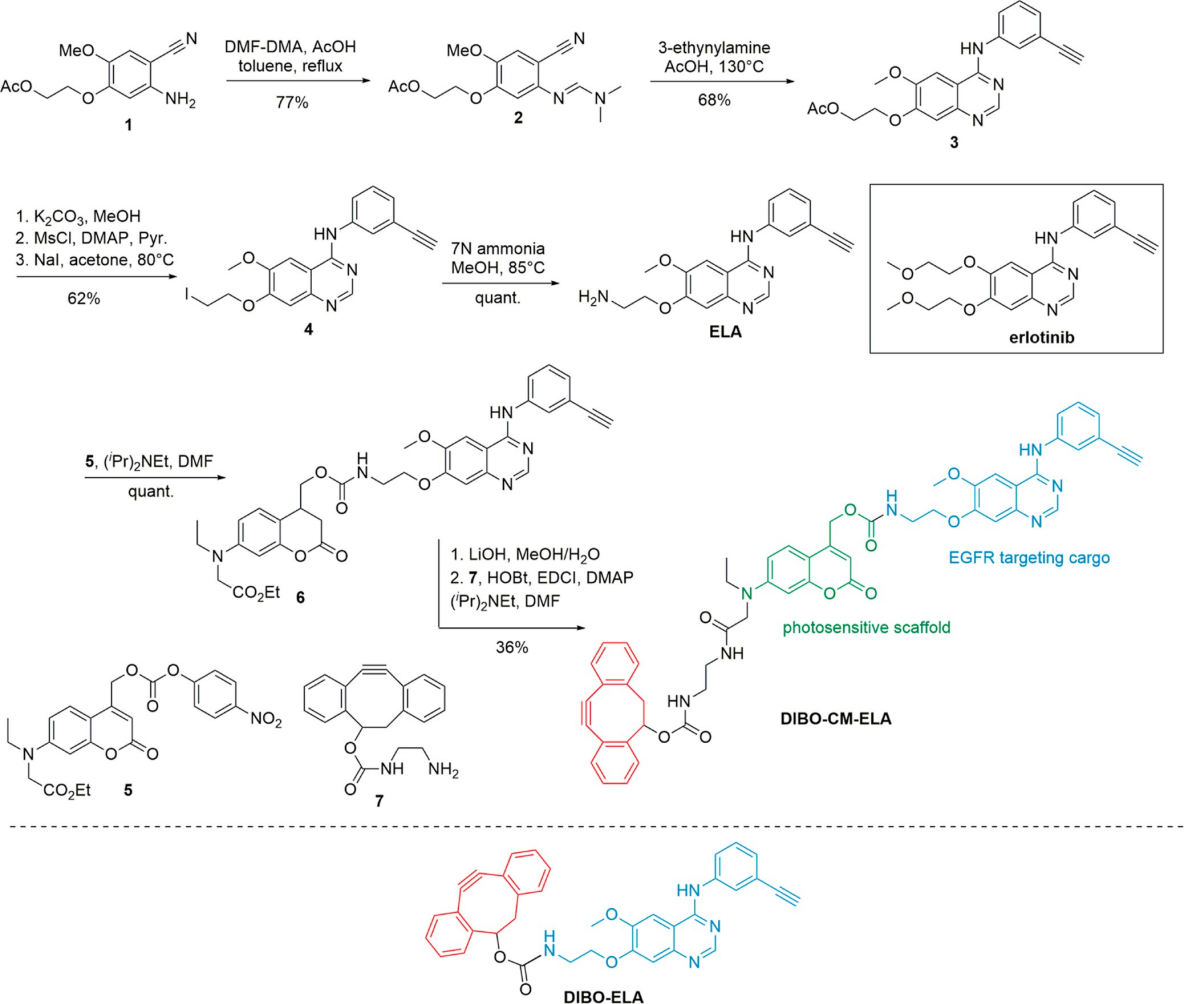
Synthesis of the Caged Erlotinib Derivative

The chemical structure of erlotinib features a 4-aminoquinazoline core and an alkyne-substituted aromatic ring, which interact with the tyrosine residue of the EGFR and the ATP receptor, respectively (Scheme 1). The design of an erlotinib analogue, amenable to covalent conjugation to a photosensitive caging group while maintaining its ability to interact with the EGFR ATP binding site, was based on the modification of the C6 and C7 aromatic substituents which are directed outside of the binding pocket.^47^ Starting from the aniline derivative **1** (synthesis protocols detailed in Supporting Information, S3), condensation with dimethylformamide dimethylacetal (DMF-DMA) followed by Dimroth rearrangement in the presence of 3-ethynylaniline afforded the substituted 4-anilinoquinazoline scaffold (**3**)^48^ in 54% yield (two steps). Methanolysis of the acetate followed by conversion of the resulting primary alcohol into a leaving group allowed for the introduction of a terminal primary amine by nucleophilic substitution, affording the erlotinib derivative **ELA**. Introduction of the photosensitive spacer proceeded through conjugation to the activated 7-amino-coumarine derivative **5** (synthesis protocols detailed in Supporting Information, S9), in quantitative yield. Subsequent saponification of the ethyl ester and coupling with the dibenzocyclooctyne-containing unit **7** (synthesis protocols detailed in Supporting Information, S15) afforded the targeted caged erlotinib derivative **CM-ELA**, ready to undergo strain-promoted [3 + 2] cycloaddition reaction. For control experiments, **ELA** was also coupled to a precursor of compound **7** to provide **DIBO-ELA** (synthesis protocols detailed in Supporting Information, S28), the equivalent of the drug delivery system **DIBO-CM-ELA** without the photosensitive coumarinyl linker.

### Preparation of LNO-Based Nanoconjugates

**Scheme 2:**
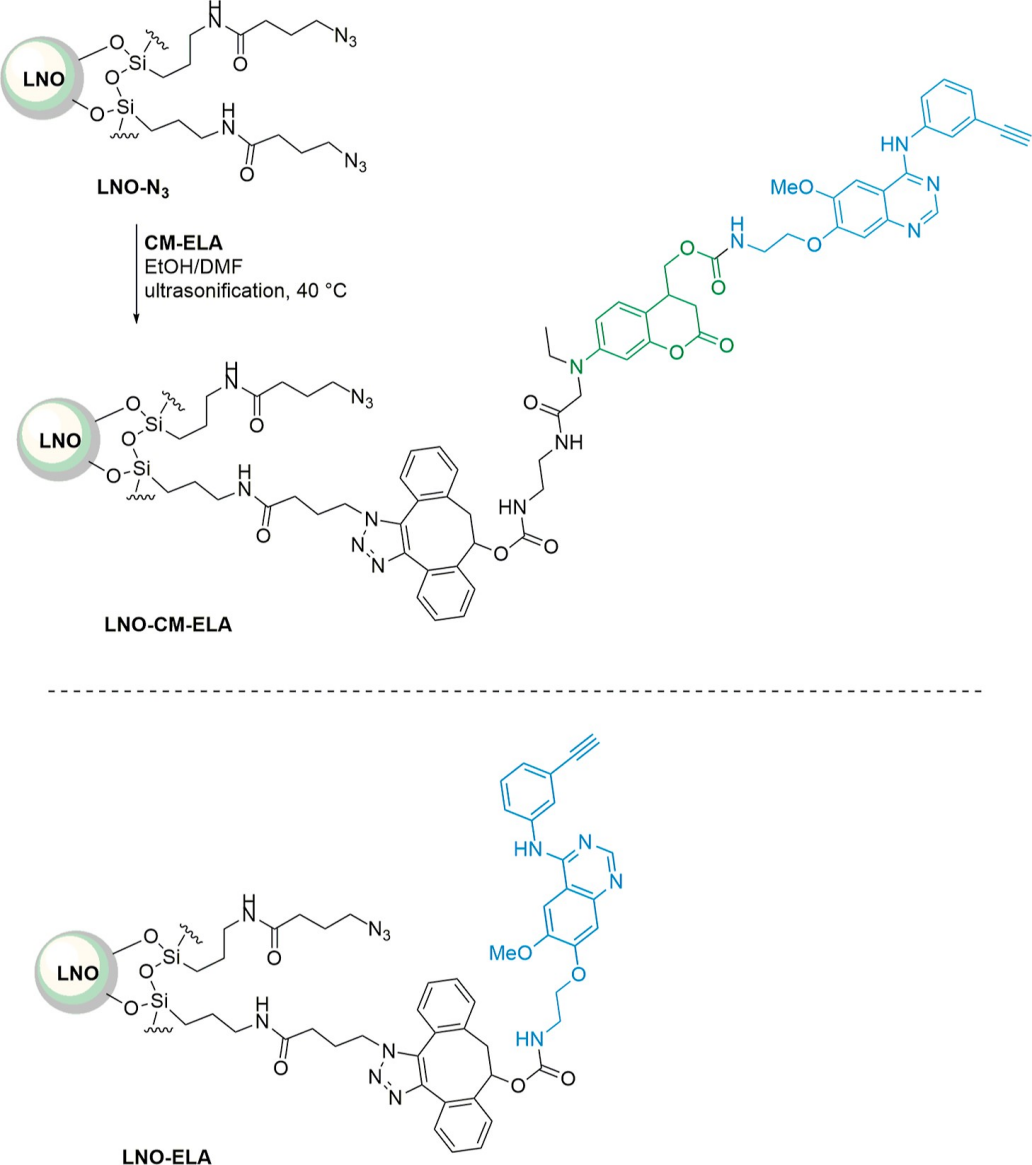
Surface Conjugation of the Caged Erlotinib Derivative to LNO HNPs

Surface silanization of LNO HNPs [prepared at a low hydrolysis rate of alkoxide precursors with a solvothermal route already described;^49^ for transmission electron microscopy (TEM) and X-ray diffraction (XRD) characterization of LNO HNPs, see Supporting Information S31] in the presence of 4-azido-*N*-(3-triethyloxysilylpropyl)butanamide provided **LNO-N**_**3**_ NPs (for the coating protocol and TEM characterization, see Supporting Information S31). The resulting suspension was subjected to the strain-promoted click reaction in in the presence of **CM-ELA**, under ultrasonication, to provide the functionalized **LNO-CM-ELA** NPs, which were stored in EtOH (2 mg/mL) (Scheme 2).

**Figure 1:**
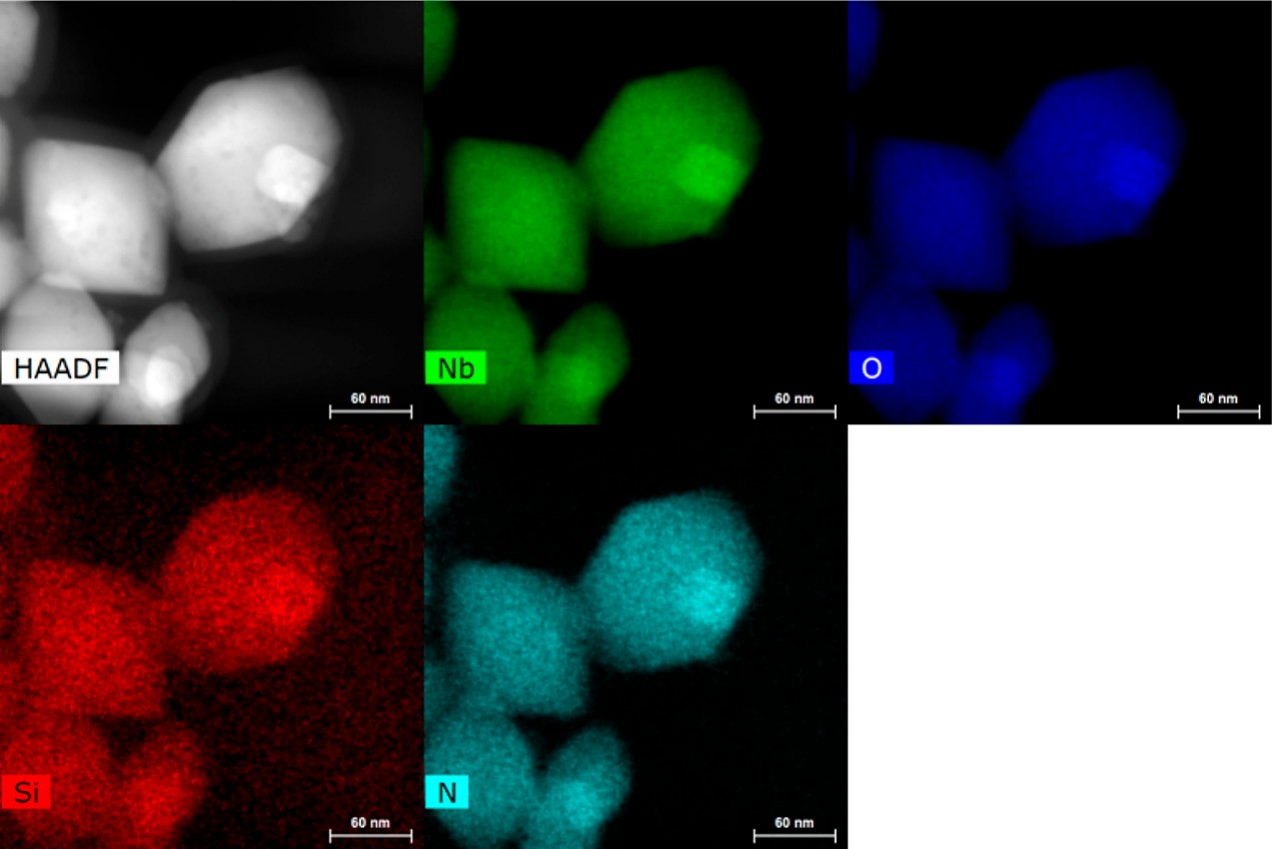
Representative STEM images of **LNO-N**_**3**_ NPs. A high-angle annular dark-field image, Nb EDX map, O EDX map, Si EDX map, and N EDX map; scale bar: 60 nm.

The surface loading of the erlotinib derivative **ELA** was estimated to be 27 nmol/mg NPs (for the quantification protocol, see Supporting Information S33, Figure S3). The coated and conjugated NPs were characterized by means of their mean hydrodynamic diameter (*d*), which increased upon surface silanization (from 77.8 ± 10.3 nm for bare LNO NPs to 146.3 ± 42.9 nm for **LNO-N**_**3**_ NPs) and post-functionalization (179.2 ± 4.4 nm for **LNO-CM-ELA** NPs), and surface charge (zeta potential, ZP), measured by dynamic light scattering (DLS) in phosphate-buffered saline (PBS) buffer (pH 7.4) at 22 °C (Table 1). The polydispersity index (PDI) of the final nanoconjugates (0.09) indicated their monodisperse size distribution and the absence of aggregate formation. These observations are consistent with the TEM analysis performed on the **LNO-N**_**3**_ (Figure 1, for images of a larger ensemble of NPs, see Supporting Information Figure S2A). The energy-dispersive X-ray (EDX) elemental analysis of **LNO-N**_**3**_ NPs revealed an atomic ratio of silicon to niobium of 7.8% (see Supporting Information, S32, Figure S2B).

**Table 1:** Hydrodynamic Diameter (*d*) and Surface Charge (ZP) Characteristics of **LNO** Derivatives Measured by DLS at 22 °C

	*d*^a^ (nm)	*d* standard deviation (nm)	ZP^a^ (mV)	ZP standard deviation (mV)	PDI
**LNO** NPs	77.8	10.3	–47.1	0.9	0.232
**LNO-N**_**3**_ NPs	146.3	42.9	–42.8	2.6	0.208
**LNO-CM-ELA** NPs	179.2	4.4	–40.8	1.4	0.09
**LNO-ELA** NPs	176.9	2.7	–38.1	2.6	0.115
**LNO-CM-ELA**, 72 h	191.2	26.9	–32.6	0.5	0.439
**LNO-CM-ELA**, 7d	161.1	5.8	–26.0	2.2	0.343
**LNO-CM-ELA**, cell medium^b^	219.1	7.1	not measurable	not measurable	0.215

a Mean hydrodynamic diameter (*d*, number distribution) and surface charge (ZP) were measured in PBS (pH 7.4) at 22 °C.

b Mean hydrodynamic diameter (*d*, number distribution) was measured in DMEM containing 10% FCS cell culture medium at 22 °C.

The stability of the nanoconjugate suspension was monitored by DLS upon storage of **LNO-CM-ELA** NPs in PBS for 24 h and 7 days at 4 °C (Table 1). No significant variation of the mean hydrodynamic diameter was observed. However, the increase in the PDI values indicated the formation of some aggregates in PBS over time. The size of **LNO-CM-ELA** NPs was also measured in cell culture medium used during the in vitro experiments.

For control experiments, **LNO-ELA** NPs (176.9 ± 2.7 nm) were prepared by conjugating **LNO-N**_**3**_ NPs with **DIBO-ELA** to model the photosensitive nanoconjugates **LNO-CM-ELA** NPs, without the coumarinyl spacer.

### Nonlinear Optical Imaging of **LNO-CM-ELA** NPs

**Figure 2:**
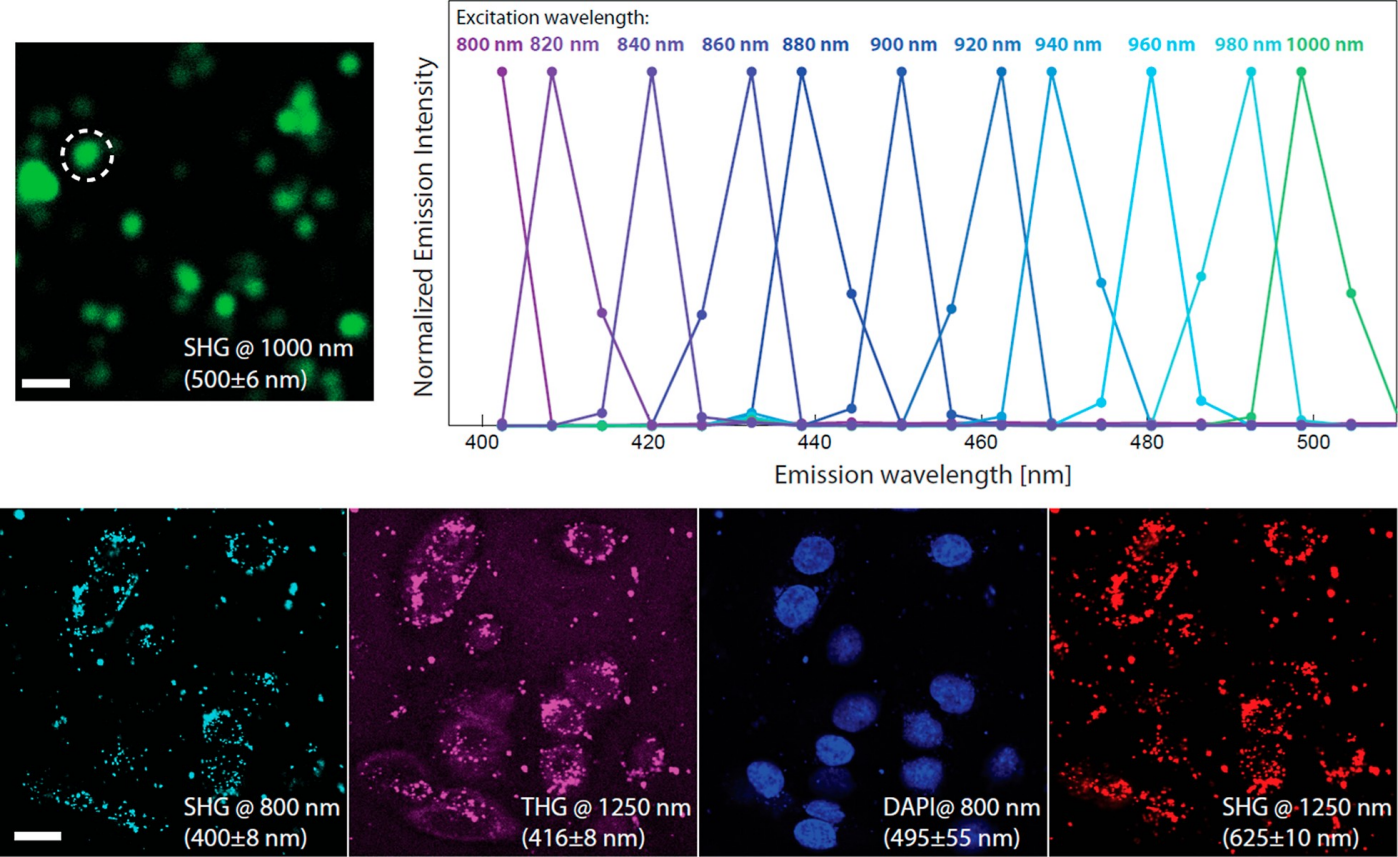
**Upper row**. **Left**. SHG image excited at 1000 nm of **LNO-CM-ELA** NPs deposited on a substrate. Scale bar 2 μm. **Right.** Normalized spectra of the SHG emissions upon excitation spanning from 800 to 1000 nm obtained by integrating the signal of the object indicated by the dashed outline. **Lower row**. Nonlinear microscopy images of DU145 cells (fixed) treated with **LNO-CM-ELA** NPs (50 μg/mL). The labels indicate the signal attribution (THG, SHG, and DAPI fluorescence), the excitation wavelength (@800, @1250 nm), and the acquisition spectral range. Scale bar: 20 μm.

In the upper row of Figure 2, we show the tunable spectral response of **LNO-CM-ELA** NPs deposited on a substrate obtained upon NIR excitation between 800 and 1000 nm on a Nikon setup. The spectra at each wavelength are associated with the emission of the object indicated by the dashed outline in the exemplary image obtained upon 1000 nm excitation (further spectral characterizations are provided in Supporting Information S-43, Figure S13). In the lower row, we present a series of nonlinear microscopy images of DU-145 cells treated with **LNO-CM-ELA** NPs (50 μg/mL) acquired on a Leica setup. Upon excitation at 800 nm, the second harmonic generation (SHG) is visible in the 400 nm channel and the 4′,6-diamidino-2-phenylindole (DAPI) fluorescence from the nuclei is visible in the channel centered at 495 nm. Using the 1250 nm excitation, the system is capable to acquire both the third harmonic generation (THG) at 416 nm and the SHG at 625 nm (1/2 and 1/3 of the excitation wavelength, respectively). The comparison between the three harmonic responses highlights HNP excitation tunability beyond the NIR range. The labeling of cells is quite sparse as HNPs appear as isolated aggregates but overall sufficient for recognizing cell morphologies.

### NIR-Triggered Release of **ELA** and in vitro Evaluation of Cytotoxic Activity

**Figure 3:**
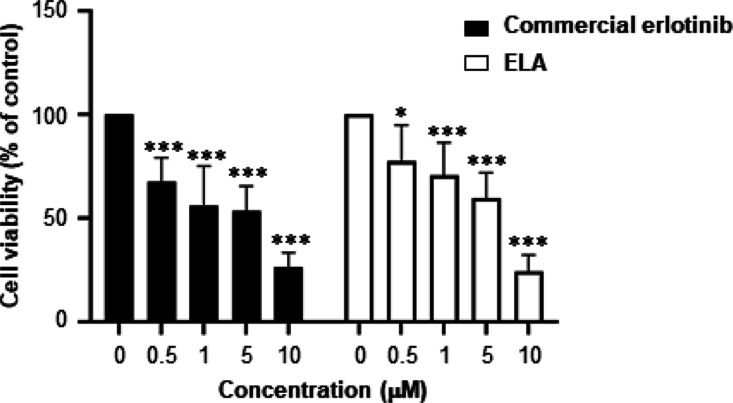
Evaluation of the cytotoxic effect of **ELA** and commercial erlotinib on DU145 cells. DU145 cells were incubated with increasing concentrations of **ELA** or commercial erlotinib for 72 h. Cell viability was assessed using MTT colorimetric assay. Results are expressed as the mean ± SD of two independent experiments. Statistical differences were calculated by two-way ANOVA. *p < 0.05, **p < 0.01, and ***p < 0.001 compared to the control.

The erlotinib derivative **ELA** was first evaluated for its cytotoxic effect on the EGFR-overexpressing DU-145 human androgen-dependent prostate cancer cells (for EGFR expression in DU-145 cells, see Supporting Information S34, Figure S4), in comparison with the parent commercial drug. DU-145 cells were incubated with commercial erlotinib for 72 h using concentrations ranging from 0.5 to 10 μM following the protocol described in the experimental section. The impact on cell viability was assessed using 3-(4,5-dimethyl-2-thiazoyl)-2,5-diphenyltetrazolium bromide (MTT) colorimetric assay. Our findings indicate that the two compounds displayed a similar cytotoxicity profile as they were able to induce 40–57% growth inhibition at 1 μM and 88–90% at 10 μM (Figure 3). These results suggest that the erlotinib derivative **ELA** conserves the ability to inhibit the growth of EGFR-overexpressing cancer cells.

**Figure 4:**
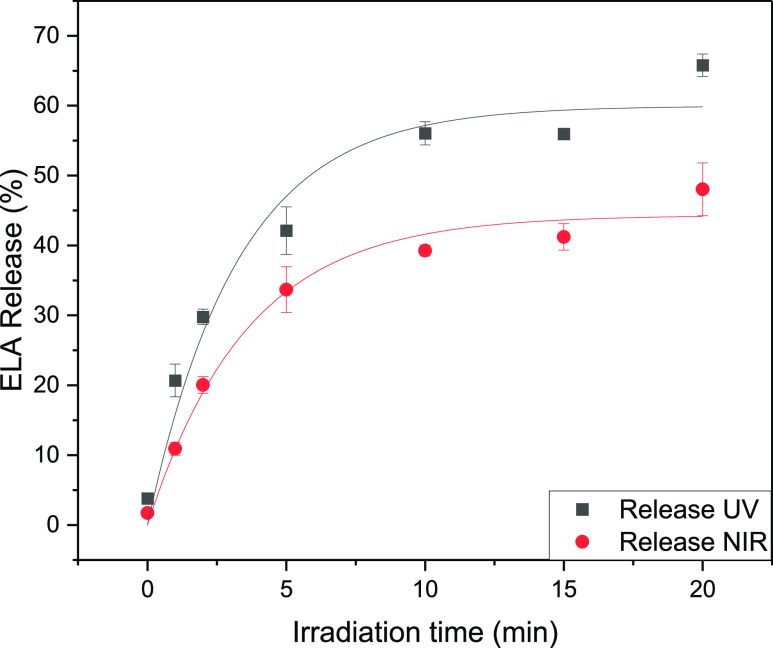
Release of **ELA** from **LNO-CM-ELA** NPs (150 μg/mL in PBS) upon UV (366 nm) and NIR (790 nm) irradiation. The curves were fitted with a monoexponential function.

The photocleavage and subsequent release of **ELA** from the surface of functionalized LNO NPs was first assessed under direct UV irradiation (see Supporting Information S35 for an estimation of UV flux at the sample) at 366 nm in PBS, in the absence of cancer cells (Figure 3, black curve, see Supporting Information S35 for detailed calculation and progress curve). Analysis of the medium by ultrahigh-performance liquid chromatography–mass spectrometry (UHPLC–MS) at successive time points indicated that the unique released component was **ELA** (assessed by its coelution with the standard after 2.0 min and accurate mass, see Supporting Information S38 for quantification protocols and calibration curve). Then, the ability of the second harmonic emission of **LNO-CM-ELA** NPs to trigger the uncaging of the cytotoxic cargo was investigated using the unfocused beam from an amplified Ti:sapphire pulsed laser system. In the first setting, we used an amplified laser system delivering a femtosecond pulse train at a 1 kHz repetition rate (setup 1, see Experimental Section) according to the protocols previously developed on BFO NPs.^45^ The choice of an amplified laser system at a low repetition rate allows to maintain a comparatively large peak intensity (necessary for exerting nonlinear interaction) on a large sample area, which facilitates the subsequent reliable quantification of **ELA** release by UHPLC–MS. The low repetition rate is also advantageous for limiting heat accumulation effects and sample photodamage.^50^ With setup 1, after 15 min of irradiation at 790 nm in PBS, the release of **ELA** reached 41% (Figure 4). This irradiation induced an SHG emission at 395 nm from the HNPs, matching with the absorption of amino-coumarin dyes reported to be centered around 380 nm.^51^ The progress curves were fitted using a monoexponential function, and the initial rate (*k*_0_) at 5 min was calculated to be 271 nM·min^–1^ (see Supporting Information S36 for the detailed calculation and progress curve).

In order to verify the dependence of SHG wavelength on the release, given that setup 1 works at fixed wavelength, we repeated the irradiation on a tunable MHz laser (setup 2, see Experimental Section). Because of the inherent characteristics of this source, we were bound to work using different peak intensities and irradiation surfaces. To partially compensate for these discrepancies, we applied longer irradiation times and we continuously moved the sample during the irradiation as detailed in the Experimental Section. We applied the irradiation treatment at three excitation wavelengths (800, 1000, and 1100 nm) for 1 h. Although the results from UHPLC–MS are less quantitatively reliable under these irradiation conditions, we could only observe **ELA** release upon 800 nm irradiation (corresponding to SHG emission within the absorption spectrum of CM) and basically no **ELA** detection at the two other wavelengths (Supporting Information S41, Figure S11).

Under NIR irradiation, the observed release rate was slower as compared to direct UV treatment as the efficiency of SHG to generate the cleaving wavelength has to be taken into account. However, the concentration of **ELA** reached under these conditions was in the expected range to exert cytotoxic effects on cancer cells.

Different control experiments were performed to verify that the **ELA** release resulted from the photocleavage of the carbamate bond of **CM-ELA**, primarily induced by the SHG from the HNP core (Table 2). In addition, the synthesis of LNO nanocrystals was adjusted to produce bare LNO NPs of around 100 nm mean diameter, in order to increase the SHG emission intensity and resulting uncaging capacity. Following silanization and post-conjugation to **DIBO-CM-ELA** and **DIBO-ELA**, the resulting functionalized HNPs presented **ELA** loading values of 30 and 38 nmol/mg for **LNO-CM-ELA** and **LNO-ELA** NPs, respectively. First, these **LNO** derivatives were irradiated for 15 min at 790 nm, leading to 70.1 and 7.4% release of the cargo, respectively. Then, the release of **ELA** was quantified from **LNO-CM-ELA** NPs, kept in the dark for 15 min at 22 °C (6.6% release), or heated at 50 °C up to 30 min (8% release). Under NIR irradiation, the temperature increase induced by exposure to the laser excitation was estimated well below this value (see Supporting Information S40, Figure S10). The release from **DIBO-CM-ELA**, non-conjugated to the surface of LNO NPs, was also evaluated. The cargo release resulting from direct two-photon absorption of the photocleavable coumarinyl linker contributed to 37% of the release with respect to conjugated **LNO-CM-ELA** NPs. This value is not surprisingly higher than what was previously observed for BFO NPs^45^ as LNO has a smaller nonlinear optical efficiency,^34^ which is compensated by better size and morphology dispersion properties, leading to more robust functionalization strategies.

**Table 2:** **ELA** Release Control Studies (Setup 1)^a^

sample	conditions	**ELA** release (nM)	release SD (nM)	% of release
**LNO-CM-ELA** NPs	790 nm irradiation, 15 min	631.1	4.4	70.1
**LNO-ELA** NPs	790 nm irradiation, 15 min	66.8	6.9	7.4
**DIBO-CM-ELA**^b^	790 nm irradiation, 15 min	161.6 (*231*)	14.6	18.0 (*26*)
**LNO-CM-ELA** NPs	no irradiation, 22 °C	59.6	5.8	6.6
**LNO-CM-ELA** NPs	no irradiation, 50 °C, 30 min	71.8	7.0	8.0

a The NP concentration was kept at 150 μg/mL in PBS in all experiments.

b This control was performed at a different laser average power (4.1 instead of 4.9 W, same beam geometry and pulse duration). The values **corrected** taking into account squared intensity dependence are in italic.

As one can observe, only **LNO-CM-ELA** NPs, exposed to NIR irradiation, induced the release of **ELA** at a concentration prone to exert cytotoxic effects on cancer cells. These control experiments confirmed that the cargo release took place upon cleavage of the photosensitive linker when being exposed to the proper irradiation and that any potential temperature increase induced by the irradiation did not contribute to the uncaging process.

**Figure 5:**
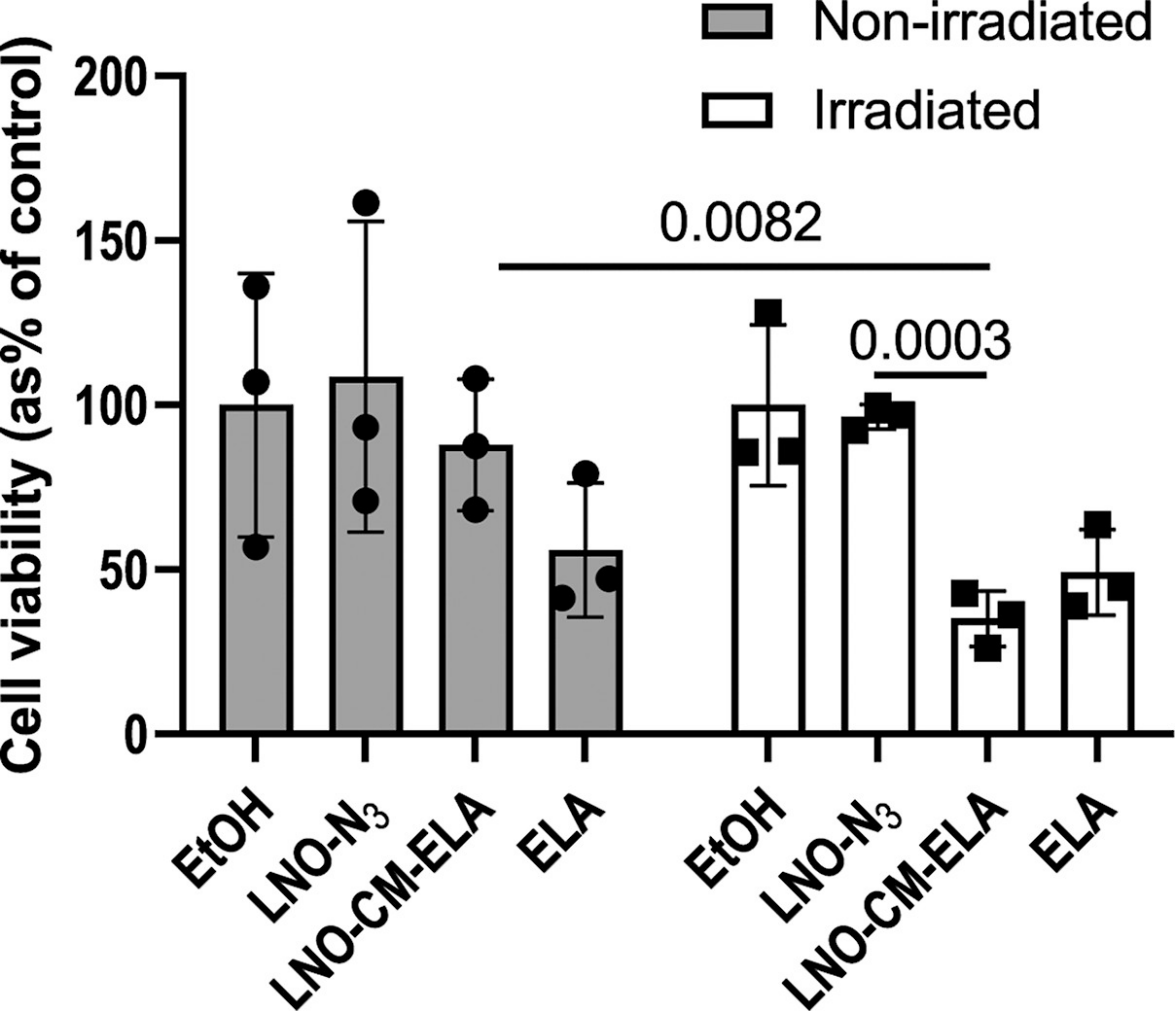
Effect of the NIR-triggered release of **ELA** on the viability of DU145 cells. Cells were incubated with **LNO-N**_**3**_ NPs (150 μg/mL), **LNO-CM-ELA** NPs (150 μg/mL), or **ELA** (0.98 μM) for 24 h, followed by 15 min femtosecond laser pulsed irradiation at 790 nm. Cell viability was evaluated using MTT assay 72 h after exposure to the laser source (gray bars). The same protocol was performed, in parallel, without laser exposure (black bars). Results are expressed as the mean ± SD of three independent experiments. Differences between irradiated and non-irradiated samples and between cells exposed to **LNO-N**_**3**_ and **LNO-CM-ELA** NPs were statistically analyzed by a two-way ANOVA test: **p < 0.01 and ***p < 0.001.

The release pattern of **ELA** upon NIR irradiation of functionalized NPs and the cargo concentration reached after 15 min of exposure to the laser source prompted us to further explore the ability of **LNO-CM-ELA** NPs to exert cytotoxic effect on DU145 cells, under similar conditions. The cells were incubated with **LNO-CM-ELA** NPs for 24 h and subsequently irradiated at 790 nm for 15 min. Cell viability was evaluated 72 h after the light exposure treatment (Figure 5). Irradiation of DU-145 cells exposed to **LNO-CM-ELA** resulted in 66% inhibition of cell growth, an effect comparable to the growth inhibitory action induced by 0.98 μM **ELA** (Figure 3). The cell viability observed was slightly lower in the case of treatment with **LNO-CM-ELA** NPs as compared to incubation with **ELA**. This observation is consistent with the calculated concentration of **ELA** released from **LNO-CM-ELA** NPs at a concentration of 150 μg/mL, which was estimated to reach 1.6 μM. Importantly, no detrimental effect was observed for non-irradiated cells or for irradiated cells incubated with **LNO-N**_**3**_ NPs. This result suggests that **LNO-N**_**3**_ and **LNO-CM-ELA** NPs do not have intrinsic cytotoxic properties and that the release of **ELA** upon NIR irradiation from loaded HNPs can efficiently inhibit the growth of EGFR-overexpressing prostate cancer cells.

The uncaging of chemotherapeutic compounds from the surface of functionalized inorganic NPs through photocleavable linkers was already reported for other types of materials.^52^ Most of the previous studies made use of UCNPs while HNPs represent more recent systems investigating such an approach. The most frequently reported drug model is doxorubicin, which induces much more aggressive effects than erlotinib as it causes cell apoptosis.^53^ In particular, the NIR-triggered release of doxorubicin from UCNPs was presented by Wong et al.^54^ and resulted in cancer cell growth inhibition comparable to the effect achieved with **LNO-CM-ELA** NPs. However, their protocol required longer irradiation time (60 min) and significantly higher concentration of functionalized UCNPs (750 μg/mL) to trigger efficient reduction in cell viability. To the best of our knowledge, the study herein presented is the first example of the successful NIR-triggered photo-release of a TK inhibitor such as erlotinib from surface-functionalized inorganic NPs.

## Conclusions

Nonlinear optical parametric signals from nanoparticles such as harmonic generation and frequency mixing, present appealing properties for imaging applications at longer wavelengths complementary to those of other fluorescence or luminescence nanoparticle probes.^55^ By means of advanced surface functionalization protocols, additional modalities, such as the controlled uncaging of molecular payloads, can be activated by simple tuning of the wavelength of the pulsed laser excitation. This procedure is automatic on a number of commercial laser systems nowadays. The combined results herein presented demonstrate the multi-harmonic imaging and NIR-triggered delivery of a chemotherapeutic compound targeting EGFR-overexpressing cancer cells. A cytotoxic compound derived from the EGFR TK inhibitor erlotinib was synthesized to allow further conjugation to the surface of LNO HNPs displaying surface-reactive azide functionalities, while preserving the growth inhibitory effect of the parent drug molecule on human prostate DU145 cancer cells. Caging with a photosensitive coumarinyl-based spacer, followed by covalent immobilization on coated **LNO-N**_**3**_ NPs through copper-free azide for alkyne click [3 + 2] cycloaddition, provided functionalized HNPs at a loading rate of 27 nmol/mg LNO. The colloidal stability of **LNP-CM-ELA** NPs upon storage in PBS buffer (pH 7.4) at 4 °C was confirmed up to 7 days. However, some aggregation was observed in cell culture medium [Dulbecco’s modified Eagle’s medium (DMEM) containing 10% FCS], and further investigation on the chemical composition of the surface functionalization pattern will aim at reducing the tendency to aggregate in cell culture medium. **LNO-CM-ELA** NPs showed significant labeling potential on DU145 cancer cells, and their simultaneous emission of SHG and THG upon long-wavelength excitation can be exploited to image them selectively against endogenous emission (autofluorescence, collagen SHG, and so on). Adjusting the incoming irradiation to 790 nm for 15 min activated the uncaging process and resulted in the efficient release of **ELA**, triggered by the SHG emission at 395 nm by the NPs. Under these conditions, the cytotoxic effect of the released cargo was evidenced by a significant decrease in the viability of the EGFR-overexpressing DU145 cells, comparable to the effect of the free molecule. Noteworthy, in the absence of irradiation, **LNO-CM-ELA** NPs did not exert detrimental effects on the cell viability according to MTT assay. Also, DU145 cells were not affected by incubation with non-functionalized **LNO-N**_**3**_ NPs, with or without exposure to laser excitation at 790 nm. The functionalized HNPs herein disclosed stand as promising nanoplatforms, suitable for decoupled harmonic imaging and localized on-demand drug delivery.

## Experimental Section

### Release of **ELA** under UV-A Irradiation

UV-induced photolysis experiments were performed with a Sylvania UV light tube (366 nm, 8 W). **LNO-CM-ELA** NPs (150 μg) were suspended in PBS (1 mL; pH = 7.4, 144 mg/L KH_2_PO_4_, 9000 mg/L NaCl, and 795 mg/L Na_2_HPO_4_·7H_2_O) and placed in eight-well borosilicate cover glass polystyrene chambers (Nunc Lab-Tek II, Merck) for irradiation at 366 nm (see Supporting Information S34 for an estimation of UV flux at the sample). Aliquots (40 μL) of the suspension were withdrawn at the indicated time points, diluted with PBS to 200 μL to achieve a 1:5 dilution ratio, and centrifuged (10 min, 13,000 rpm). Quantification of **ELA** in the supernatant (triplicates) was performed by UHPLC–MS. Details of the parameters for UHPLC–ESI–HRMS analysis and calibration curves are provided in Supporting Information (S37, Figure S7).

### Release of **ELA** upon NIR Femtosecond Pulsed Laser Irradiation

#### Irradiation Setup 1

In this case, the irradiation source is an amplified Ti:Sapphire laser system (Astrella, Coherent) with 4.9 W average output at a 1 KHz repetition rate. The system delivers pulses centered at 790 nm with 35 nm bandwidth corresponding to 27 fs transform-limited duration. The actual pulse duration measured using a commercial second harmonic generation frequency resolved optical gating (SHG-FROG) device (PulseCheck, APE Berlin) yields approximately 60 fs. The beam diameter, determined using a beam profiler (Newport) set at the amplifier output, corresponds to approximately 11 mm at 1/*e*^2^. The beam is directed on the bottom surface of the multi-well plate using a 45° dielectric mirror, without focusing. The peak intensity at the sample corresponds to 87 GW/cm^2^ at 1/*e*^2^ (Supporting Information S39, Figure S8). **LNO-CM-ELA** NPs (150 μg) were suspended in PBS (1 mL; pH = 7.4, 144 mg/L KH_2_PO_4_, 9000 mg/L NaCl, and 795 mg/L Na_2_HPO_4_·7H_2_O) and placed in eight-well borosilicate cover glass polystyrene chambers (Nunc Lab-Tek II, Merck). The samples were irradiated at 790 nm.

#### Irradiation Setup 2

The setup for comparing the release of **ELA** at different wavelengths is based on a 680–1300 nm tunable ultrafast system (InSight X3, Spectra-Physics) with 1.4 W average output power at an 80 MHz repetition rate. The nominal pulse duration is approximately 120 fs. The experiment was performed at 800, 1000, and 1100 nm. The pulse temporal compression was carefully adjusted to obtain Fourier-limited duration for each condition. The beam diameter, determined using a beam profiler (Newport) set at the laser output, corresponds to approximately 1.36 mm at 1/*e*^2^. The beam is directed on the bottom surface of the multi-well plate with a 45° dielectric mirror, without focusing. The peak intensity at the sample corresponds to 10 MW/cm^2^ at 1/*e*^2^. To account for the smaller beam size compared to the size of the well (12 mm), we used a rotational motor (PRM1-Z8—Thorlabs) to move continuously the sample with respect to the beam position in a circular pattern (2π/10 min). To partially compensate for the lower peak intensity with respect to setup 1, we applied the irradiation for a longer time span (1 h instead of 15 min).

Aliquots (40 μL) of the suspension were withdrawn at the indicated time points, diluted with PBS to 200 μL to achieve a 1:5 dilution ratio, and centrifuged (10 min, 13,000 rpm). Quantification of **ELA** in the supernatant (triplicates) was performed by UHPLC–MS. Details of the parameters for UHPLC–ESI–HRMS analysis and calibration curves are provided in Supporting Information (S35, Figure S7).

### In Vitro Evaluation of the Cytotoxic Effect of Erlotinib and **ELA**

DU145 cells cultured in DMEM containing 10% of FCS were exposed to increasing concentration of either **ELA** or commercial erlotinib for 72 h at 37 °C. Cells were then washed two times with PBS and incubated for 2 h with a solution of MTT in DMEM containing 10% FCS without phenol red. Cells were then lysed by incubating them for 15 min with isopropanol/HCl 0.04 M. After homogenization, absorbance was measured at 540 nm using an Epoch 2 microplate spectrophotometer (BioTek). Experiments were conducted in triplicates and repeated two times. Means ± standard deviation (SD) were calculated.

### In Vitro Evaluation of the Cytotoxic Effect of the NIR-Triggered Release of **ELA**

**LNO-CM-ELA** NPs or **LNO-N**_**3**_ NPs (10 mg/mL in EtOH) were diluted to 0–200 μg/mL with DMEM containing 10% of FCS and added to eight-well borosilicate cover glass polystyrene chambers (Nunc Lab-Tek II, Merck) containing DU145 cells in DMEM (400 μL). Cells were incubated with **LNO-N**_**3**_ NPs (150 μg/mL), **LNO-CM-ELA** NPs (150 μg/mL), **ELA** (0.98 μM), or DMEM containing 1.5% EtOH as the control. After 24 h incubation at 37 °C, the samples were irradiated at 790 nm for 15 min using the setup described above. The samples were incubated at 37 °C for 72 h. Cells were washed with PBS and incubated with a solution of MTT in DMEM containing 10% FCS without phenol red (1 mM), for 2 h. The supernatant was discarded, and cells were lysed with isopropanol/HCl 40 mM. Absorbance at 540 nm was measured using an Epoch 2 microplate spectrophotometer (BioTek) and compared to the values of control cells incubated in only DMEM containing 1.5% EtOH. Experiments were conducted in triplicate wells and repeated three times. Means ± SD were calculated.

### Nonlinear Imaging

#### Cells

DU145 human prostate cancer cells were grown in DMEM medium supplemented with GlutaMAX (Gibco N 61870036), non-essential amino acids (Gibco N 1140035), 10% heat-inactivated fetal bovine serum (FBS) (Gibco N 10270106), and gentamycin (100 μg/mL) (Gibco N 15750045). Cells were plated in 35 mm Petri dishes with a glass bottom (MatTek, P35G-0.170-14-C) for 24 h, and then, cell layers were washed once with PBS and **LNO-CN-ELA** NPs (50 μg/mL) in DMEM medium without FCS were added for 24 h. After 48 h of incubation, the cell layers were washed twice with PBS, fixed for 20 min using 4% paraformaldehyde, and finally stained with DAPI (Roth, 6335.1) (1:5000) for 30 min and kept at 4 °C until the imaging session.

Cell images presented in Figure 2 were acquired on a Leica SP8 DIVE FALCON upright multiphoton microscope coupled with a tunable femtosecond laser (InSight X3, Newport Spectra- Physics) using a NA 0.95 water-immersion objective (Leica HC FLUOTAR L 25×). The acquisition spectral range for each channel was selected using a Leica 4Tune system. Images at each excitation wavelength (SHG and DAPI fluorescence at 800 nm and SHG and THG at 1250 nm) were acquired simultaneously. The gains in detectors were adjusted for each signal.

#### Nanoparticles

The spectral response upon NIR excitation of **LNO-CN-ELA** NPs was performed on a different microscopy setup (Nikon MP) characterized by a narrower excitation tunability range (750–1000 nm) but capable of hyperspectral imaging over 32 independent detection channels. From a colloidal dispersion of the compound in EtOH (2 mg/mL), an aliquot of 2.5 μL was drop-cast on a microscope coverslip. After solvent evaporation, the sample was imaged on a Nikon multiphoton inverted microscope (A1R-MP) coupled with a 80 MHz Ti:Sapphire oscillator (Mai-Tai, Newport-Spectra-Physics) through a water immersion objective (Nikon, Plan Apo IR 60×, NA 1.27). The epi-collected signal was acquired using the Nikon spectral unit (max range 400–650 nm) with grating selection being set to 6 nm resolution.

#### Synthesis Protocols

Designation of the compounds refers to the chemical structures presented in Schemes 1 and 2. Details on the experimental protocols and instrumentation used for characterization are given in Supporting Information, S3.

##### Preparation of Compound **2**

To a solution of **1** (10.02 g, 20.0 mmol, 1.0 equiv) in toluene (120 mL) were added DMF-DMA (10.6 mL, 80.0 mmol, 2.0 equiv) and AcOH (0.7 mL, 12.0 mmol, 0.3 equiv). The reaction mixture was refluxed for 2 h. The mixture was cooled to rt and concentrated under reduced pressure. The resulting oil was triturated with heptane and sonicated. The precipitate was filtered and washed with heptane to afford **2** as a pale-yellow solid (9.45 g, 30.9 mmol, 77% yield). ^1^H NMR (400 MHz, chloroform-*d*): δ *7*.55 (s, 1H, *N*=CH), 6.92 (s, 1H, *H*_Ar_), 6.46 (s, 1H, *H*_Ar_), 4.42 (t, *J* = 4.6 Hz, 2H, C*H*_2_–OAr), 4.21 (t, *J* = 4.8 Hz, 2H, C*H*_2_–OAc), 3.80 (s, 3H, C*H*_3_–OAr), 3.80 (s, 6H, 2 × C*H*_3_–*N*), 2.06 (s, 3H, C*H*_3_–CO) ppm. ^13^C NMR (101 MHz, chloroform-*d*): δ 170.9, 153.8, 151.8, 150.2, 143.7, 119.7, 115.0, 105.6, 96.0, 66.8, 62.4, 56.4, 40.3, 34.6, 20.9 ppm. HRMS (ESI/QTOF): *m/z* calcd for C_15_H_19_N_3_O_4_^+^ ([M + H]^+^), 306.1453; found, 306.1454. ^1^H NMR, ^13^C NMR spectra, IR data (Supporting Information S17).

##### Preparation of Compound **3**

3-Ethynylaniline (17 mmol, 1.9 mL, 1.1 equiv) was added to a solution of **2** (15.3 mmol, 4.68 g, 1.0 equiv) in AcOH (40 mL). The reaction mixture was stirred at 130 °C for 2 h. The solution was concentrated, and the residue was recrystallized twice from toluene to afford **3** as a beige powder (12.0 mmol, 4.54 g, 68%). ^1^H NMR (400 MHz, dimethyl sulfoxide-*d*_6_): δ 9.53 (s, 1H, NH), 8.51 (s, 1H, *H*_Ar_), 7.99 (t, *J* = 1.6 Hz, 1H, *H*_Ar_), 7.90 (dd, *J* = 8.0, 1.9 Hz, 1H, *H*_Ar_), 7.86 (s, 1H, *H*_Ar_), 7.41 (t, *J* = 7.9 Hz, 1H, *H*_Ar_), 7.24 (s, 1H, *H*_Ar_), 7.21 (m, 1H, *H*_Ar_), 4.41 (m, 2H, C*H*_2_–OAc), 4.36 (m, 2H, C*H*_2_–OAr), 4.21 (s, 1H, *H*C≡C), 3.98 (s, 3H, C*H*_3_–OAr), 2.07 (s, 3H, C*H*_3_–CO) ppm. ^13^C NMR (101 MHz, dimethyl sulfoxide-*d*_6_): δ 170.9, 156.6, 153.6, 152.6, 149.9, 147.2, 139.0, 129.2, 126.9, 125.2, 122.8, 121.8, 109.9, 108.7, 101.8, 84.4, 80.8, 67.1, 62.7, 55.3, 21.4 ppm. HRMS (ESI/QTOF): *m/z* calcd for C_21_H_19_N_3_O_4_^+^ ([M + H]^+^), 378.1453; found, 378.1454. ^1^H NMR, ^13^C NMR spectra, IR data (Supporting Information S18).

##### Preparation of Compound **4**

K_2_CO_3_ (12.0 g, 87.1 mmol, 5.0 equiv) was added to a solution of **4** (6.58 g, 17.4 mmol, 1.0 equiv) dissolved in MeOH (400 mL). The reaction mixture was stirred at rt for 20 min and diluted with H_2_O (100 mL). MeOH was evaporated under reduced pressure. The precipitate was filtered and washed with H_2_O to afford the intermediate alcohol as a yellow solid (5.7 g, 16.9 mmol, 98% yield). Mesyl chloride (0.12 mL, 1.49 mmol, 5.0 equiv) was added dropwise to a stirred solution of the above intermediate (100.0 mg, 0.30 mmol, 1.0 equiv) and DMAP (18.2 mg, 0.15 mmol, 0.5 equiv) in dry pyridine (5 mL), at 0 °C. The reaction mixture was warmed to rt and stirred for 5 h under an argon atmosphere. Completion of the reaction was monitored by ESI–MS. A saturated aqueous NaHCO_3_ solution (15 mL) was added to the reaction mixture. The aqueous layer was extracted with EtOAc (30 mL, five times). The combined organic layers were dried over MgSO_4_, filtered, and concentrated under reduced pressure to afford the corresponding mesylate as a yellow solid (114.8 mg, 0.28 mmol, 93% yield). This intermediate (105 mg, 0.25 mmol, 1.0 equiv) and NaI (0.45 g, 2.53 mmol, 10.0 equiv) were dissolved in acetone (5 mL) in a sealed tube, and the solution was stirred at 80 °C for 16 h. The mixture was filtered, the solvent was removed under reduced pressure, and the crude product was purified by flash column chromatography (FCC) (EtOAc) to afford **4** as a yellow solid (92.8 mg, 0.21 mmol, 62% over three steps). ^1^H NMR (400 MHz, chloroform-*d*): δ 8.60 (s, 1H, *H*_Ar_), 7.86 (t, *J* = 1.8 Hz, 1H, *H*_Ar_), 7.77 (dt, *J* = 8.2, 1.5 Hz, 1H, *H*_Ar_), 7.35 (t, *J* = 7.9 Hz, 1H, *H*_Ar_), 7.31–7.27 (m, 1H, *H*_Ar_), 7.24 (s, 1H, *H*_Ar_), 7.17 (s, 1H, *H*_Ar_), 4.40 (t, *J* = 7.1 Hz, 2H, C*H*_2_–OAr), 4.04 (s, 3H, C*H*_3_–OAr), 3.55–3.46 (m, 2H, C*H*_2_–I), 3.10 (s, 1H, *H*C≡C) ppm. ^13^C NMR (101 MHz, chloroform-*d*): δ 168.5, 156.3, 153.6, 150.0, 143.7, 138.4, 129.2, 128.4, 125.5, 123.1, 122.8, 108.1, 100.6, 83.3, 77.8, 69.6, 57.0, −0.5 ppm. HRMS (ESI/QTOF): *m/z* calcd for C_19_H_17_IN_3_O_2_^+^ ([M + H]^+^), 446.0360; found, 446.0360. ^1^H NMR, ^13^C NMR spectra, IR data (Supporting Information S19).

##### Preparation of **ELA**

Compound **4** (92.8 mg, 0.21 mmol, 1.0 equiv) was dissolved in 7N ammonia (7N in MeOH, 3 mL) in a sealed tube. The solution was stirred at 85 °C for 16 h. The solvent was removed under reduced pressure to afford **ELA** (70.2 mg, 0.21 mmol, quant.) as a yellow solid. ^1^H NMR (400 MHz, methanol-*d*_4_): δ 8.51 (s, 1H, *H*_Ar_), 7.94 (t, *J* = 1.9 Hz, 1H, *H*_Ar_), 7.90 (s, 1H, *H*_Ar_), 7.80 (dd, *J* = 8.2, 2.3 Hz, 1H, *H*_Ar_), 7.41 (t, *J* = 7.9 Hz, 1H, *H*_Ar_), 7.31 (dt, *J* = 7.6, 1.3 Hz, 1H, *H*_Ar_), 7.26 (s, 1H, *H*_Ar_), 4.49–4.40 (m, 2H, C*H*_2_–OAr), 4.12 (s, 3H, C*H*_3_–OAr), 3.55 (s, 1H, *H*C≡C), 3.53–3.47 (m, 2H, C*H*_2_–NH_2_). ^13^C NMR (101 MHz, methanol-*d*_4_): δ 160.1, 155.9, 153.4, 152.6, 149.6, 141.0, 128.6, 127.7, 125.9, 123.1, 122.9, 121.2, 107.1, 101.9, 82.8, 77.4, 65.2, 55.7, 38.7. HRMS (ESI/QTOF): *m/z* calcd for C_19_H_19_N_4_O_2_^+^ ([M + H]^+^), 335.1503; found, 335.1511. ^1^H NMR, ^13^C NMR spectra, IR data (Supporting Information S23).

##### Preparation of Compound **6**

To a solution of **ELA** (71.2 mg, 0.21 mmol, 1.0 equiv) and compound **5** (100.0 mg, 0.21 mmol, 1.0 equiv) in dry DMF (10 mL), ^*i*^Pr_2_NEt (0.15 mL, 0.85 mmol, 4.0 equiv) was added. The solution was stirred at rt for 16 h under an argon atmosphere and dark conditions. The solvent was removed under reduced pressure, and the crude product was purified by FCC (PE/EtOAc, 5:5 to 0:1) to afford **6** as a yellow solid (139.1 mg, 0.21 mmol, quant.). ^1^H NMR (400 MHz, dimethyl sulfoxide-*d*_6_): δ 9.51 (s, 1H, NH_ELA_), 8.50 (s, 1H, *H*_Ar_), 7.99 (d, *J* = 1.9 Hz, 1H, *H*_Ar_), 7.90 (m, 2H, *H*_Ar_), 7.81 (t, *J* = 5.6 Hz, 1H, N*H*_carbamate_), 7.48 (m, 1H, CM-*H*), 7.41 (t, *J* = 7.8 Hz, 1H, *H*_Ar_), 7.22 (dd, *J* = 16.9, 9.5 Hz, 2H, *H*_Ar_), 6.64 (dd, *J* = 9.1, 2.6 Hz, 1H, CM-*H*), 6.53 (d, *J* = 2.5 Hz, 1H, CM-*H*), 6.05 (s, 1H, CM-*H*), 5.25 (s, 2H, C*H*_2__CM_), 4.28 (s, 2H, *N*–C*H*_2_–CO_2_Et), 4.23 (t, *J* = 5.5 Hz, 2H, NH–C*H*_2_–CH_2_–OAr), 4.20 (s, 1H, *H*C≡C), 4.13 (q, *J* = 7.2 Hz, 2H, C*H*_2__ester_), 3.97 (s, 3H, C*H*_3_–OAr), 3.49 (d, *J* = 5.0 Hz, 4H, *N*–C*H*_2_–CH_3_ and NH–CH_*2*_–C*H*_2_–OAr), 1.20 (m, 3H, *N*–CH_2_–C*H*_3_), 1.12 (t, *J* = 7.0 Hz, 3H, C*H*_3__ester_). ^13^C NMR (101 MHz, dimethyl sulfoxide-*d*_6_): δ 170.5, 161.1, 156.6, 155.8, 153.8, 153.2, 152.2, 151.4, 149.5, 147.4, 140.3, 129.3, 126.8, 125.7, 125.2, 123.1, 122.2, 109.5, 108.5, 106.7, 106.5, 105.3, 102.7, 98.0, 84.0, 81.0, 67.6, 61.5, 61.1, 56.8, 51.9, 46.2, 40.2, 14.6, 12.5. HRMS (ESI/QTOF): *m/z* calcd for C_36_H_36_N_5_O_8_^+^ ([M + H]^+^), 666.2558; found, 666.2566. ^1^H NMR, ^13^C NMR spectra, IR data (Supporting Information S25).

##### Preparation of **CM-ELA**

To a solution of **5** (140 mg, 0.21 mmol, 1.0 equiv) in MeOH/H_2_O/DMF (8 mL, 5:1:1), LiOH (100 mg, 4.2 mmol, 20.0 equiv) was added, and the reaction mixture was stirred at rt for 6 h under dark conditions. The solution was diluted with H_2_O (50 mL) and 1 M aqueous HCl (8 mL). The aqueous layer was extracted with EtOAc (60 mL, three times), and the combined organic layers were dried over MgSO_4_, filtered, and concentrated under reduced pressure. The residue was purified by FCC (DCM/MeOH, 9:1) to afford the corresponding carboxylic acid as a pale-yellow solid (102 mg, 0.16 mmol, 89%). To a solution of this intermediate (25.0 mg, 39.2 μmol, 1.0 equiv) and **7** (13.2 mg, 43.1 μmol, 1.1 equiv) in dry DMF (2.5 mL) were added DMAP (2.4 mg, 19.6 μmol, 0.5 equiv), HOBt (21.2 mg, 0.16 mmol, 4.0 equiv), and EDCI (30.1 mg, 0.16 mmol, 4.0 equiv). ^*i*^Pr_2_NEt (27 μL, 0.16 mmol, 4.0 equiv) was added, and the solution was stirred at rt for 24 h under an argon atmosphere and dark conditions. The solvent was removed under reduced pressure, and the crude product was purified by preparative thin-layer chromatography (DCM/MeOH, 18:1) to afford **CM-ELA** as a yellow solid (14.7 mg, 15.8 μmol, 36% over two steps). ^1^H NMR (400 MHz, dimethyl sulfoxide-*d*_6_): δ 9.54 (s, 1H, NH_ELA_), 9.45 (d, *J* = 7.9 Hz, 1H, N*H*_amide_), 8.50 (d, *J* = 5.4 Hz, 1H, *H*_Ar_), 7.99 (d, *J* = 12.5 Hz, 1H, *H*_Ar_), 7.94–7.86 (m, 2H, *H*_Ar_), 7.81 (d, *J* = 8.4 Hz, 1H, *H*_Ar_), 7.60 (dt, *J* = 11.5, 5.7 Hz, 1H, N*H*_carbamate_), 7.56–7.49 (m, 1H, CM*-*H and *H*_Ar-cyclooctyne_), 7.47–7.27 (m, 7H, *H*_Ar_), 7.26–7.18 (m, 2H, *H*_Ar_), 6.56 (dd, *J* = 21.0, 8.6 Hz, 1H, CM*-H*), 6.51–6.40 (m, 1H, CM*-H*), 6.04 (d, *J* = 3.1 Hz, 1H, CM*-H*), 5.27 (m, 2H, N*H*_carbamate_ and *H*_DIBO_), 5.23 (s, 2H, C*H*_*2*_), 4.39 (t, *J* = 5.6 Hz, 1H, NH–C*H*_*2*_–CH_2_-OAr), 4.23 (t, *J* = 5.9 Hz, 1H, NH–C*H*_*2*_–CH_2_-OAr), 4.20 (d, *J* = 5.1 Hz, 1H, *H*_alkyne_), 3.97 (s, 3H, C*H*_*3*_–O), 3.96–3.84 (m, 4H, *N*–C*H*_*2*_–CONHR and NH–CH_*2*_–C*H*_2_–OAr), 3.58–3.47 (m, 2H, *N*–C*H*_*2*_–CH_3_), 3.17 (q, *J* = 6.3, 5.6 Hz, 3H, NH–C*H*_*2*_–CH_2_–NHCO_2_R and *H*_DIBO_), 3.06 (q, *J* = 7.6, 6.8 Hz, 2H, NH–CH_2_–C*H*_*2*_–NHCO_2_R), 2.75 (dd, *J* = 15.0, 4.4 Hz, 1H, *H*_DIBO_), 1.06 (t, *J* = 7.0 Hz, 1H, R_2_N–CH_2_–C*H*_*3*_). ^13^C NMR (101 MHz, dimethyl sulfoxide-*d*_6_): δ 171.2, 169.4, 159.8, 156.6, 155.8, 155.8, 155.8, 153.2, 152.1, 151.3, 149.5, 142.8, 146.1, 140.3, 129.3, 128.9, 127.8, 127.8, 126.5, 126.2, 125.2, 124.3, 123.9, 123.4, 123.0, 122.2, 120.8, 119.0, 113.0, 109.5, 108.5, 107.1, 105.6, 102.6, 98.0, 92.0, 84.0, 81.0, 75.9, 68.8, 61.9, 56.6, 53.5, 46.1, 45.9, 40.9, 40.7, 39.1, 12.1. HRMS (nanochip-ESI/LTQ-Orbitrap): *m*/*z*: calcd for C_53_H_48_N_7_O_9_^+^ ([M + H]^+^), 926.3508; found, 926.3539. ^1^H NMR, ^13^C NMR spectra, IR data (Supporting Information S27).

##### Preparation of **LNO-CM-ELA** NPs

To a suspension of **LNO-N**_**3**_ (2 mg) in EtOH (1 mL) were added DMF (1 mL) and **CM-ELA** (10 mM in DMSO, 50 μL, 0.4 μmol) in DMSO. The suspension was ultra-sonicated for 16 h under an argon atmosphere and dark conditions (40–60 °C). The suspension was centrifuged (10 min, 4 700 rpm), and the supernatant was discarded for quantification of unreacted **CM-ELA** (Supporting Information S32). The solid residue was sequentially washed and centrifuged (HERAEUS Biofuge 13 centrifugator) with DMF (1 mL) and EtOH (1 mL, three times). The combined supernatant phases were evaporated under reduced pressure, dissolved in DMSO (50 μL), and diluted with EtOH (1 mL) for quantification. The resulting **LNO-CM-ELA** NPs were suspended in EtOH (1 mL). An aliquot (20 μL) was withdrawn and diluted in PBS 0.1× (1 mL). After ultrasonication for 30 min, the sample was analyzed in a Malvern NanoZ system to measure the mean hydrodynamic diameter and zeta potential. Scanning TEM (STEM) with EDX analysis was performed at the Interdisciplinary Centre for Electron Microscopy (CIME, EPFL, Lausanne, Switzerland) on a FEI Titan Themis 60-300 microscope, and the samples were deposited on silica-free copper-carbon grids.

## Supporting Information Available

Synthesis protocols for compounds **1**, **5**, **7**, and **DIBO-ELA**; TEM and XRD characterization of LNO NPs; preparation and characterization of **LNO-N**_**3**_ NPs; analytical data (^1^H and ^13^C NMR spectra and IR data) for compounds **2**, **3**, **4**, **6**, **ELA**, and **DIBO-CM-ELA**; estimation of the **ELA** loading rate; EGFR expression in DU145 cells; quantitative analysis by UHPLC–ESI–HRMS; characterization of the release of **ELA** from **LNO-CM-ELA** NPs; irradiation intensity distribution in cell plates; temperature increase upon NIR irradiation; and DLA characterization of LNO, **LNO-N**_**3**_, and **LNO-CM-ELA** NPs (PDF)

## Abbreviations

APTES: (3-aminopropyl)triethoxysilane
ATP: adenosine triphosphate
ATR: attenuated total reflectance
BFO: bismuth ferrite
CM: coumarin
DCM: dichloromethane
DIBO: dibenzocyclooctyne
DLS: dynamic light scattering
DMAP: 4-dimethylaminopyridine
DMEM: Dulbecco’s modified Eagle’s medium
DMF: *N*,*N*-dimethylformamide
DMF-DMA: dimethylformamide–dimethylacetal
DMSO: dimethyl sulfoxide
EDCI: 1-ethyl-3-(3-dimethylaminopropyl)carbodiimide
EDX: energy-dispersive X-ray
EGFR: epidermal growth factor receptor
**ELA**: erlotinib-amine derivative
ESI: electrospray ionization
FCC: flash column chromatography
FBS: fetal bovine serum
FCS: fetal calf serum
FROG: frequency-resolved time gating
FTIR: Fourier transform infrared
HNP: harmonic nanoparticle
HOBt: hydroxybenzotriazole
HRMS: high-resolution mass spectrometry
IR: infrared
LNO: lithium niobate
MTT: 3-(4,5-dimethyl-2-thiazoyl)-2,5-diphenyltetrazolium bromide
MS: mass spectrometry
NIR: near infrared
NMR: nuclear mass resonance
NP: nanoparticle
NSCLC: non-small-cell lung cancer
PBS: phosphate-buffered saline
PDI: polydispersity index
PE: petroleum ether
QTOF: quadrupole time-of-flight
RNA: ribonucleic acid
rt: room temperature
SD: standard deviation
SHG: second-harmonic generation
STEM: scanning transmission electron microscopy
SWIR: shortwave infrared
THG: third-harmonic generation
TK: tyrosine kinase
TLC: thin-layer chromatography
UCNP: upconversion nanoparticle
UHPLC: ultrahigh-performance liquid chromatography
UV: ultraviolet
ZP: zeta potential
