# Supplementary material for: Photoresponsive Nanocarriers Based on Lithium Niobate Nanoparticles for Harmonic Imaging and On-Demand Release of Anticancer Chemotherapeutics
Source: ACS Nanosci Au. 2022 Jun 3;2(4):355–66. doi: 10.1021/acsnanoscienceau.1c00044 (PMC9389616; doi:10.1021/acsnanoscienceau.1c00044)

Supporting information

Photoresponsive Nanocarriers based on Lithium Niobate Nanoparticles for Harmonic Imaging and On-Demand Release of Anticancer Chemotherapeutics

Adrian Gheata,[1] Geoffrey Gaulier,[2] Gabriel Campargue,[2] Jérémy Vuilleumier,[1] Simon Kaiser,[3] Ivan Gautschi,[3] Florian Riporto,[4] Sandrine Beauquis,[4] Davide Staedler,[3] Dario Diviani,[3] Luigi Bonacina,[2] Sandrine Gerber-Lemaire[1]*

[1]Institute of Chemical Sciences and Engineering, Ecole Polytechnique Fédérale de Lausanne, Group for Functionalized Biomaterials, EPFL SB ISIC SCI-SB-SG, Station 6, CH-1015 Lausanne, Switzerland
[2]Department of Applied Physics, Université de Genève, 22 Chemin de Pinchat, CH-1211 Genève, Switzerland
[3]Department of Biomedical Sciences, Université de Lausanne, 7 Rue du Bugnon, CH-1005 Lausanne, Switzerland
[4]Université Savoie Mont-Blanc, SYMME, F-74000 Annecy, France.

*E-mail: sandrine.gerber@epfl.ch

Table of contents

Details on experimental protocols and characterization instrumentation S-3
Preparation of 2-(4-cyano-2-methoxyphenoxy)ethyl acetate (**1**) S-3
Preparation of ethyl *N*-ethyl-*N*-(4-((((4-nitrophenoxy)carbonyl)oxy)methyl)-2-oxo-2*H*-chromen-7-yl) glycinate (**5**) S-9
Preparation of compound **7** S-15
Preparation of compound **2**: ^{1}H-NMR, ^{13}C NMR and IR data S-17
Preparation of compound **3**: ^{1}H-NMR, ^{13}C NMR and IR data S-18
Preparation of compound **4**: ^{1}H-NMR, ^{13}C NMR and IR data S-19
Preparation of **ELA**: ^{1}H-NMR, ^{13}C NMR and IR data S-23
Preparation of compound **6**: ^{1}H-NMR, ^{13}C NMR and IR data S-25
Preparation of **CM-ELA**: ^{1}H-NMR, ^{13}C NMR and IR data S-27
Preparation of **DIBO-ELA**: ^{1}H-NMR, ^{13}C NMR and IR data S-28
TEM and XRD characterization of LNO HNPs S-31
Preparation of **LNO-N$_3$** NPs S-31
Determination of **ELA** loading at the surface of **LNO-CM-ELA** NPs S-33
EGFR expression in DU145 cells S-34
Characterization of UV light- and NIR excitation-triggered release of **ELA** from **LNO-CM-ELA** NPs S-35
Quantitative analysis by UHPLC-ESI-HRMS S-38
Irradiation intensity distribution in cell plates S-40
Detection of ELA release upon irradiation on a tunable MHz laser (setup 2) S-41
DLS characterization of LNO NPs, **LNO-N$_3$** NPs and **LNO-CM-ELA** NPs S-42
Emission of aggregated **LNO-CM-ELA** NPs S-43

Details on experimental protocols and characterization instrumentation

Reagent-grade solvents (Fluka, Riedel-de-Haën) and chemicals (Aldrich, Acros, Fluka, Sigma, Maybridge, TCI Chemicals, Apollo, abcr and Fluorochem) were used without further purification. All reactions were performed in flame-dried glassware under an inert atmosphere of nitrogen. All products were dried under vacuum (10-2 bar) before analytical characterization. Reactions were monitored by thin layer chromatography (TLC) on pre-coated aluminum plates SiO2 60 F254 (Merck, Darmstadt, Germany). The compounds were visualized by 254 nm light or stained with solutions of KMnO4, Pancaldi reagent [(NH4)6MoO4, Ce(SO4)2, H2SO4, H2O], ninhydrin or iodine vapors. Purifications were performed by flash chromatography (FC) on silica gel (Merck N° 9385 silica gel 60, 230-400 mesh, particle size 40-63 µm). NMR spectra were recorded on Bruker Avance III-400, Bruker Avance-400 or Bruker DRX-400 spectrometers (Bruker, Billerica, MA, USA) at rt. 1H frequency is at 400.13 MHz, 13C frequency is at 100.62 MHz. Chemical shifts are reported downfield from tetramethylsilane. 1H signals are reported in ppm with the internal chloroform signal at 7.26 ppm, the internal methanol signal at 3.31 ppm or the internal DMSO signal at 2.50 ppm as references. 13C-NMR signals are reported in ppm with the internal chloroform signal at 77.00 ppm, the internal methanol signal at 49.00 ppm or the internal DMSO signal at 39.5 as internal references. The resonance multiplicity is described as s (singlet), d (doublet), t (triplet), q (quartet), quin (quintet), m (multiplet). Broad signals are indicated as br. Coupling constants (J) are given in hertz (Hz). Ultrasonication was performed on Elmasonic P 120 H or Branson 1800 Ultrasonic Cleaner sonicators. IR spectra were recorded on a Jasco FT/IR-4100 spectrometer outfitted with a PIKE technology MIRacleTM ATR accessory as neat films compressed onto a Zinc Selenide window. The spectra are reported in cm-1. The qualitative accurate masses were measured by ESI-TOF using the Xevo G2-S QTOF (Waters) and nanoESI-FT-MS using the Elite™ Hybrid Ion Trap-Orbitrap (ThermoFisher) Mass Spectrometer. Quantitative MS analyses were performed on the 6530 Accurate-Mass Q-TOF LC/MS mass spectrometer coupled to the 1290 Infinity UHPLC system (Agilent Technologies, USA). The separation was achieved using an ACQUITY UHPLC® BEH C18 1.7µm column, 2.1 mm x 50 mm (Waters) heated at 30°C using water and acetonitrile as mobile phases.

Preparation of 2-(4-Cyano-2-methoxyphenoxy)ethyl acetate (1)

Adapted from protocols described in:

Thompson, R.; Doggrell, S.; Hoberg, J. O. Potassium Channel Activators Based on the Benzopyran Substructure: Synthesis and Activity of the C-8 Substituent. *Bioorg. Med. Chem.* **2003**, *11*, 1663–1668.

Gurjar, J.; Bater, J.; Fokin, V. V. Sulfuryl Fluoride Mediated Conversion of Aldehydes to Nitriles. *Chem. Eur. J.* **2019**, *25*, 1906–1909.

Passemard, S. Functionalization of Nanoparticles for Targeted Cancer Imaging and Diagnosis, EPFL TH N°6248.

Scheme S1. Synthetic route toward 2-(4-Cyano-2-methoxyphenoxy)ethyl acetate (**1**)

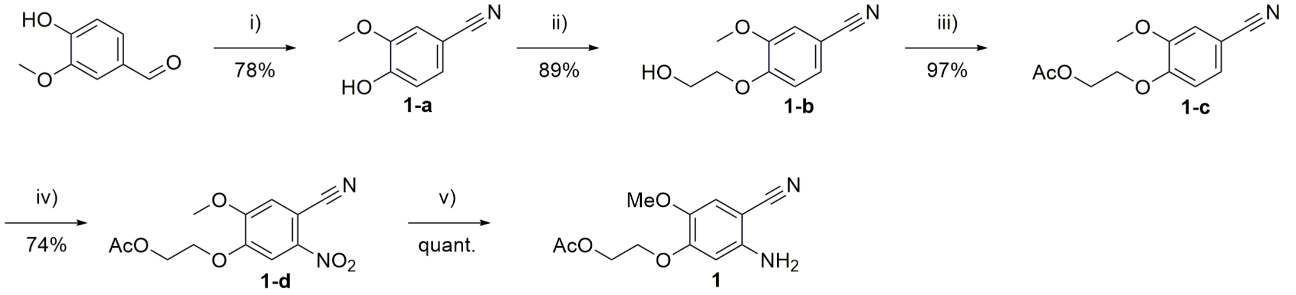

Reagents and conditions: i) $NH_2OH\cdot HCl$, AcOH, reflux, 90 min; ii) 2-chloroethanol, K_2CO_3, DMF,150°C, 8 h; iii) Acetyl chloride, pyridine, 0°C, 2 h; iv) HNO_3, 0°C to r.t., 35 min; v) $Pd(OAc)_2$, PMHS, KF, THF, r.t., 30 min.

4-Hydroxy-3-methoxybenzonitrile (**1-a**)

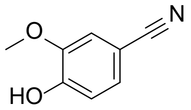

To solution of vanillin (10.0 g, 65.7 mmol, 1.0 eq) in AcOH (80 mL) was added hydroxylamine hydrochloride (6.9 g, 98.6 mmol, 1.5 eq). The reaction mixture was refluxed for 1.5 h. The mixture was cooled to rt, diluted with Et_2O (100 mL). The organic layer was washed with H_2O (2 X 100 mL), brine (1 X 100 mL), dried over $MgSO_4$, filtered and concentrated under reduced pressure. The crude product was purified by FCC (PE / EtOAc, 1:1) to afford **1-a** as a pale yellow solid (7.7 g, 51.4 mmol, 78%). The analytical data were in accordance with previously reported data.

^{1}H NMR (400 MHz, Chloroform-*d*): δ 7.23 (dd, *J* = 8.3, 1.8 Hz, 1H, Ar-*H*), 7.08 (d, *J* = 1.8 Hz, 1H, Ar-*H*), 6.96 (d, *J* = 8.2 Hz, 1H, Ar-*H*), 6.08 (s, 1H, O*H*), 3.93 (s, 3H, C*H*$_3$) ppm.

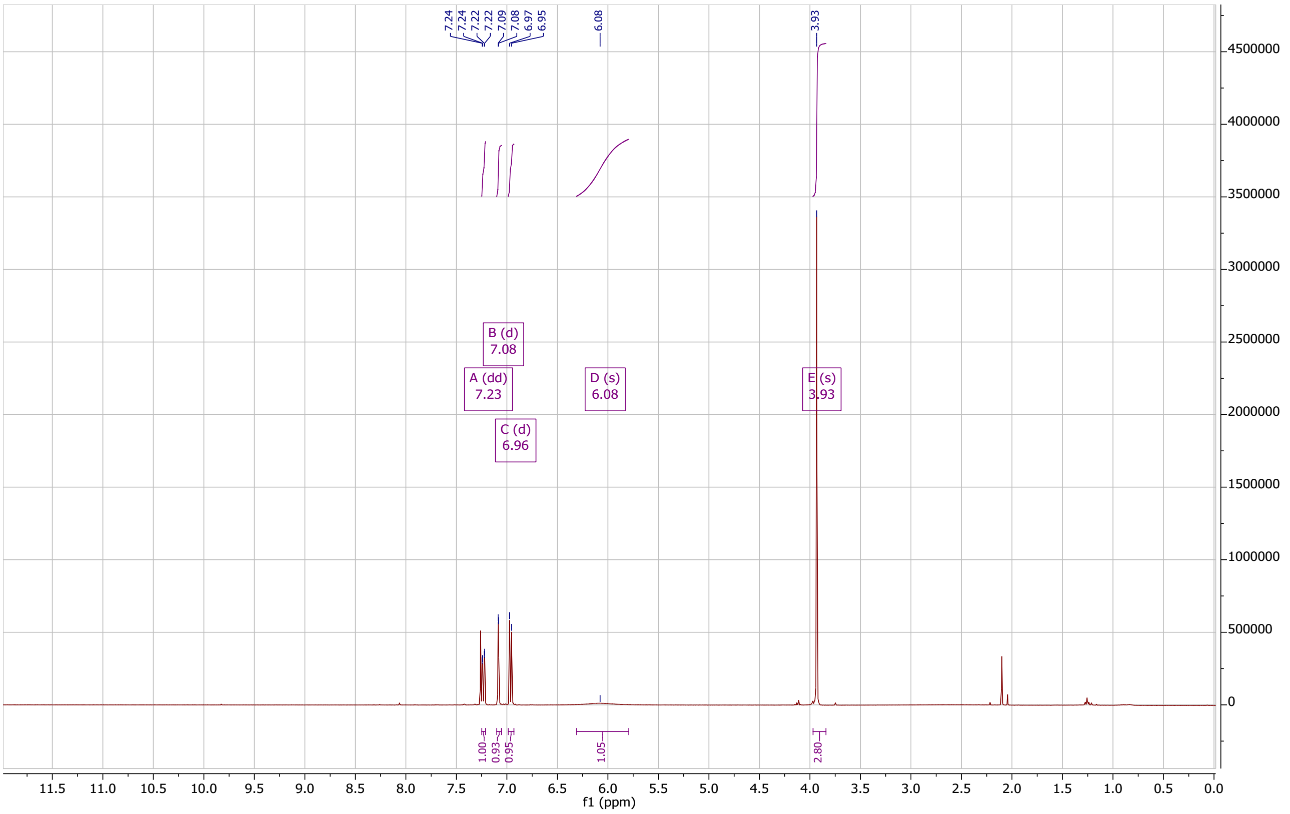

4-(2-Hydroxyethoxy)-3-methoxybenzonitrile (**1-b**)

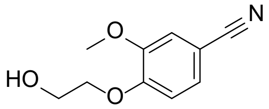

Compound **1-a** (7.7 g, 51.4 mmol, 1.0 eq), 2-chloroethanol (3.8 mL, 56.6 mmol, 1.1 eq) and K_2CO_3 (21.3 g, 154.2 mmol, 3.0 eq) were dissolved in DMF (100 mL). The solution was stirred at 150°C for 8 h. The mixture was cooled to rt, diluted with sat. aqueous NH_4Cl (300 mL) and extracted with DCM (3 X 300 mL). The combined organic layers were dried over $MgSO_4$, filtered and concentrated under reduced pressure. The crude product was purified by FCC (PE / EtOAc, 1:1) to afford **1-b** as a white solid (8.9 g, 45.9 mmol, 89%). The analytical data were in accordance with previously reported data.

^{1}H NMR (400 MHz, Chloroform-*d*): δ 7.28 (d, *J* = 1.8 Hz, 1H, Ar-*H*), 7.10 (d, *J* = 1.9 Hz, 1H, Ar-*H*), 6.93 (d, *J* = 8.3 Hz, 1H, Ar-*H*), 4.20 – 4.12 (m, 2H, C*H*$_2$-OAr), 4.07 – 3.99 (m, 2H, C*H*$_2$-OH), 3.89 (s, 3H, C*H*$_3$), 2.25 (t, *J* = 6.3 Hz, 1H, O*H*) ppm.

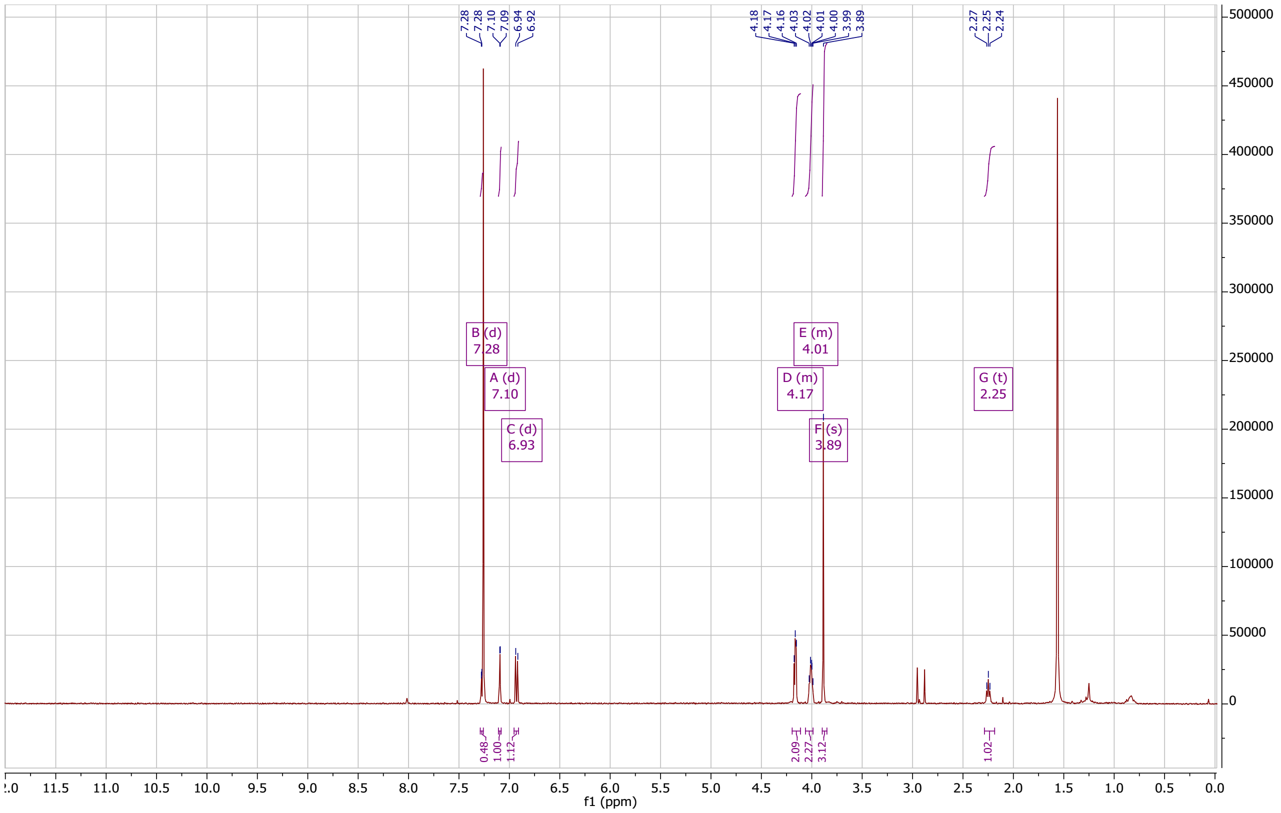

2-(4-Cyano-2-methoxyphenoxy)ethyl acetate (**1-c**)

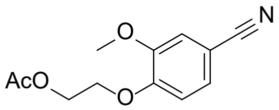

Acetyl chloride (4.9 mL, 68.9 mmol, 1.5 eq) and pyridine (5.6 mL, 68.9 mmol, 1.5 eq) were added dropwise to a solution of **1-b** (8.9 g, 45.9 mmol, 1.0 eq) in THF (70 mL) at 0°C. The reaction mixture was warmed to rt and stirred for 2 h. The mixture was diluted with DCM (300 mL) and washed with sat. aqueous copper sulfate solution (2 X 300 mL) and H_2O (1 X 300 mL). The organic layer was dried over $MgSO_4$, filtered and concentrated under reduced pressure to afford **1-c** as a white solid (10.5 g, 44.5 mmol, 97%). The analytical data were in accordance with previously reported data.

^{1}H NMR (400 MHz, Chloroform-*d*): δ 7.26 (s, 1H, Ar-*H*), 7.10 (d, *J* = 1.9 Hz, 1H, Ar-*H*), 6.92 (d, *J* = 8.3 Hz, 1H, Ar-*H*), 4.51 – 4.39 (m, 2H, C*H*$_2$-OAc), 4.27 (t, *J* = 4.9 Hz, 2H, C*H*$_2$-OAr), 3.89 (s, 3H, C*H*$_3$-OAr), 2.10 (s, 3H, C*H*$_{3\,Ac}$) ppm.

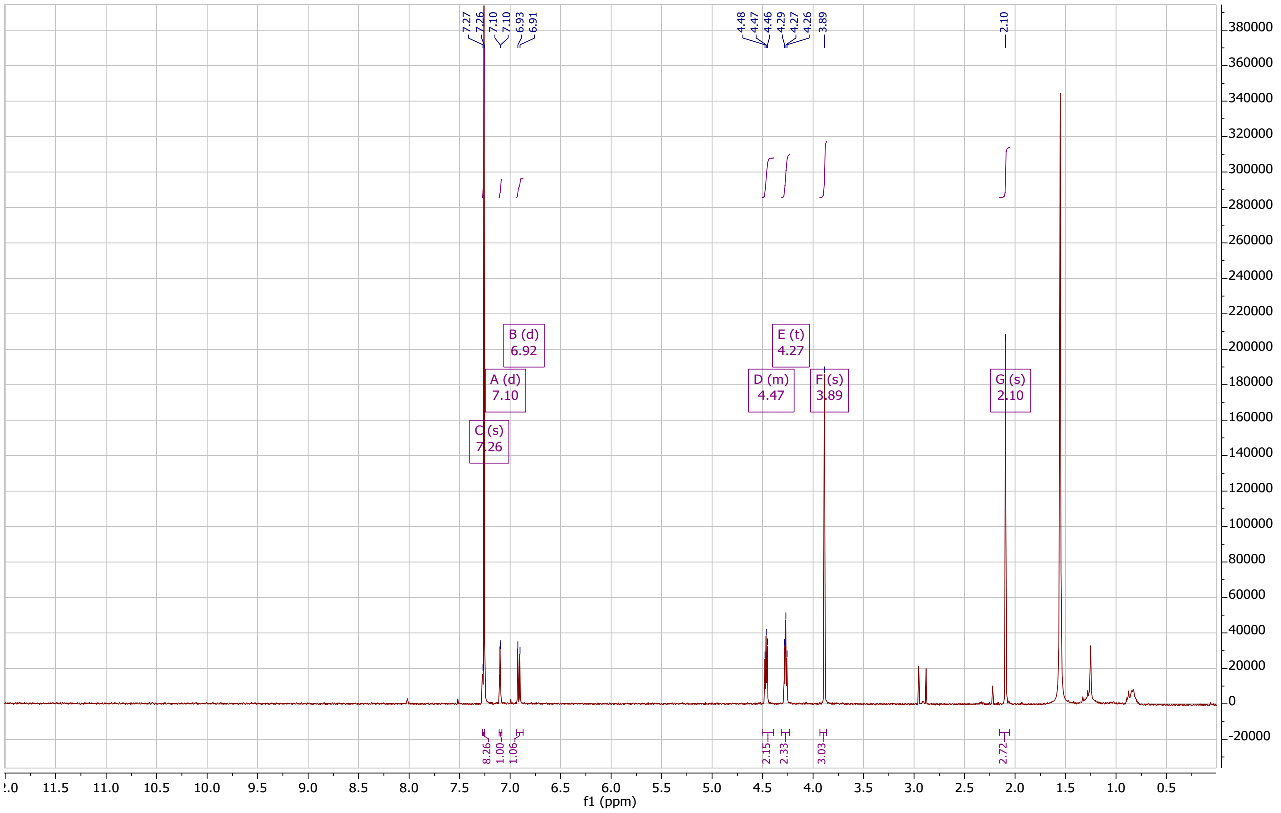

2-(4-Cyano-2-methoxy-5-nitrophenoxy)ethyl acetate (**1-d**)

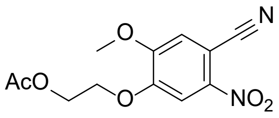

Compound **1-c** (0.5 g, 2.1 mmol, 1.0 eq) was added portionwise to fuming HNO_3 (0.7 mL) at 0°C. The solution was warmed to rt and stirred for 35 min. The mixture was poured into a mixture of ice / H_2O. The precipitate was filtered and recrystallized from EtOAc to afford **1-d** as a white solid (0.4 g, 1.6 mmol, 74%). The analytical data were in accordance with previously reported data.

^{1}H NMR (400 MHz, Chloroform-*d*): δ 7.83 (s, 1H, Ar-*H*), 7.22 (s, 1H, Ar-*H*), 5.13 (s, 2H), 4.50 (dd, *J* = 5.5, 3.8 Hz, 2H, CH_2-OAc), 4.40 – 4.28 (m, 2H, CH_2-OAr), 4.01 (s, 3H, CH_3-OAr), 2.10 (s, 3H, C$H_{3\,Ac}$) ppm.

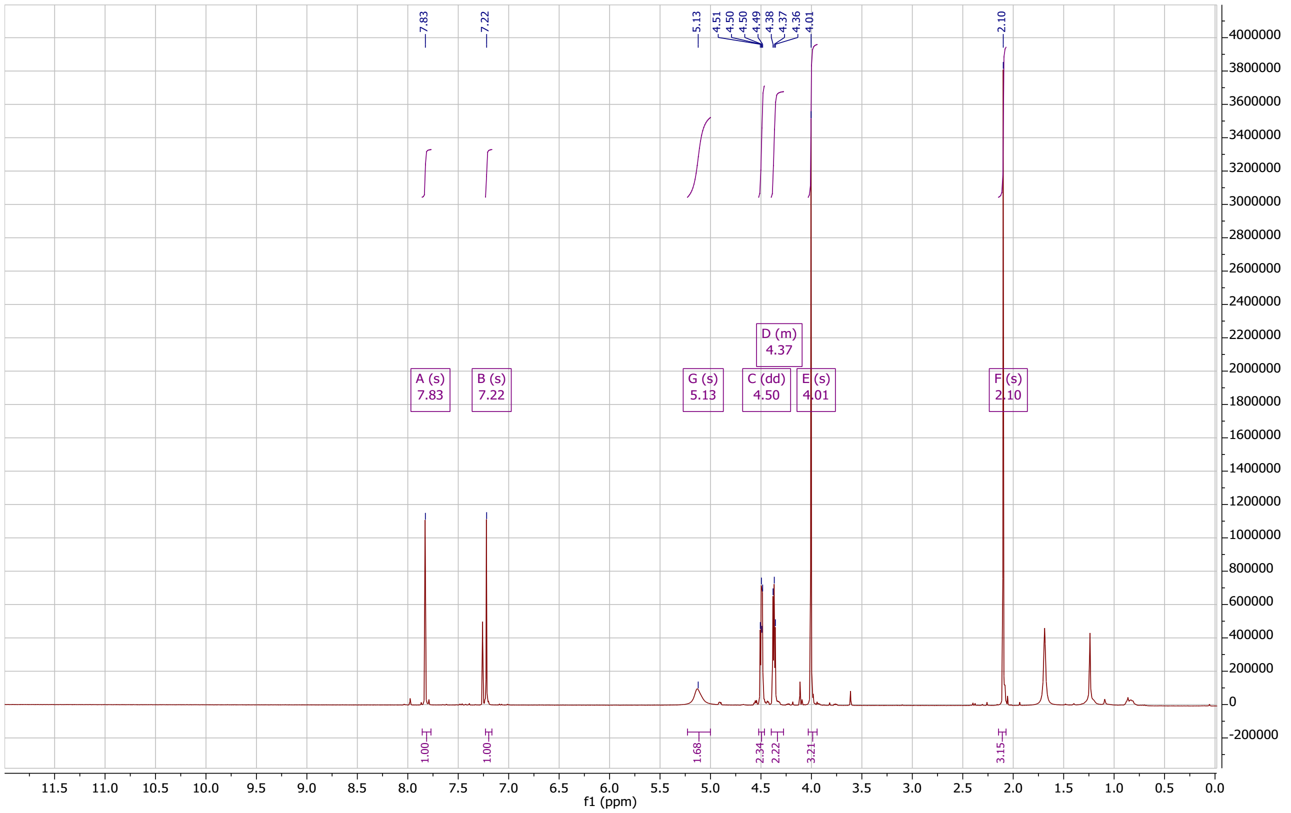

2-(5-Amino-4-cyano-2-methoxyphenoxy)ethyl acetate (**1**)

$Pd(OAc)_2$ (0.23 g, 1.0 mmol, 5 mol%) was added to a solution of **1-d** (5.61 g, 40.0 mmol, 1.0 eq) in dry and degassed THF (100 mL). A 1M KF solution in H_2O (40 mL, 80.0 mmol, 2.0 eq) and PHMS (4.8 mL, 80.0 mmol, 4.0 eq) were slowly added and the solution was stirred at rt for 30 min. The solution was diluted with Et_2O (200 mL) and H_2O (160 mL). The aqueous layer was extracted with Et_2O (3 X 200 mL). The combined organic layers were dried over $MgSO_4$, filtered and concentrated under reduced pressure. The crude product was purified by FCC (EtOAc) to afford **1** as a brown solid (10.02 g, 40.0 mmol, quant.). The analytical data were in accordance with previously reported data.

^{1}H NMR (400 MHz, Chloroform-*d*): δ 6.82 (s, 1H, Ar-*H*), 6.27 (s, 1H, Ar-*H*), 4.45 (t, *J* = 4.9 Hz, 3H, CH_2-OAc), 4.20 (t, *J* = 4.9 Hz, 3H, CH_2-OAr), 3.79 (s, 3H, CH_3-OAr), 2.10 (s, 3H, C$H_{3\,Ac}$) ppm.

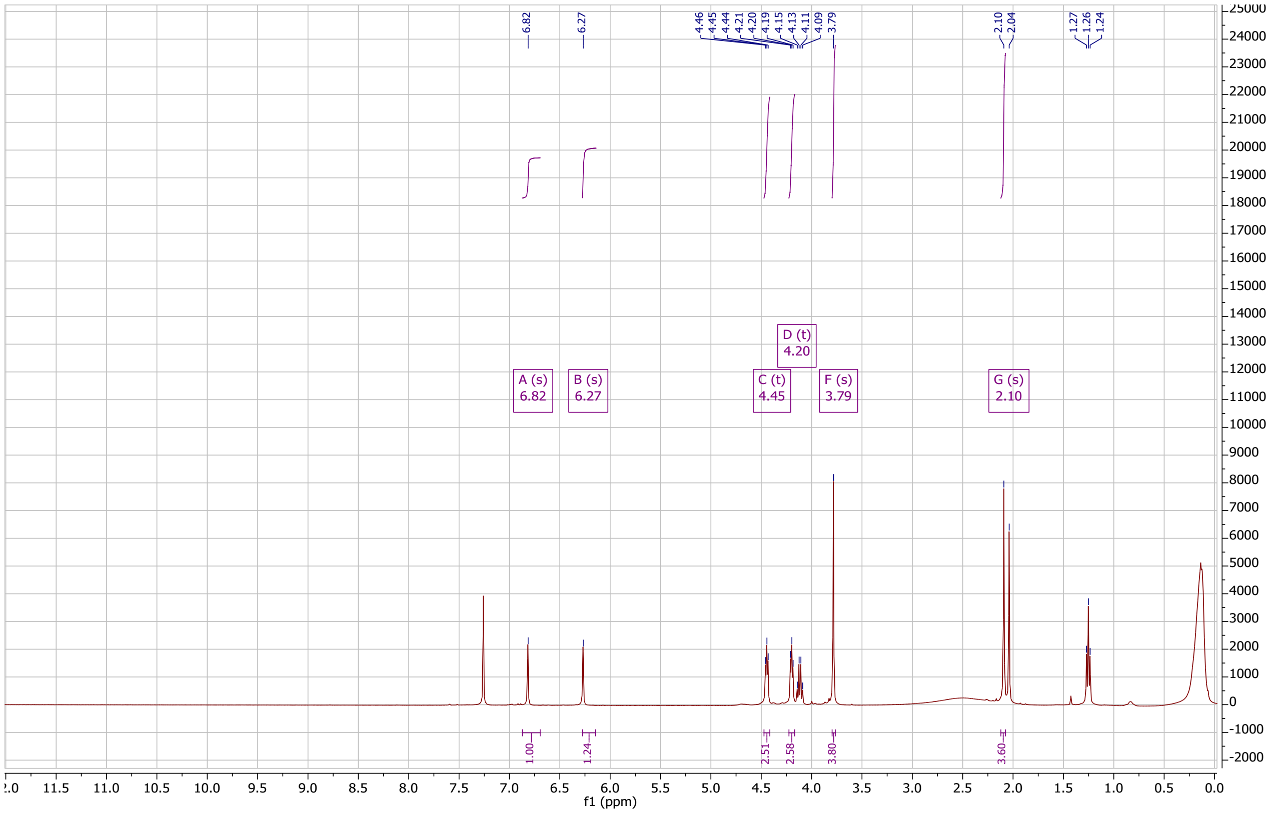

6.82
6.27
4.46
4.45
4.44
4.21
4.20
4.19
4.15
4.13
4.11
4.09
3.79
2.10
2.04
1.27
1.26
1.24
A (s)
6.82
B (s)
6.27
C (t)
4.45
D (t)
4.20
F (s)
3.79
G (s)
2.10
1.00
1.24
2.51
2.58
3.80
3.60
f1 (ppm)

__**Preparation of ethyl *N*-ethyl-*N*-(4-((((4-nitrophenoxy)carbonyl)oxy)methyl)-2-oxo-2*H*-chromen-7-yl)glycinate (5)**__

Scheme S2. Synthetic route toward ethyl N-ethyl-N-(4-((((4-nitrophenoxy)carbonyl)oxy)methyl)-2-oxo-2H-chromen-7-yl)glycinate (**5**)

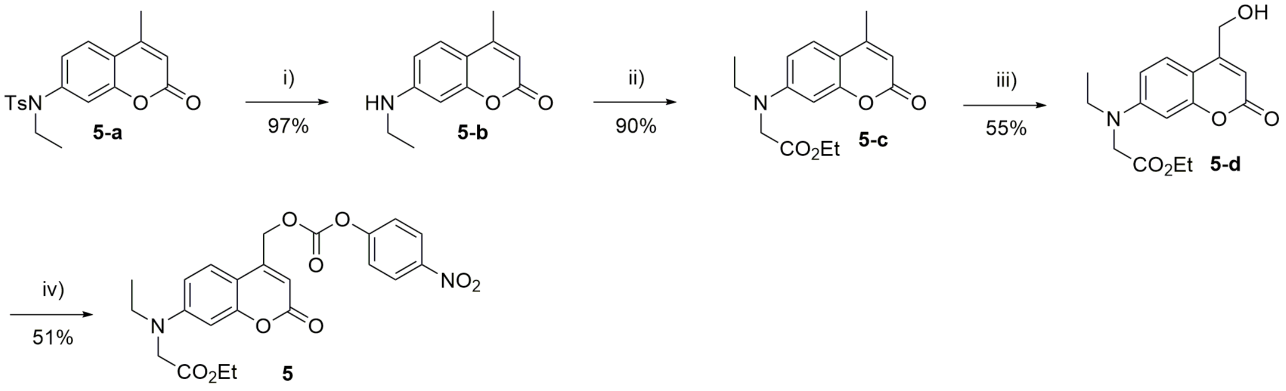

Reagents and conditions: i) H_2SO_4, 0°C, 1 h; ii) ethyl bromoacetate, K_2CO_3, TBAB, NaI, ACN, reflux, 46 h; iii) SeO_2; *p*-xylene, reflux 24 h; then $NaBH_4$, MeOH, rt, 3 h; iv) 4-nitrophenyl chloroformate, DIPEA, DCM, r.t., 16 h.

__7-(Ethylamino)-4-methyl-2*H*-chromen-2-one (**5-b**)__

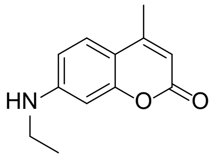

4-Methyl-*N*-(4-methyl-2-oxo-2*H*-chromen-7-yl)benzenesulfonamide (**5-a**) (1.1 g, 3.1 mmol, 1.0 eq) was added to conc. sulfuric acid (5 mL) and the reaction mixture was stirred at 0°C for 1 h. The solution was poured into H_2O (20 mL) and neutralized with sat. aqueous $NaHCO_3$ (150 mL).The aqueous layer was extracted with DCM (3 X 200 mL). The combined organic layers were dried over $MgSO_4$, filtered and concentrated under reduced pressure. The crude product was purified by FCC (EtOAc / PE, 6:4) to afford **5-b** as a yellow solid (0.6 g, 2.9 mmol, 97%).

The analytical data were in accordance with the data reported in:
Lin, Q.; Huang, Q.; Li, C.; Bao, C.; Liu, Z.; Li, F.; Zhu, L. Anticancer Drug Release from a Mesoporous Silica Based Nanophotocage Regulated by Either a One- or Two-Photon Process. *J. Am. Chem. Soc.* **2010**, *132*, 10645–10647.

^{1}H NMR (400 MHz, Chloroform-*d*): δ 7.35 (d, *J* = 8.6 Hz, 1H, CM-*H*), 6.49 (dd, *J* = 8.6, 2.4 Hz, 1H, CM-*H*), 6.44 (d, *J* = 2.3 Hz, 1H, CM-*H*), 5.98 (d, *J* = 1.5 Hz, 1H, CM-*H*), 4.11 (s, 1H, N-*H*), 3.22 (qd, *J* = 7.1, 5.2 Hz, 2H, $CH_{2\,ester}$), 2.34 (d, *J* = 1.2 Hz, 3H, $CH_{3\,CM}$), 1.29 (t, *J* = 7.2 Hz, 3H, $CH_{3\,ester}$) ppm.

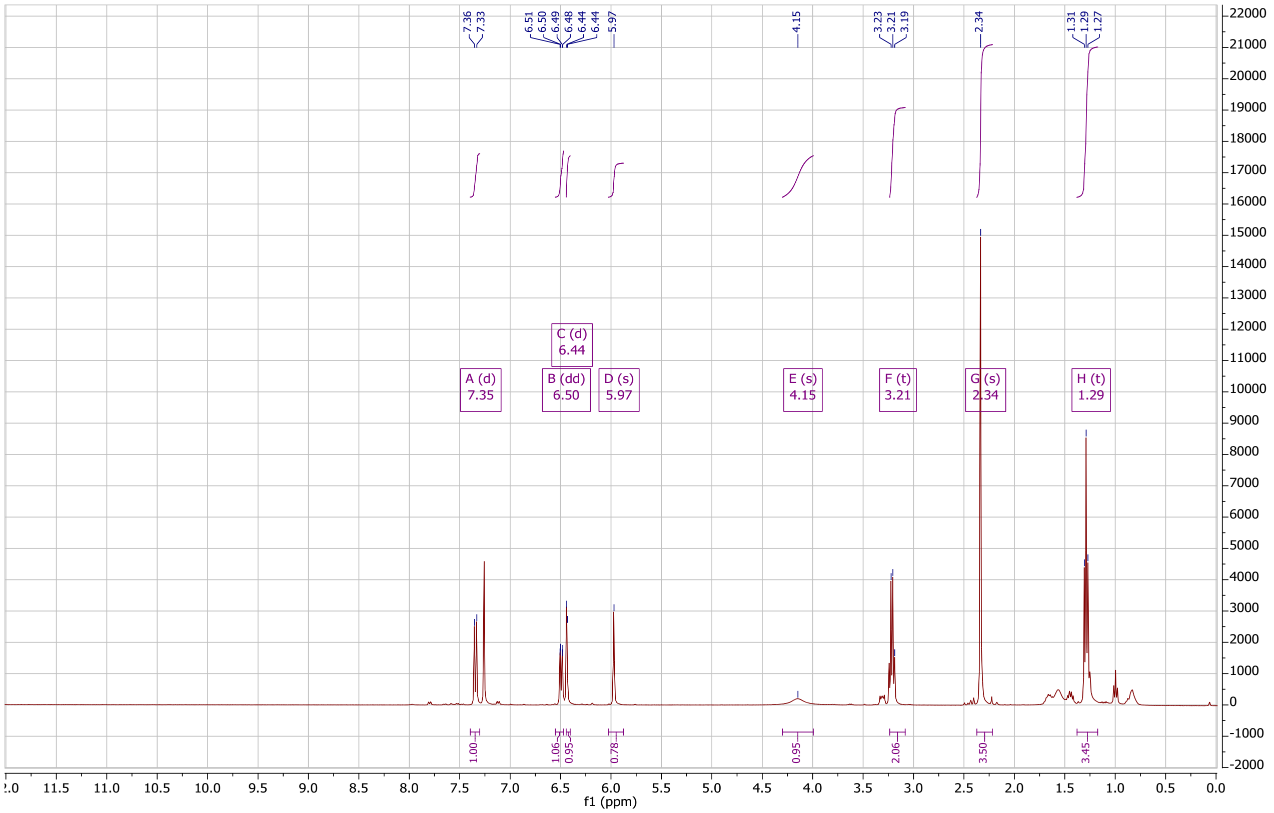

Ethyl *N*-ethyl-*N*-(4-methyl-2-oxo-2*H*-chromen-7-yl)glycinate (**5-c**)

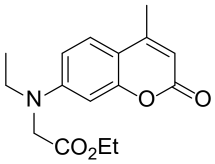

Compound **5-b** (1.0 g, 4.9 mmol, 1.0 eq), K_2CO_3 (2.0 g, 14.7 mmol, 3.0 eq), NaI (0.15 g, 0.98 mmol, 0.2 eq) and tetrabutylammonium bromide (0.32 g, 0.92 mmol, 0.2 eq) were dissolved in acetonitrile (50 mL). Ethyl bromoacetate (5.5 mL, 49.0 mmol, 10.0 eq) was added and the mixture was refluxed for 48 h. The reaction mixture was cooled to rt, filtered and the solvent was removed under reduced pressure. The residue was dissolved in EtOAc (100 mL) and washed with H_2O (3 X 100 mL). The organic layer was dried over $MgSO_4$, filtered and concentrated under reduced pressure. The residue was purified by FCC (EtOAc / PE, 6:4) to afford **5-c** as a yellow solid (1.29 g, 4.5 mmol, 90%).

IR (neat, cm^{-1}): 2965, 1725, 1620, 1535, 1410, 1194, 1080, 770, 715.

HRMS (nanochip-ESI/LTQ-Orbitrap) m/z: $[M + H]^+$ Calcd for $C_{16}H_{20}NO_4^+$ 290.1387; Found 290.1385.

^{1}H NMR (400 MHz, Chloroform-*d*): δ 7.40 (d, *J* = 8.9 Hz, 1H, CM-*H*), 6.56 (dd, *J* = 8.9, 2.6 Hz, 1H, CM-*H*), 6.48 (d, *J* = 2.6 Hz, 1H, CM-*H*), 5.99 (d, *J* = 1.5 Hz, 1H, CM-*H*), 4.22 (q, *J* = 7.1 Hz, 2H, $CH_{2\ ester}$), 4.07 (s, 2H, N-CH_2-CO_2Et), 3.52 (q, *J* = 7.1 Hz, 2H, N-CH_2-CH_3), 2.34 (d, *J* = 1.1 Hz, 3H, $CH_{3\ CM}$), 1.27 (dt, *J* = 12.5, 7.2 Hz, 6H, N-CH_2-CH_3 and $CH_{3\ ester}$) ppm.

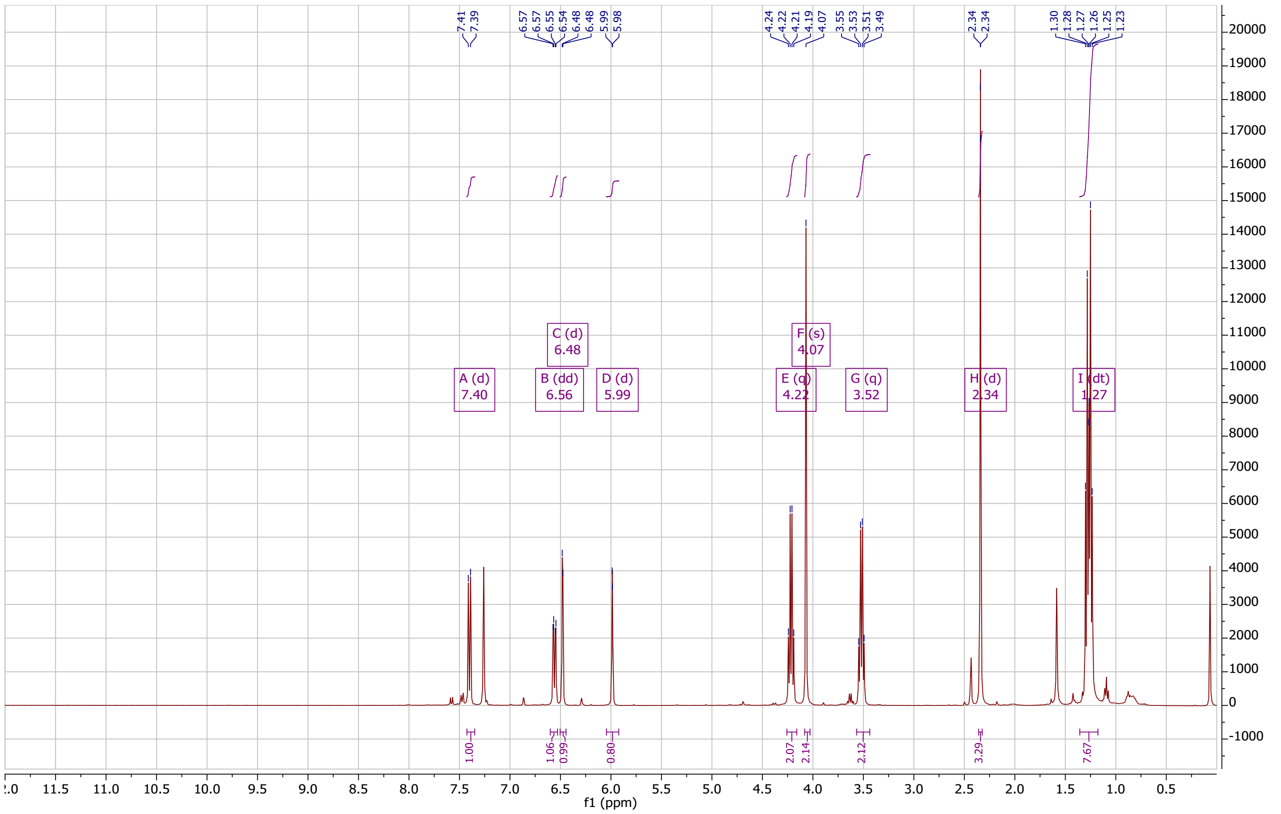

^{13}C NMR (101 MHz, Chloroform-*d*): δ 170.2 (*C*O), 162.1 (C_{CM}), 155.9 (C_{CM}), 152.9 (C_{CM}), 150.9 (C_{CM}), 125.7 (CH_{CM}), 110.5 (C_{CM}), 109.9 (CH_{CM}), 108.8 (CH_{CM}), 98.7 (CH_{CM}), 61.6 (CH_2), 52.3 (CH_2), 46.8 (CH_2), 18.6 (CH_3), 14.4 (CH_3), 12.4 (CH_3) ppm.

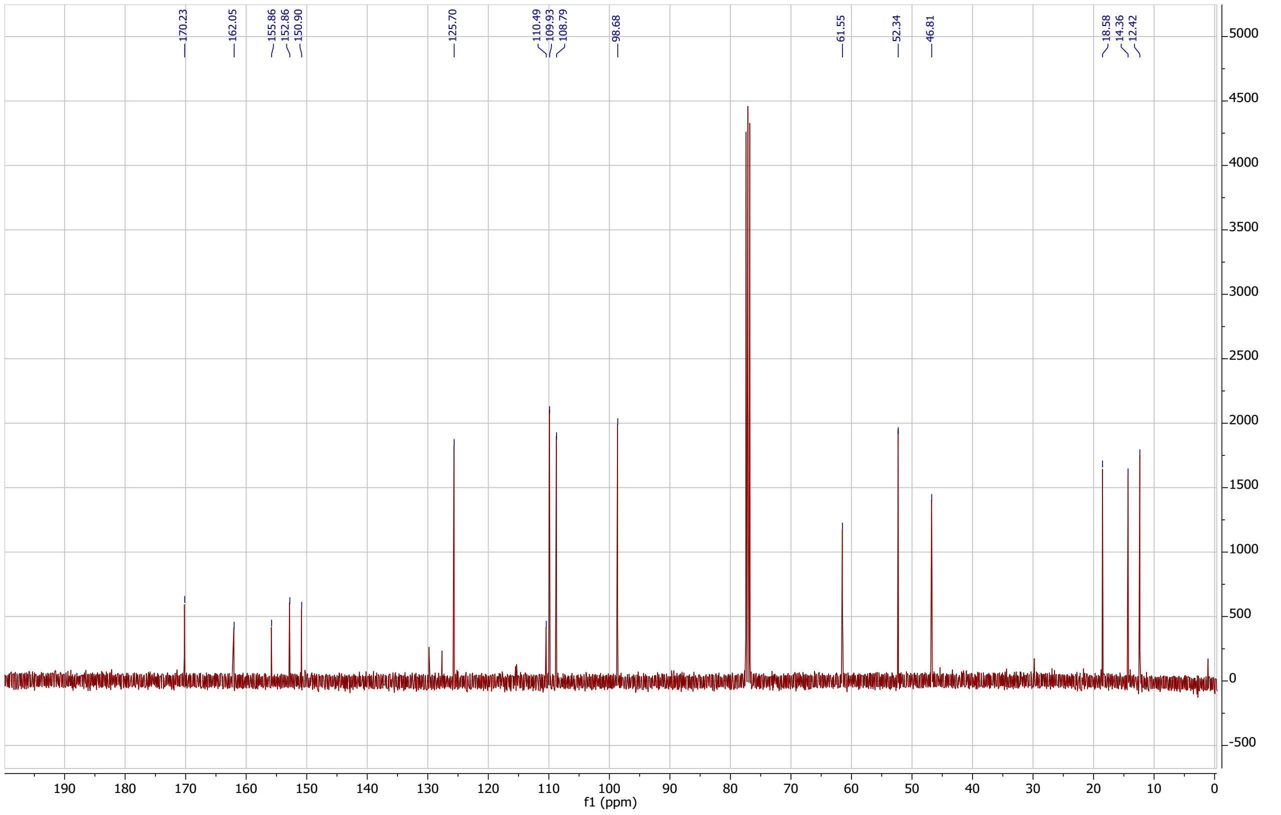

Ethyl *N*-ethyl-*N*-(4-(hydroxymethyl)-2-oxo-2*H*-chromen-7-yl)glycinate (**5-d**)

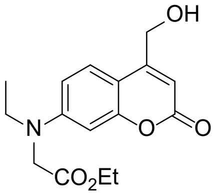

Compound **5-c** (1.29 g, 4.46 mmol, 1.0 eq) and SeO_2 (0.99 g, 8.92, 2.0 eq) were dissolved in *p*-xylene (40 mL). The reaction mixture was refluxed, under vigorous stirring, under argon atmosphere for 24 h. The reaction mixture was cooled to rt, filtered through paper filter and concentrated under reduced pressure. The crude mixture was dissolved in methanol (30 mL) and $NaBH_4$ (0.34 g, 8.92 mmol, 2.0 eq) was added. The solution was stirred at rt for 3 h and the reaction mixture was neutralized with 1M HCl (10 mL) and H_2O (100 mL). The solution was extracted with DCM (3 X 150 mL) and the combined organic layers were dried over $MgSO_4$, filtered and concentrated under reduced pressure. The crude mixture was purified by FCC (EtOAc / PE, 3:7 to 6:4) to afford **5-d** as a yellow solid (0.75 g, 2.46 mmol, 55% yield).

IR (neat, cm^{-1}): 1715, 1610 , 1515, 1440, 1405, 1190, 1095, 810, 665.

HRMS (nanochip-ESI/LTQ-Orbitrap) m/z: $[M + H]^+$ Calcd for $C_{16}H_{20}NO_5^+$ 306.1336; Found 306.1332.

^{1}H NMR (400 MHz, Chloroform-*d*): δ 7.32 (d, *J* = 8.9 Hz, 1H, CM-*H*), 6.53 (dd, *J* = 8.9, 2.6 Hz, 1H, CM-*H*), 6.49 (d, *J* = 2.6 Hz, 1H, CM-*H*), 6.30 (s, 1H, CM-*H*), 4.81 (d, *J* = 6.1 Hz, 2H, C$H_{2\ CM}$), 4.22 (q, J = 7.1Hz, 2H, C$H_{2\ ester}$), 4.07 (s, 2H, N-CH_2-CO_2Et), 3.51 (q, *J* = 7.1 Hz, 2H, NH-CH_2-CH_3), 2.07 (t, *J* = 6.1 Hz, 1H, RO-*H*), 1.28 (t, *J* = 7.1 Hz, 3H, C$H_{3\ ester}$), 1.25 (t, *J* = 7.1 Hz 3H, N-CH_2-CH_3) ppm.

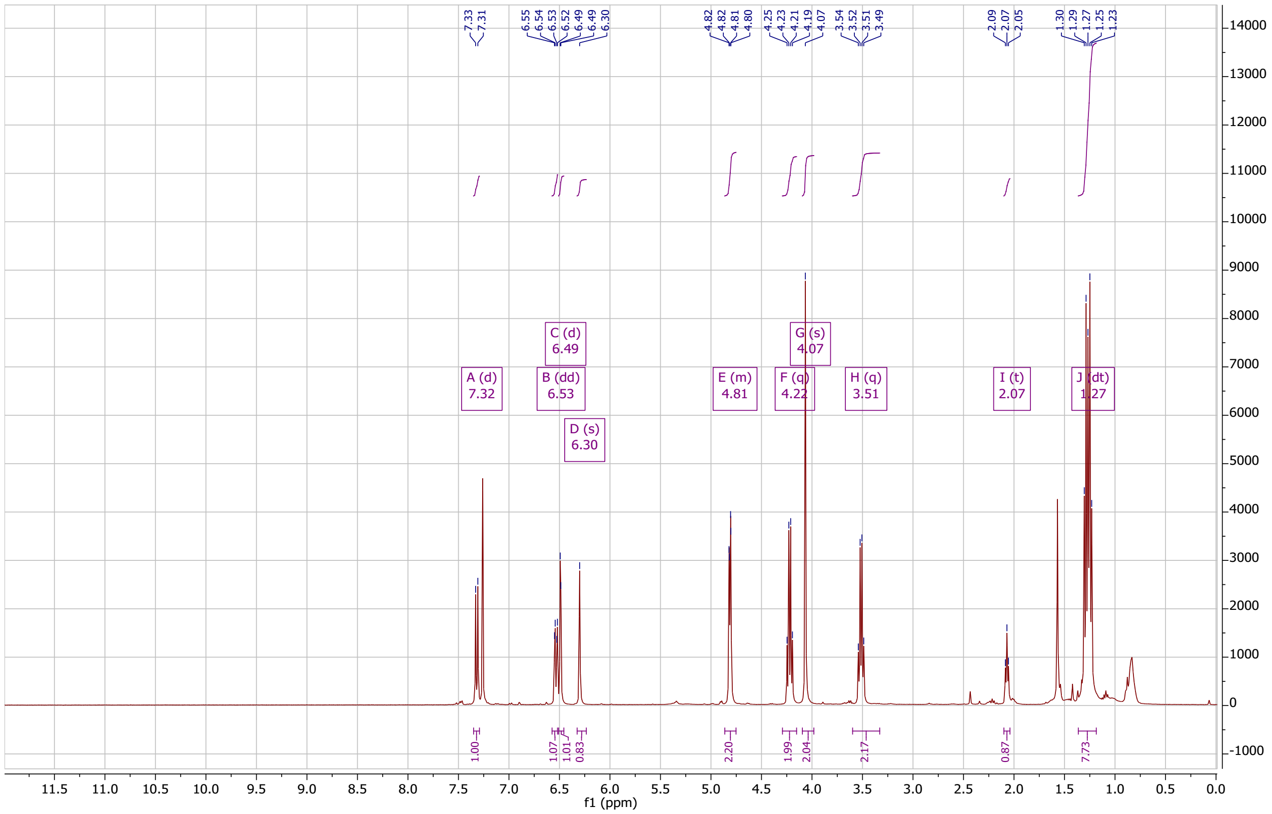

^{13}C NMR (101 MHz, Chloroform-*d*): δ 170.1 (*C*O), 162.1 (C_{CM}), 155.9 (C_{CM}), 154.2 (C_{CM}), 150.7 (C_{CM}), 124.4 (CH_{CM}), 108.8 (CH_{CM}), 107.5 (C_{CM}), 106.6 (CH_{CM}), 98.7 (CH_{CM}), 61.5 (CH_2), 61.0 (CH_2), 52.1 (CH_2), 46.7 (CH_2), 14.2 (CH_3), 12.3 (CH_3) ppm.

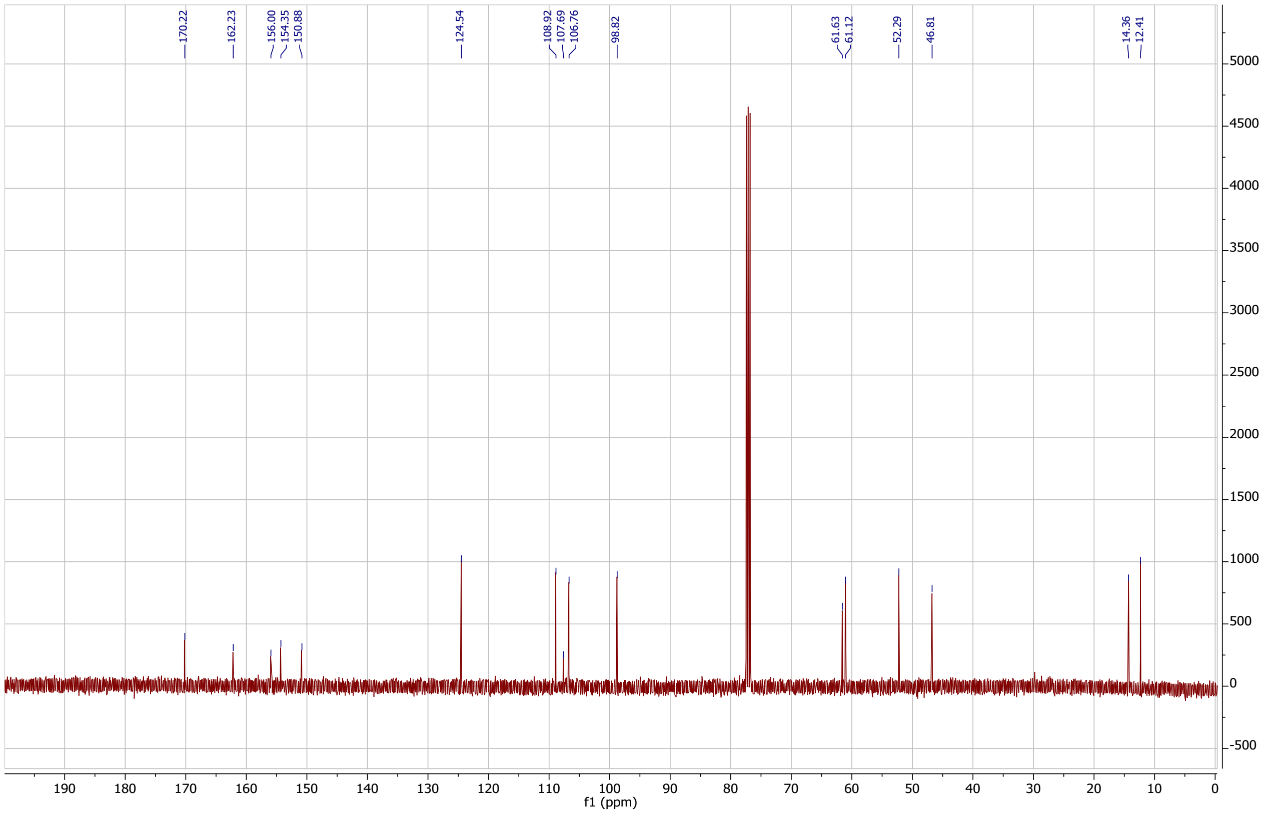

Ethyl *N*-ethyl-*N*-(4-((((4-nitrophenoxy)carbonyl)oxy)methyl)-2-oxo-2*H*-chromen-7-yl)glycinate (**5**)

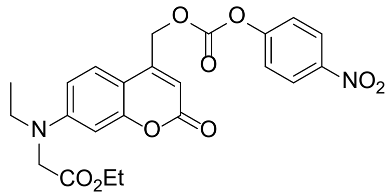

Compound **5-d** (0.10 g, 0.33 mmol, 1.0 eq) and 4-nitrophenyl chloroformate (99 mg, 0.49 mmol, 1.5 eq) were dissolved in dry DCM (2.5 mL) under argon atmosphere and dark conditions. $^{i}Pr_2NEt$ (0.20 mL, 1.32 mmol, 4.0 eq) was added and the reaction mixture was stirred at rt for 24 h. The solvent was removed under reduced pressure and the crude product was purified by FCC (EtOAc / PE, 7:3 to 5:5) to afford **5** as a yellow oil (78.7 mg, 0.17 mmol, 51% yield).

IR (neat, cm^{-1}): 2960, 1730, 1610, 1520, 1410, 1280, 1185, 1165, 1065, 860.

HRMS (nanochip-ESI/LTQ-Orbitrap) m/z: $[M + Na]^+$ Calcd for $C_{23}H_{22}N_2NaO_9^+$ 493.1218; Found 493.1207.

^{1}H NMR (400 MHz, Chloroform-*d*): δ 8.30 (d, *J* = 9.1 Hz, 2H, Ar-*H*), 7.42 (d, *J* = 9.1 Hz, 2H, Ar-*H*), 7.35 (d, *J* = 9.0, 1H, CM-*H*), 6.59 (dd, *J* = 9.0, 2.6 Hz, 1H, CM-*H*), 6.53 (d, *J* = 2.6 Hz, 1H, CM-*H*), 6.28 (s, 1H, CM-*H*), 5.40 (s, 2H, $CH_{2\,CM}$), 4.23 (q, *J* = 7.1 Hz, 2H, $CH_{2\,ester}$), 4.09 (s, 2H, N-CH_2-CO_2Et), 3.54 (q, *J* = 7.1 Hz, 2H, N-CH_2-CH_3), 1.29 (t, *J* = 7.1 Hz, 3H, $CH_{3\,ester}$), 1.27 (t, *J* = 7.2 Hz, 3H, N-CH_2-CH_3) ppm.

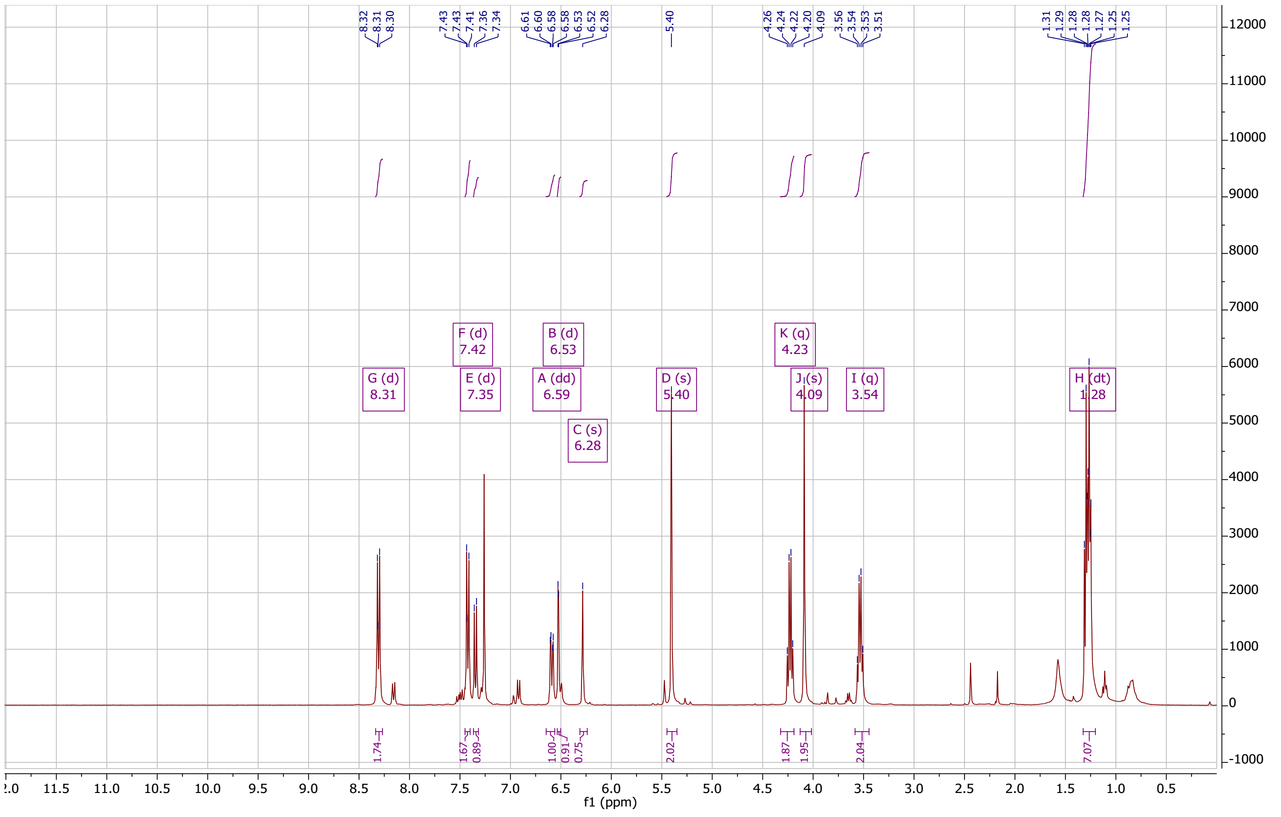

^{13}C NMR (101 MHz, Chloroform-*d*): δ 170.0 (*C*O), 161.4 (C_{CM}), 156.2 (C_{CM}), 155.3 (C_{Ar}), 152.3 (C_{CM}), 151.3 (C_{CM}), 147.7 (C_{Ar}), 125.6 (CH_{Ar}), 124.5 (CH_{CM}), 121.9 (CH_{Ar}), 109.2 (CH_{CM}), 108.3 (CH_{CM}), 107.0 (C_{CM}), 99.0 (CH_{CM}), 65.8 (CH_2), 61.7 (CH_2), 52.3 (CH_2), 46.9 (CH_2), 14.4 (CH_3), 12.4 (CH_3) ppm.

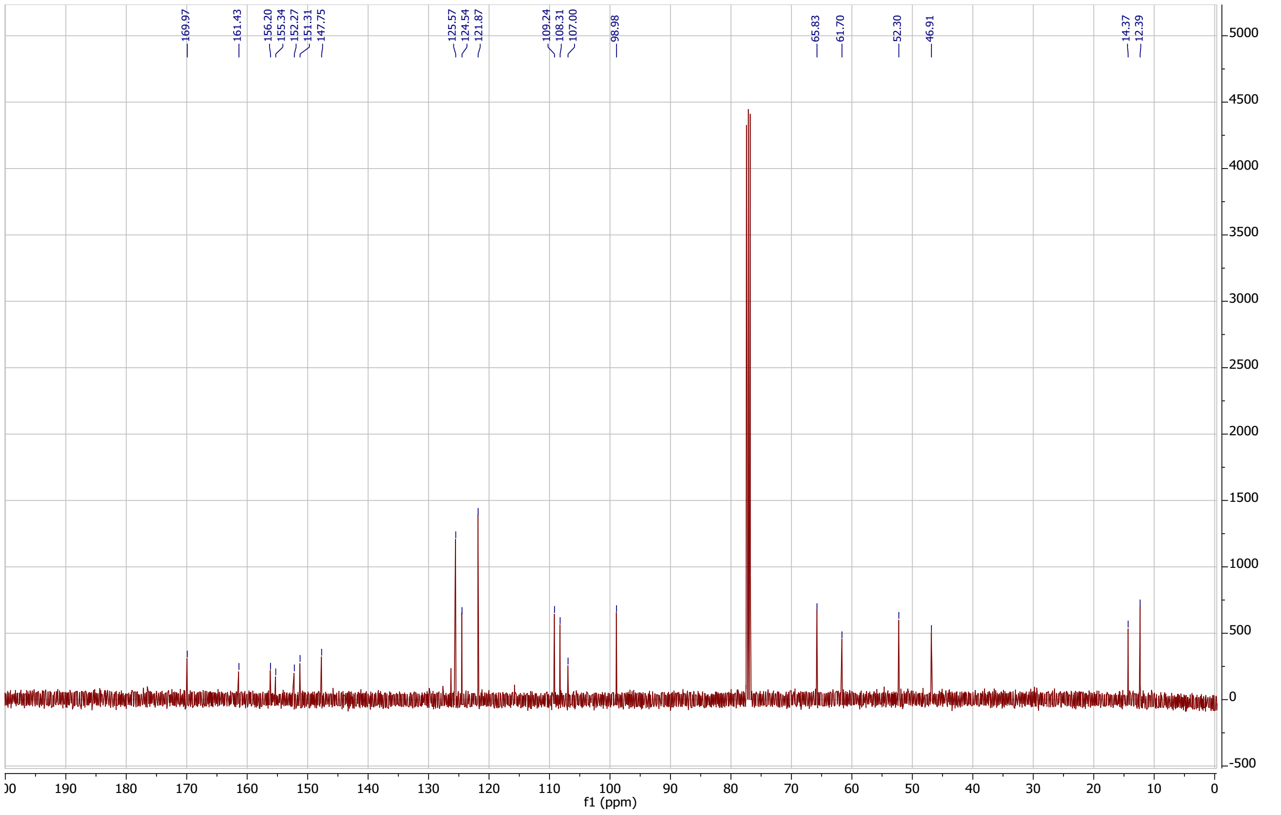

Preparation of compound 7

Protocols adapted from:

Mbua, N. E.; Guo, J.; Wolfert, M. A.; Steet, R.; Boons, G.-J. Strain-Promoted Alkyne-Azide Cycloadditions (SPAAC) Reveal New Features of Glycoconjugate Biosynthesis. *Chembiochem Eur. J. Chem. Biol.* **2011**, *12*, 1912–1921.

Wu, Y.; Zhu, H.; Zhang, B.; Liu, F.; Chen, J.; Wang, Y.; Wang, Y.; Zhang, Z.; Wu, L.; Si, L.; et al. Synthesis of Site-Specific Radiolabeled Antibodies for Radioimmunotherapy via Genetic Code Expansion. *Bioconjug. Chem.* **2016**, *27*, 2460–2468.

Scheme S3. Synthetic route toward compound **7**

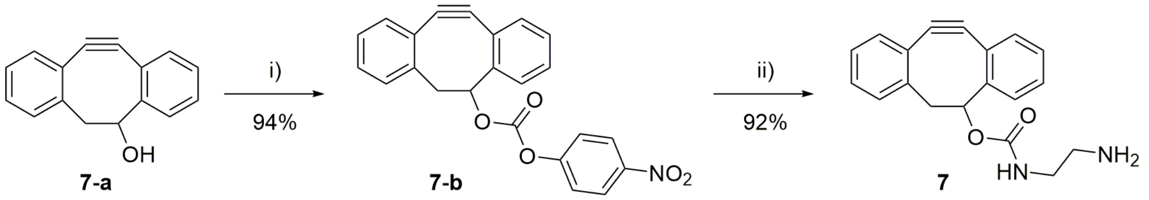

Reagents and conditions: i) 4-nitrophenyl chloroformate, pyridine, DCM, r.t., 16 h; ii) ethylenendiamine, Et_3N, DCM, r.t., 2 h.

11,12-Didehydro-5,6-dihydrodibenzo[*a*,*e*][8]annulen-5-yl 4- nitrophenyl carbonate (**7-b**)

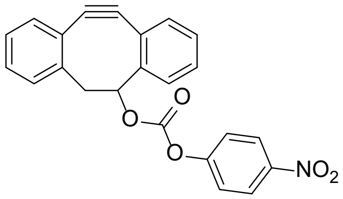

DIBO (**7-a**) (0.50 g, 2.3 mmol, 1.0 eq) and 4-nitrophenyl chloroformate (0.92 g, 4.6 mmol, 2.0 eq) were dissolved in DCM (70 mL). Pyridine (0.9 mL, 11.4 mmol, 5.0 eq) was added and the reaction mixture was stirred at rt for 16 h. The solution was washed with brine (2 X 10 mL) and the organic layer was dried over $MgSO_4$, filtered and concentrated under reduced pressure. The crude mixture was purified by FCC (PE / EtOAc, 5:1) to afford **7-b** as a white solid (0.83 g, 2.1 mmol, 94%). The analytical data were in accordance with previously reported data.

^{1}H NMR (400 MHz, Chloroform-*d*): δ 8.28 (d, *J* = 9.1 Hz, 2H, 2 X Ar-*H*), 7.62 (dq, *J* = 7.8, 1.0 Hz, 1H, DIBO-*H*), 7.47 – 7.29 (m, 9H, 7 X DIBO-*H* and 2 X Ar-*H*), 5.59 (t, *J* = 3.0 Hz, 1H, CH_{DIBO}), 3.34 (dd, *J* = 15.4, 2.2 Hz, 1H, H-CH_{DIBO}), 3.05 (dd, *J* = 15.3, 4.0 Hz, 1H, H-CH_{DIBO}) ppm.

Compound **7**

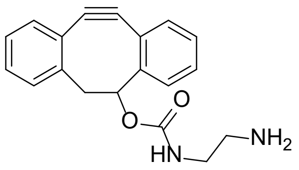

A solution of **7-b** (0.40 g, 1.04 mmol, 1.0 eq) in dry DCM (5 mL) was added dropwise to a solution of ethylenediamine (0.35 mL, 5.19 mmol, 5.0 eq) and Et_3N (0.43 mL, 3.11 mmol, 3.0 eq) in dry DCM (25 mL). The reaction mixture was stirred at rt for 2 h under argon atmosphere. The solvent was removed under reduced

pressure and the crude mixture was purified by FCC (DCM / MeOH, 5:1) to afford **7** as a pale yellow oil (0.29 g, 0.96 mmol, 92% yield). The analytical data were in accordance with previously reported data.

^{1}H NMR (400 MHz, Chloroform-*d*): δ 7.51 (d, *J* = 7.7 Hz, 1H, Ar-*H*), 7.41 – 7.24 (m, 7H, 7 X Ar-*H*), 5.50 (d, *J* = 3.3 Hz, 1H, C*H*), 5.38 (s, 1H, N*H*), 3.26 (q, *J* = 6.0 Hz, 2H, NH-CH_2-CH_2-NH_2), 3.17 (dd, *J* = 15.1, 2.2 Hz, 1H, HC-*H*), 2.99 – 2.90 (m, 1H, HC-*H*), 2.90 – 2.83 (m, 2H, CH_2-NH_2) ppm.

Preparation of compound 2

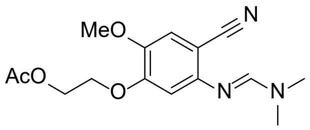

IR (neat, cm^{-1}): 2935, 2360, 2210, 1740, 1630, 1375, 1215.

^{1}H NMR (400 MHz, Chloroform-d) δ 7.55 (s, 1 H, Ar-N=C*H*), 6.92 (s, 1 H, H_{Ar}), 6.46 (s, 1 H, H_{Ar}), 4.42 (t, ^{3}J = 4.6 Hz, 2 H, CH_2-OAr), 4.21 (t, ^{3}J = 4.8 Hz, 2 H, CH_2-OAc), 3.80 (s, 3 H, CH_3-OAr), 3.80 (s, 6 H, 2 x CH_3-N), 2.06 (s, 3 H, CH_3-CO) ppm.

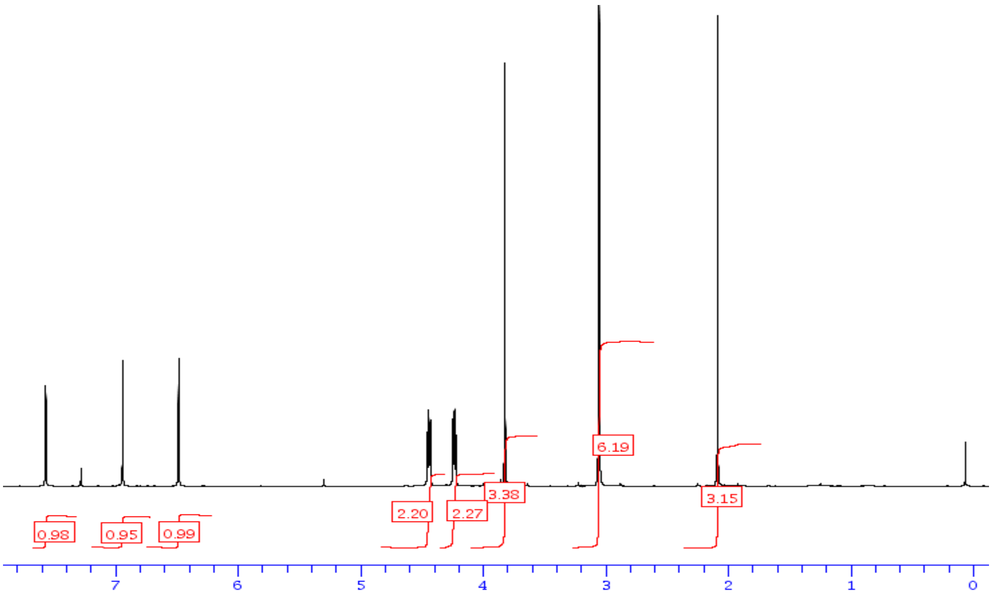

^{13}C NMR (101 MHz, Chloroform-d) δ 170.9 (*C*O), 153.8 (*C*H), 151.8 (C_{Ar}), 150.2 (C_{Ar}), 143.7 (C_{Ar}), 119.7 (*C*N), 115.0 (CH_{Ar}), 105.6 (CH_{Ar}), 96.0 (C_{Ar}), 66.8 (*C*H_2), 62.4 (*C*H_2), 56.4 (*C*H_3), 40.3 (*C*H_3), 34.6 (*C*H_3), 20.9 (*C*H_3) ppm.

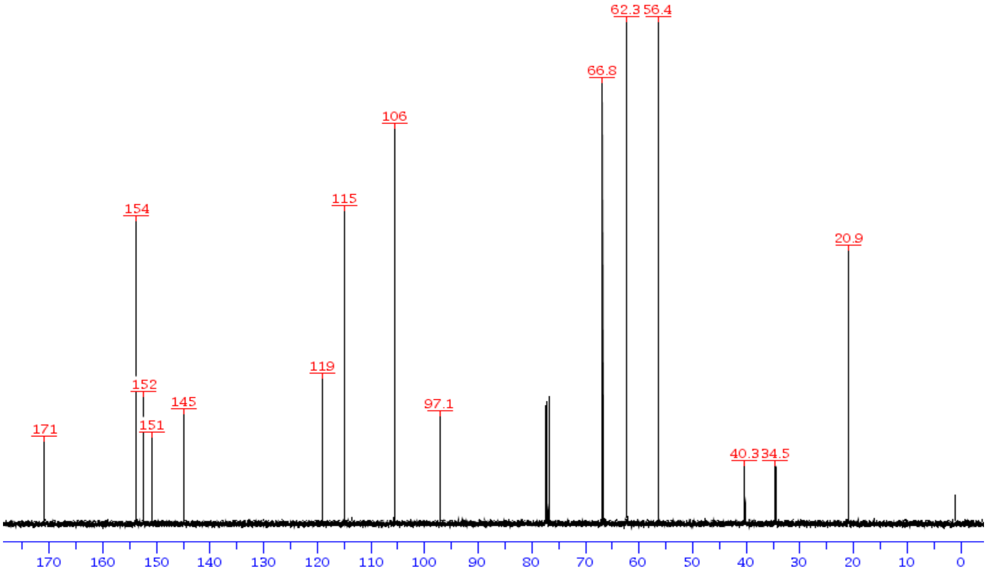

Preparation of compound 3

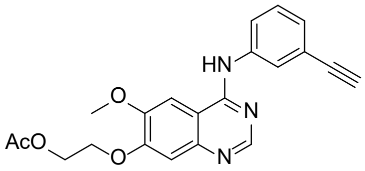

IR (neat, cm^{-1}): 2970, 2360, 1735, 1425, 1230.

^{1}H NMR (400 MHz, Dimethyl sulfoxide-d$_6$) δ 9.53 (s, 1H, N*H*), 8.51 (s, 1H, *H*$_{Ar}$), 7.99 (t, *J* = 1.6 Hz, 1H, *H*$_{Ar}$), 7.90 (dd, *J* = 8.0, 1.9 Hz, 1H, *H*$_{Ar}$), 7.86 (s, 1H, *H*$_{Ar}$), 7.41 (t, *J* = 7.9 Hz, 1H, *H*$_{Ar}$), 7.24 (s, 1H, *H*$_{Ar}$), 7.21 (m, 1H, *H*$_{Ar}$), 4.41 (m, 2H, C*H*$_2$-OAc), 4.36 (m, 2H, C*H*$_2$-OAr), 4.21 (s, 1H, *H*C≡C), 3.98 (s, 3H, C*H*$_3$-OAr), 2.07 (s, 3H, C*H*$_3$-CO) ppm.

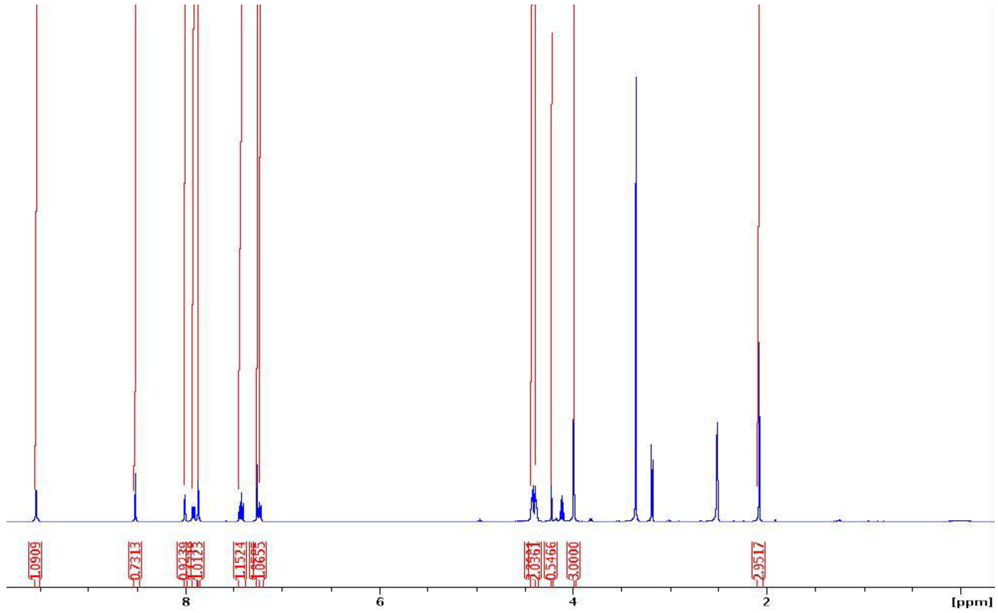

^{13}C NMR (101 MHz, Dimethyl sulfoxide-d$_6$) *δ* 170.9 (*C*O), 156.6 (*C$_{Ar}$*), 153.6 (*C*H$_{Ar}$), 152.6 (*C*H$_{Ar}$), 149.9 (*C$_{Ar}$*), 147.2 (*C$_{Ar}$*), 139.0 (*C*H$_{Ar}$), 129.2 (*C*H$_{Ar}$), 126.9 (*C$_{Ar}$*), 125.2 (*C*H$_{Ar}$), 122.8 (*C*H$_{Ar}$), 121.8 (*C$_{Ar}$*), 109.9 (*C$_{Ar}$*), 108.7 (*C*H$_{Ar}$), 101.8 (*C$_{Ar}$*), 84.4 (*C$_q$*), 80.8 (*C*H), 67.1 (*C*H$_2$), 62.7 (*C*H$_2$), 55.3 (*C*H$_3$), 21.4 (*C*H$_3$) ppm.

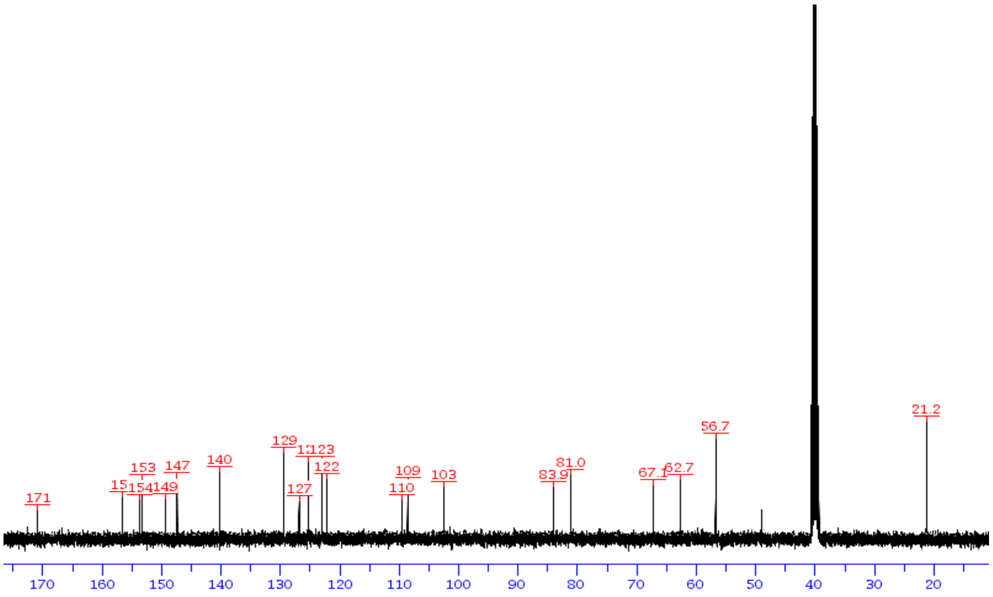

Preparation of compound 4

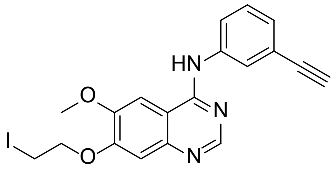

IR (neat, cm^{-1}): 3430, 2390, 1630, 1505, 1435, 1245, 1220, 979, 878, 815.

^{1}H NMR (400 MHz, Chloroform-d) δ 8.60 (s, 1H, H_{Ar}), 7.86 (t, J = 1.8 Hz, 1H, H_{Ar}), 7.77 (dt, J = 8.2, 1.5 Hz, 1H, H_{Ar}), 7.35 (t, J = 7.9 Hz, 1H, H_{Ar}), 7.31 – 7.27 (m, 1H, H_{Ar}), 7.24 (s, 1H, H_{Ar}), 7.17 (s, 1H, H_{Ar}), 4.40 (t, J = 7.1 Hz, 2H, CH_2-OAr), 4.04 (s, 3H, CH_3-OAr), 3.55 – 3.46 (m, 2H, CH_2-I), 3.10 (s, 1H, HC≡C) ppm.

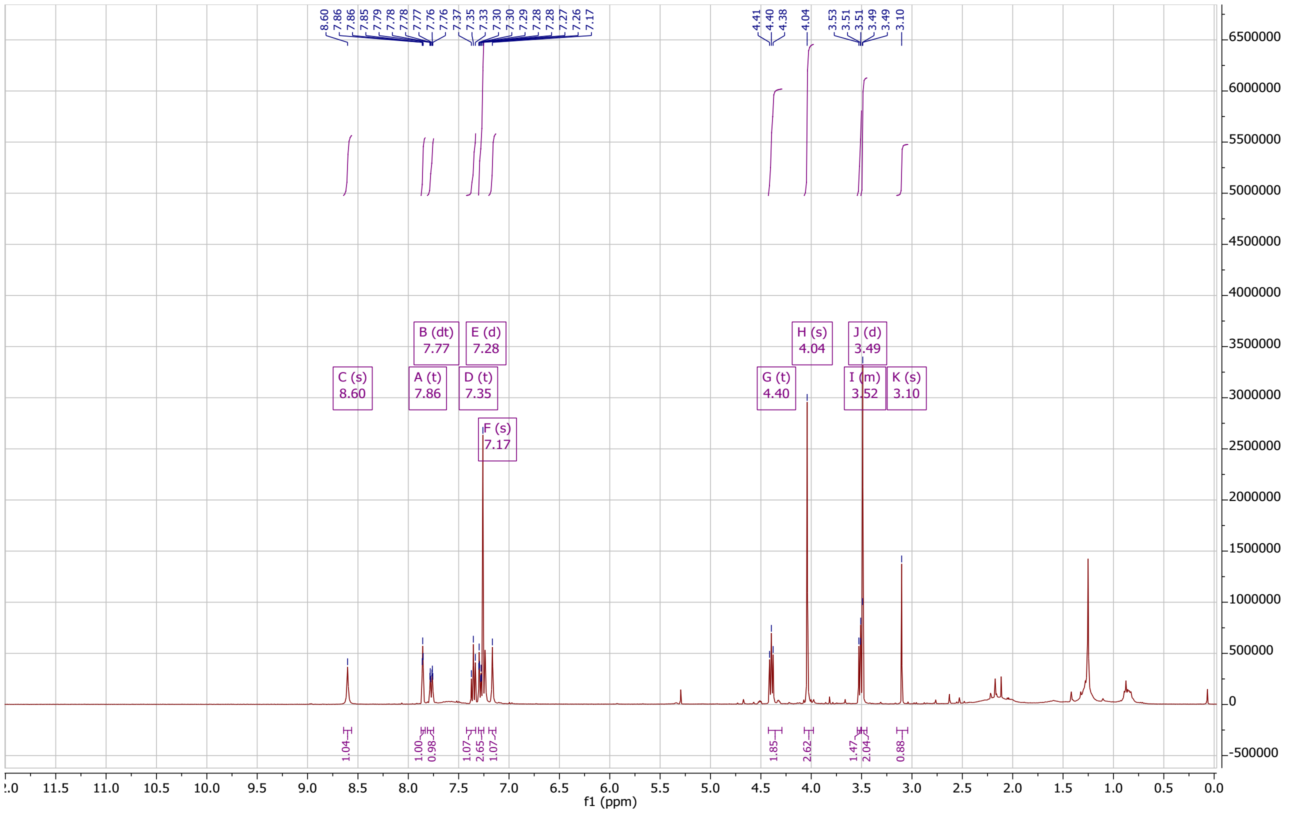

^{13}C NMR (101 MHz, Chloroform-d) δ 168.5 (C_{Ar}), 156.3 (C_{Ar}), 153.6 (CH_{Ar}), 150.0 (C_{Ar}), 143.7 (C_{Ar}), 138.4 (C_{Ar}), 129.2 (CH_{Ar}), 128.4 (CH_{Ar}), 125.5 (CH_{Ar}), 123.1 (CH_{Ar}), 122.8 (C_{Ar}), 108.1 (CH_{Ar}), 100.6 (CH_{Ar}), 83.3 (C_{alkyne}), 77.8 (CH_{alkyne}), 69.6 (CH_2), 57.0 (CH_3), -0.5 (CH_2) ppm. One carbon is not resolved.

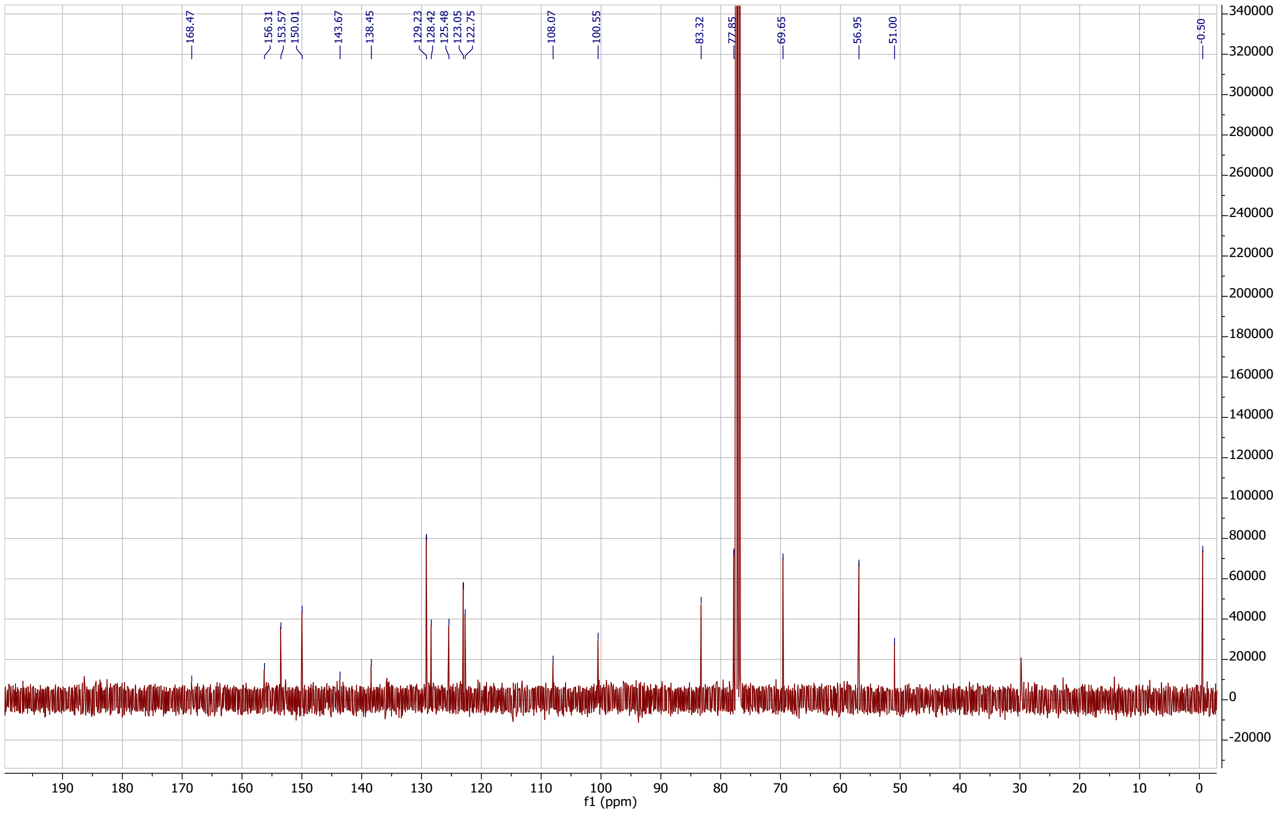

Data for intermediate alcohol

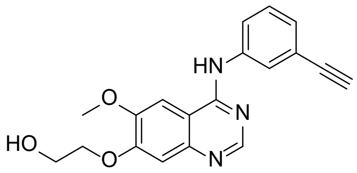

IR (neat, cm^{-1}): 3300, 2970, 2360, 1740, 1425, 1220, 1025.

HRMS (nanochip-ESI/LTQ-Orbitrap) m/z: calcd for $C_{19}H_{17}N_3O_3$ 336.1348, found: 336.1348.

^{1}H NMR (400 MHz, Dimethyl sulfoxide-d$_6$) δ 9.49 (s, 1H, N*H*), 8.50 (s, 1H, *H*$_{Ar}$), 7.99 (m, 1 H, C*H*$_{Ar}$(2')), 7.91 (dd, ^3J = 8.0 Hz, ^4J = 2.1 Hz, 1 H, C*H*$_{Ar}$(6')), 7.79 (s, 1 H, C*H*$_{Ar}$(8)), 7.41 (t, ^3J = 8.0 Hz, 1 H, C*H*$_{Ar}$(5')), 7.21 (m, 2 H, C*H*$_{Ar}$(4'), C*H*$_{Ar}$(5)), 4.94 (t, ^3J = 5.2 Hz, 1 H, *H*O-CH$_2$), 4.20 (s, 1 H, *H*C≡C-Ar), 4.14 (t, ^3J = 4.9 Hz, 2 H, C*H*$_2$-OAr), 3.97 (s, 3 H, C*H*$_3$-OAr), 3.81 (dt, ^3J = 5.1 Hz, ^3J = 5.1 Hz, 2 H, C*H*$_2$-OH) ppm.

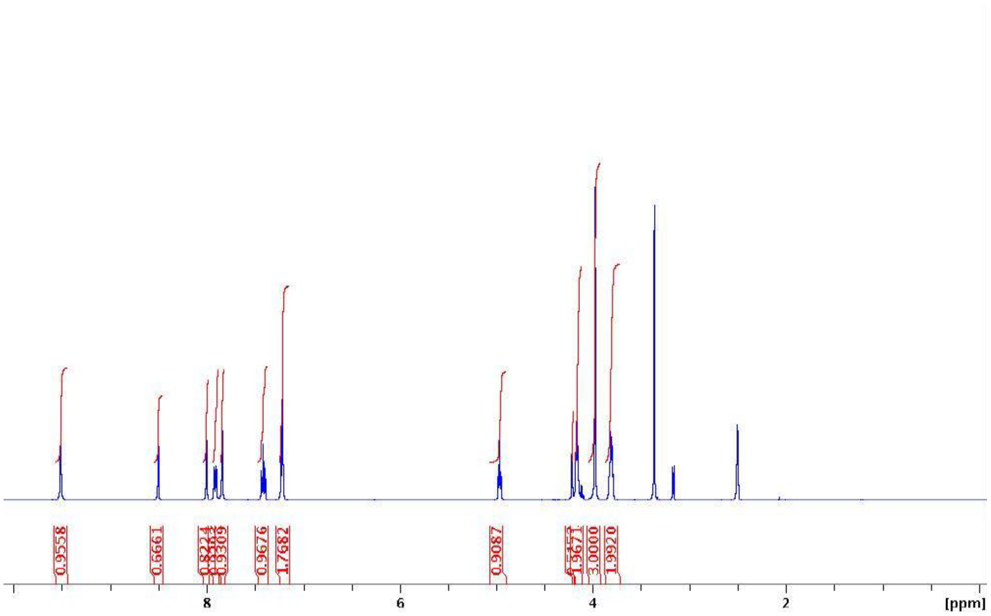

^{13}C NMR (101 MHz, Dimethyl sulfoxide-d$_6$) δ 156.6 (C_{Ar}), 154.2 (CH_{Ar}), 152.5 (CH_{Ar}), 149.5 (C_{Ar}), 147.5 (C_{Ar}), 140.3 (CH_{Ar}), 129.4 (CH_{Ar}), 126.8 (C_{Ar}), 125.2 (CH_{Ar}), 123.0 (CH_{Ar}), 121.9 (C_{Ar}), 109.3 (C_{Ar}), 108.3 (CH_{Ar}), 102.3 (C_{Ar}), 84.0 (C_q), 80.7 (CH), 71.3 (CH$_2$), 60.7 (CH$_2$), 56.3 (CH$_3$) ppm.

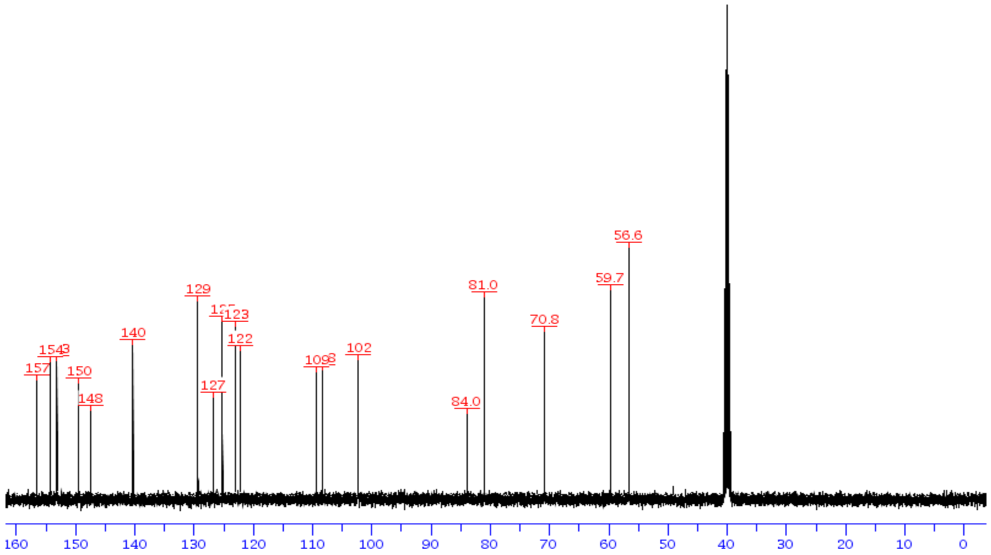

Data for intermediate mesylate

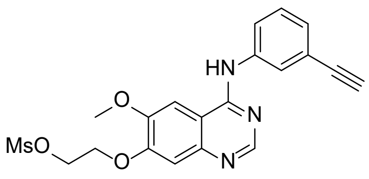

IR (neat, cm^{-1}): 2920, 285o, 1615, 1505, 1460, 1425, 1330, 1250, 1170, 980, 925, 810.

HRMS (nanochip-ESI/LTQ-Orbitrap) m/z: [M + H]$^+$ Calcd for $C_{20}H_{20}N_3O_5S^+$ 414.1118; Found 414.1112.

^{1}H NMR (400 MHz, Dimethyl sulfoxide-d_6): δ 9.53 (s, 1H, NH), 8.51 (s, 1H, H_{Ar}), 8.00 (d, J = 5.0 Hz, 1H, H_{Ar}), 7.91 (d, J = 8.1 Hz, 1H, H_{Ar}), 7.87 (s, 1H, H_{Ar}), 7.41 (t, J = 7.9 Hz, 1H, H_{Ar}), 7.26 (s, 1H, H_{Ar}), 7.21 (d, J = 7.7 Hz, 1H, H_{Ar}), 4.64 (dt, J = 4.2, 2.2 Hz, 2H, CH_2-OMs), 4.45 (dd, J = 5.4, 2.8 Hz, 2H, CH_2-OAr), 4.20 (s, 1H, HC≡C), 3.98 (s, 3H, CH_3-OAr), 3.29 (s, 3H, C$H_{3\ \mathrm{mesyl}}$) ppm.

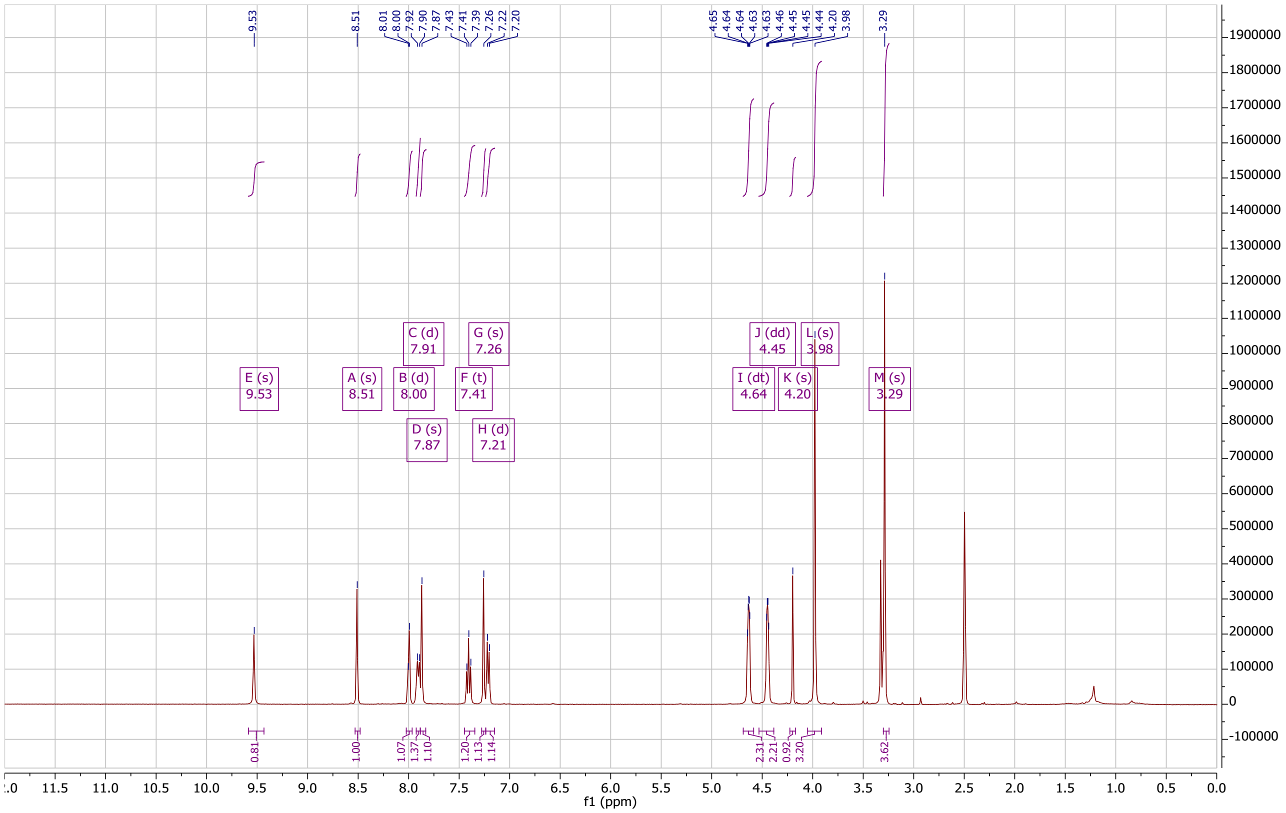

^{13}C NMR (101 MHz, Dimethyl sulfoxide-d_6): δ 156.1 (C_{Ar}), 152.8 (CH$_{Ar}$), 152.8 (C_{Ar}), 148.8 (C_{Ar}), 146.9 (C_{Ar}), 139.8 (C_{Ar}), 128.9 (CH$_{Ar}$), 126.4 (CH$_{Ar}$), 124.8 (CH$_{Ar}$), 122.6 (CH$_{Ar}$), 121.7 (C_{Ar}), 109.2 (C_{Ar}), 108.2 (CH$_{Ar}$), 102.2 (CH$_{Ar}$), 83.5 (C_{alkyne}), 80.6 (CH$_{alkyne}$), 68.6 (CH$_2$), 66.6 (CH$_2$), 56.3 (CH$_3$), 36.9 (CH$_3$) ppm.

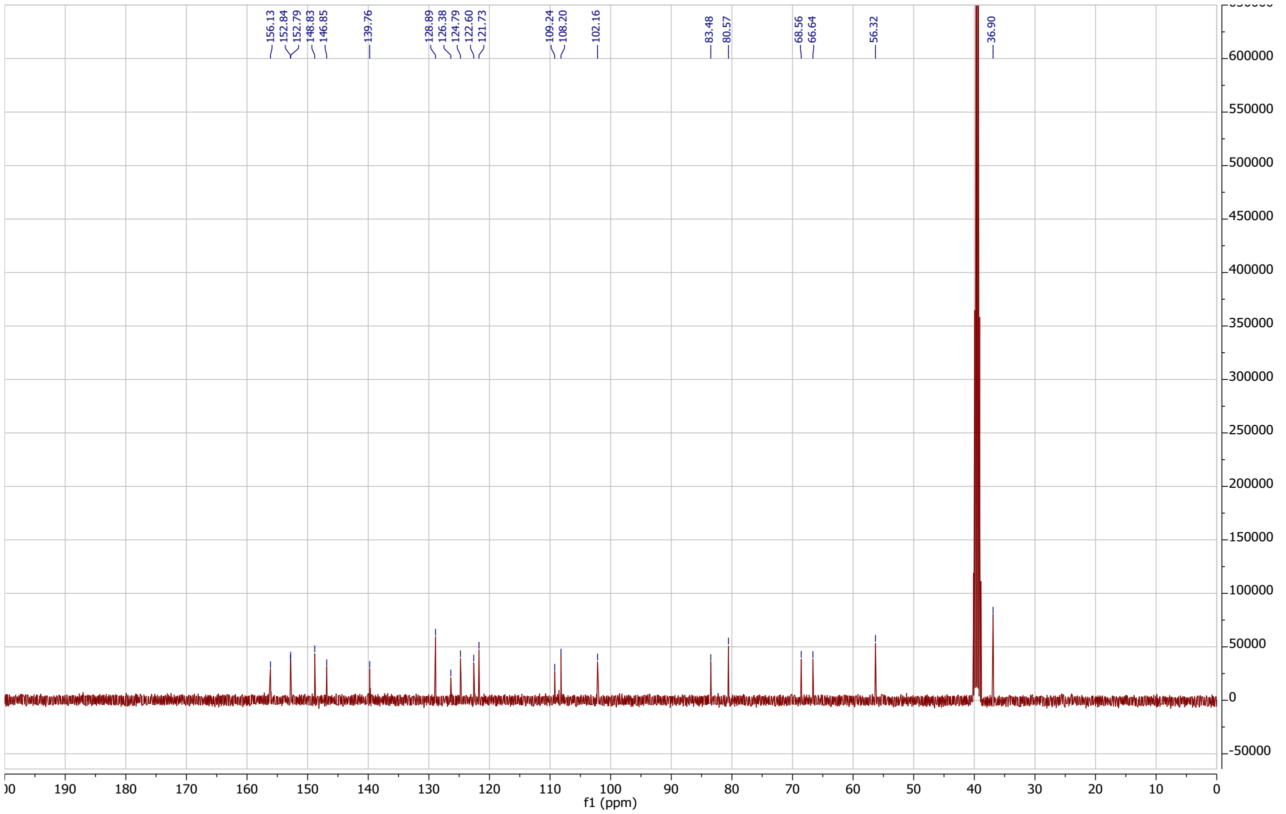

Preparation of ELA

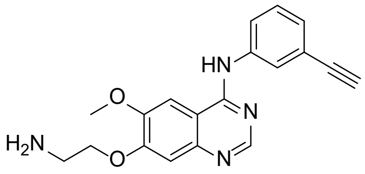

IR (neat, cm^{-1}): 3455, 2925, 1625, 1525, 1510, 1455, 1430, 1280, 1245, 1210, 1155, 995.

^{1}H NMR (400 MHz, Methanol-d$_4$) δ 8.51 (s, 1H, H_{Ar}), 7.94 (t, J = 1.9 Hz, 1H, H_{Ar}), 7.90 (s, 1H, H_{Ar}), 7.80 (dd, J = 8.2, 2.3 Hz, 1H, H_{Ar}), 7.41 (t, J = 7.9 Hz, 1H, H_{Ar}), 7.31 (dt, J = 7.6, 1.3 Hz, 1H, H_{Ar}), 7.26 (s, 1H, H_{Ar}), 4.49 – 4.40 (m, 2H, CH_2-OAr), 4.12 (s, 3H, CH_3-OAr), 3.55 (s, 1H, HC≡C), 3.53 – 3.47 (m, 2H, CH_2-NH$_2$) ppm.

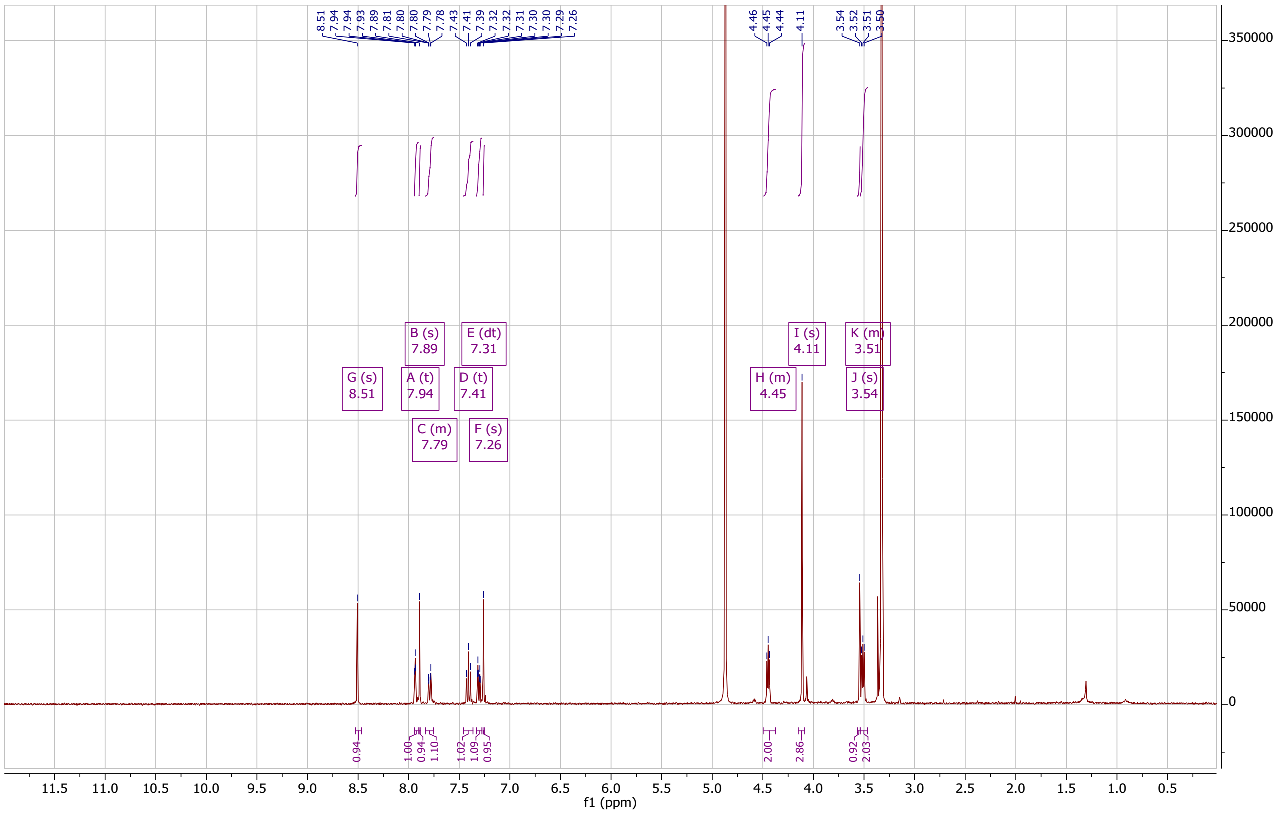

^{13}C NMR (101 MHz, Methanol-d_4): δ 160.1 (C$_{Ar}$), 155.9 (C$_{Ar}$), 153.4 (C$_{Ar}$), 152.6 (CH$_{Ar}$), 149.6 (C$_{Ar}$), 141.0 (C$_{Ar}$), 128.6 (CH$_{Ar}$), 127.7 (CH$_{Ar}$), 125.9 (CH$_{Ar}$), 123.1 (CH$_{Ar}$), 122.9 (C$_{Ar}$), 121.2 (C$_{Ar}$), 107.1 (CH$_{Ar}$), 101.9 (CH$_{Ar}$), 82.8 (C_{alkyne}), 77.4 (CH$_{alkyne}$), 65.2 (CH$_2$), 55.7 (CH$_3$), 38.7 (CH$_2$).

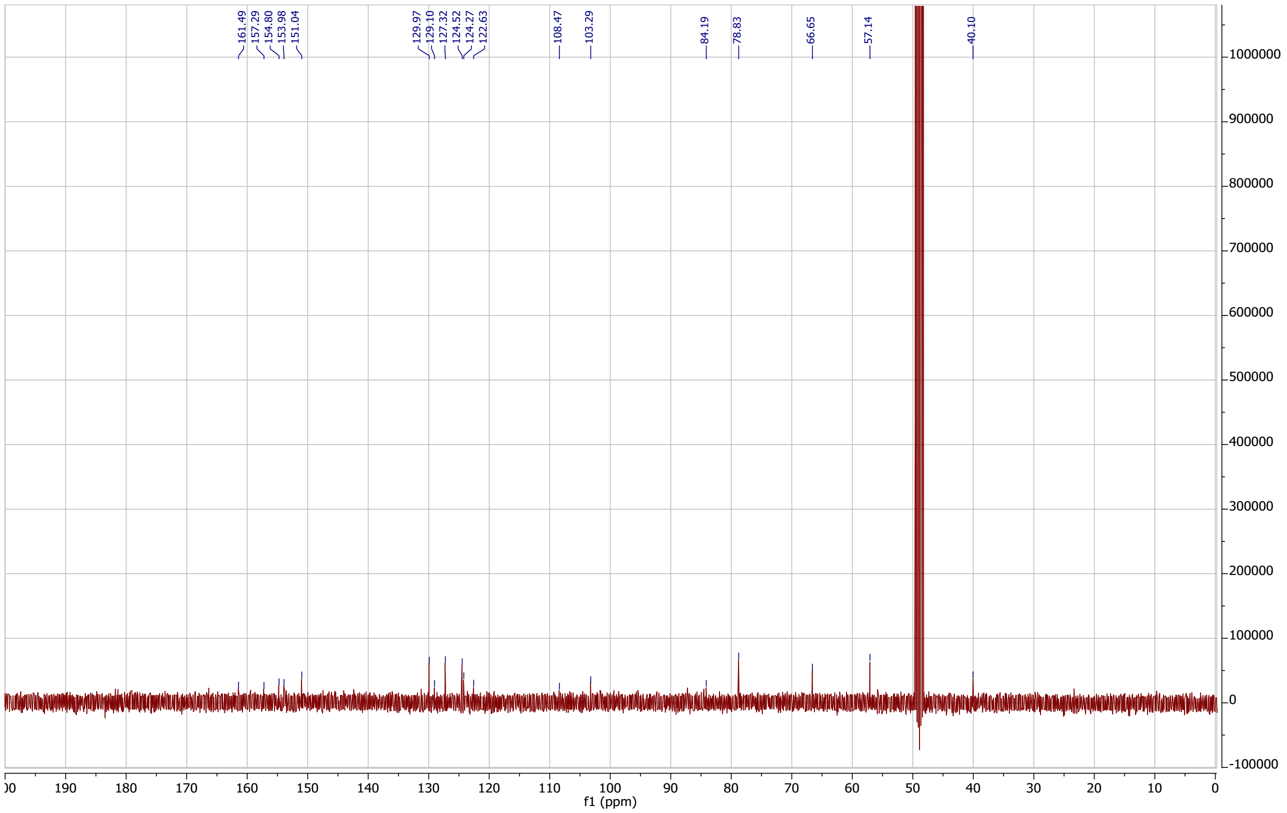

161.49
157.29
154.80
153.98
151.04
129.97
129.10
127.32
124.52
124.27
122.63
108.47
103.29
84.19
78.83
66.65
57.14
40.10
f1 (ppm)

Preparation of compound 6

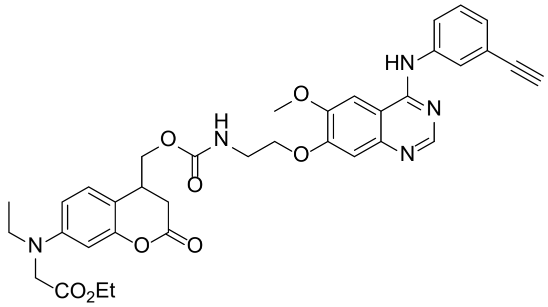

IR (neat, cm^{-1}): 3345, 1960, 1620, 1700, 1155, 835, 770, 715.

^{1}H NMR (400 MHz, Dimethyl sulfoxide-d$_6$) δ 9.51 (s, 1H, NH_{ELA}), 8.50 (s, 1H, H_{Ar}), 7.99 (d, J = 1.9 Hz, 1H, H_{Ar}), 7.90 (m, 2H, H_{Ar}), 7.81 (t, J = 5.6 Hz, 1H, N$H_{carbamate}$), 7.48 (m, 1H, CM-H), 7.41 (t, J = 7.8 Hz, 1H, H_{Ar}), 7.22 (dd, J = 16.9, 9.5 Hz, 2H, H_{Ar}), 6.64 (dd, J = 9.1, 2.6 Hz, 1H, CM-H), 6.53 (d, J = 2.5 Hz, 1H, CM-H), 6.05 (s, 1H, CM-H), 5.25 (s, 2H, C$H_{2\,CM}$), 4.28 (s, 2H, N-CH_2-CO_2Et), 4.23 (t, J = 5.5 Hz, 2H, NH-CH_2-CH_2-OAr), 4.20 (s, 1H, HC≡C), 4.13 (q, J = 7.2 Hz, 2H, C$H_{2\,ester}$), 3.97 (s, 3H, CH_3-OAr), 3.49 (d, J = 5.0 Hz, 4H, N-CH_2-CH_3 and NH-CH_2-CH_2-OAr), 1.20 (m, 3H, N-CH_2-CH_3), 1.12 (t, J = 7.0 Hz, 3H, C$H_{3\,ester}$) ppm.

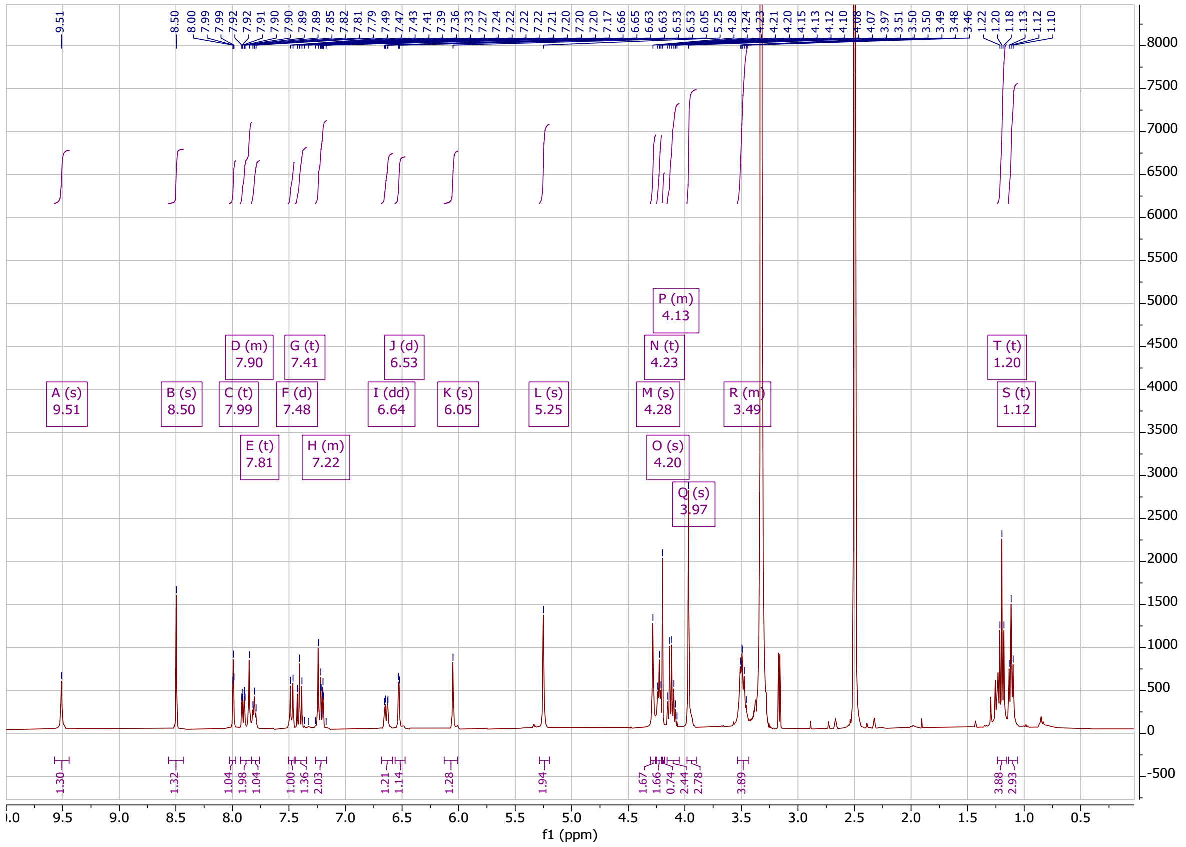

^{13}C NMR (101 MHz, Dimethyl sulfoxide-d_6): δ 170.5 (CO_2Et), 161.1 (CO_{CM}), 156.6 (C_{ELA}), 155.8 (C_{CM}), 153.8 (C_{erlo}), 153.2 (CH_{ELA}), 152.2 ($CO_{carbamate}$), 151.4 (C_{CM}), 149.5 (C_{ELA}), 147.4 (C_{ELA}), 140.3 (C_{ELA}), 129.3 (CH_{ELA}), 126.8 (CH_{ELA}), 125.7 (CH_{CM}), 125.2 (CH_{ELA}), 123.1 (CH_{ELA}), 122.2, 109.5 (CH_{CM}), 108.5 (CH_{ELA}), 106.7 (CH_{CM}), 106.5 (C_{CM}), 105.3 (C_{ELA}), 102.7 (CH_{ELA}), 98.0 (CH_{CM}), 84.0 (C_{alkyne}), 81.0 (CH_{alkyne}), 67.6 ($CH_{2\,ELA}$), 61.5 ($CH_{2\,CM}$), 61.1 ($CH_{2\,CM}$), 56.8 ($CH_{3\,ELA}$), 51.9 ($CH_{2\,CM}$), 46.2 ($CH_{2\,CM}$), 40.2 ($CH_{2\,ELA}$), 14.6 ($CH_{3\,CM}$), 12.5 ($CH_{3\,CM}$). Two carbons are not resolved.

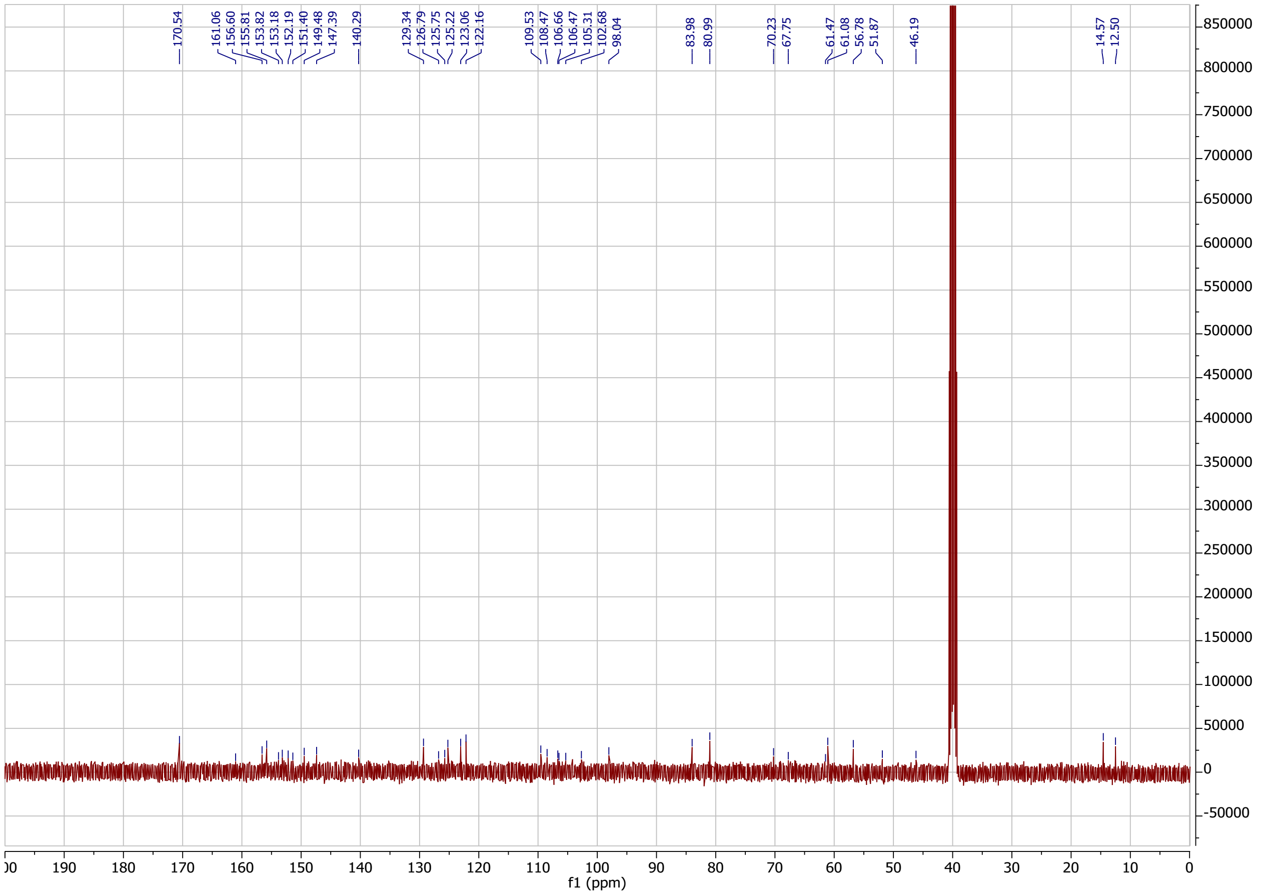
170.54
161.06
156.60
155.81
153.82
153.18
152.19
151.40
149.48
147.39
140.29
129.34
126.79
125.75
125.22
123.06
122.16
109.53
108.47
106.66
106.47
105.31
102.68
98.04
83.98
80.99
70.23
67.75
61.47
61.08
56.78
51.87
46.19
14.57
12.50
f1 (ppm)

Preparation of CM-ELA

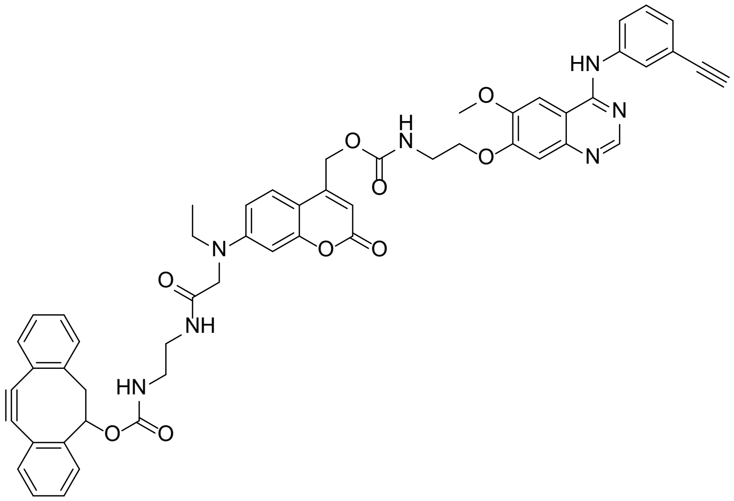

IR (neat, cm^{-1}): 2955, 2920, 1705, 1655, 1620, 1575, 1530, 1425, 1260, 1085, 1020, 795, 770.

^{1}H NMR (400 MHz, Dimethyl sulfoxide-d$_6$) δ 9.54 (s, 1H, NH_{ELA}), 9.45 (d, J = 7.9 Hz, 1H, NH_{amide}), 8.50 (d, J = 5.4 Hz, 1H, H_{Ar}), 7.99 (d, J = 12.5 Hz, 1H, H_{Ar}), 7.94 – 7.86 (m, 2H, H_{Ar}), 7.81 (d, J = 8.4 Hz, 1H, H_{Ar}), 7.60 (dt, J = 11.5, 5.7 Hz, 1H, N$H_{carbamate}$), 7.56 – 7.49 (m, 1H, CM-H and $H_{Ar\text{-}cyclooctyne}$), 7.47 – 7.27 (m, 7H, H_{Ar}), 7.26 – 7.18 (m, 2H, H_{Ar}), 6.56 (dd, J = 21.0, 8.6 Hz, 1H, CM-H), 6.51 – 6.40 (m, 1H, CM-H), 6.04 (d, J = 3.1 Hz, 1H, CM-H), 5.27 (m, 2H, N$H_{carbamate}$ and H_{DIBO}), 5.23 (s, 2H, CH_2), 4.39 (t, J = 5.6 Hz, 1H, NH-CH_2-CH$_2$-OAr), 4.23 (t, J = 5.9 Hz, 1H, NH-CH_2-CH$_2$-OAr), 4.20 (d, J = 5.1 Hz, 1H, H_{alkyne}), 3.97 (s, 3H, CH_3-O), 3.96 – 3.84 (m, 4H, N-CH_2-CONHR and NH-CH$_2$-CH_2-OAr), 3.58 – 3.47 (m, 2H, N-CH_2-CH$_3$), 3.17 (q, J = 6.3, 5.6 Hz, 3H, NH-CH_2-CH$_2$-NHCO$_2$R and H_{DIBO}), 3.06 (q, J = 7.6, 6.8 Hz, 2H, NH-CH$_2$-CH_2-NHCO$_2$R), 2.75 (dd, J = 15.0, 4.4 Hz, 1H, H_{DIBO}), 1.06 (t, J = 7.0 Hz, 1H, R$_2$N-CH$_2$-CH_3) ppm.

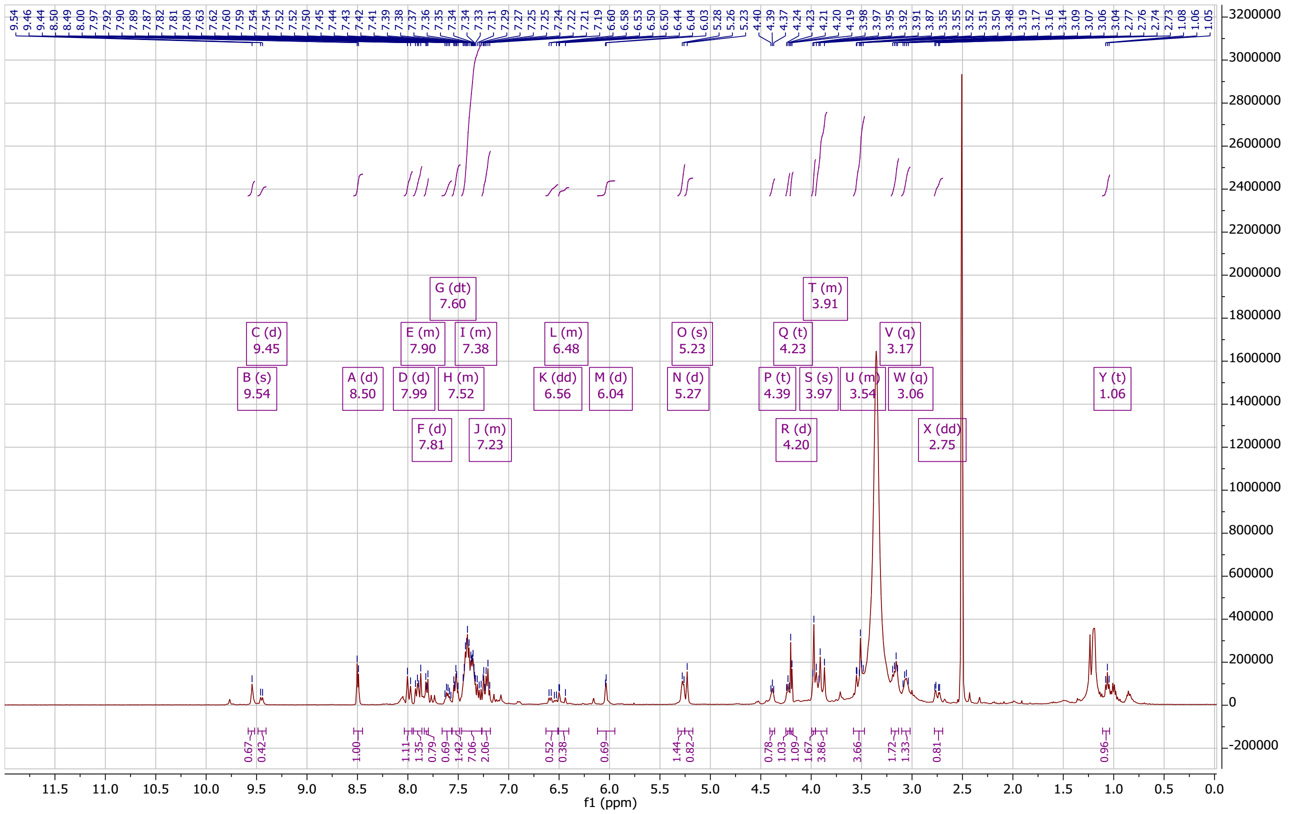

^{13}C NMR (101 MHz, Dimethyl sulfoxide-d_6): δ 171.2 (CO_{amide}), 169.4 CO_{amide}), 159.8 (CO_{CM}), 156.6 (C_{ELA}), 155.8 (C_{CM}), 155.8 (C_{ELA}), 155.8, 153.2 C_{ELA}), 152.1 (CH_{ELA}), 151.3 (C_{CM}), 149.5 (C_{DIBO}), 142.8 (C_{ELA}), 146.1 (C_{ELA}), 140.3 (C_{ELA}), 129.3 (CH_{ELA}), 128.9 (CH_{DIBO}), 127.8 (CH_{DIBO}), 127.8 (CH_{DIBO}), 126.5 (CH_{ELA}), 126.2 (CH_{DIBO}), 125.2 (CH_{ELA}), 124.3 (CH_{CM}), 123.9 (CH_{DIBO}), 123.4 (C_{DIBO}), 123.0 (CH_{ELA}), 122.2 (CH_{DIBO}), 120.8 (C_{DIBO}), 119.0 (C_{DIBO}), 113.0 (CH_{DIBO}), 109.5 (CH_{CM}), 108.5 (CH_{ELA}), 107.1 (C_{CM}), 105.6 (CH_{CM}), 102.6 (CH_{ELA}), 98.0 (CH_{CM}), 92.0 (C_{DIBO}), 84.0 (C_{ELA}), 81.0 (CH_{alkyne}), 75.9 (CH_{DIBO}), 68.8 ($CH_{2\ ELA}$), 61.9 (CH_{2CM}), 56.6 ($CH_{3\ ELA}$), 53.5 ($CH_{2\ CM}$), 46.1 ($CH_{2\ CM}$), 45.9 ($CH_{2\ DIBO}$), 40.9 ($CH_{2\ ELA}$), 40.7 ($CH_{2\ DIBO}$), 39.1 ($CH_{2\ DIBO}$), 12.1 ($CH_{3\ CM}$). Five carbons were not resolved.

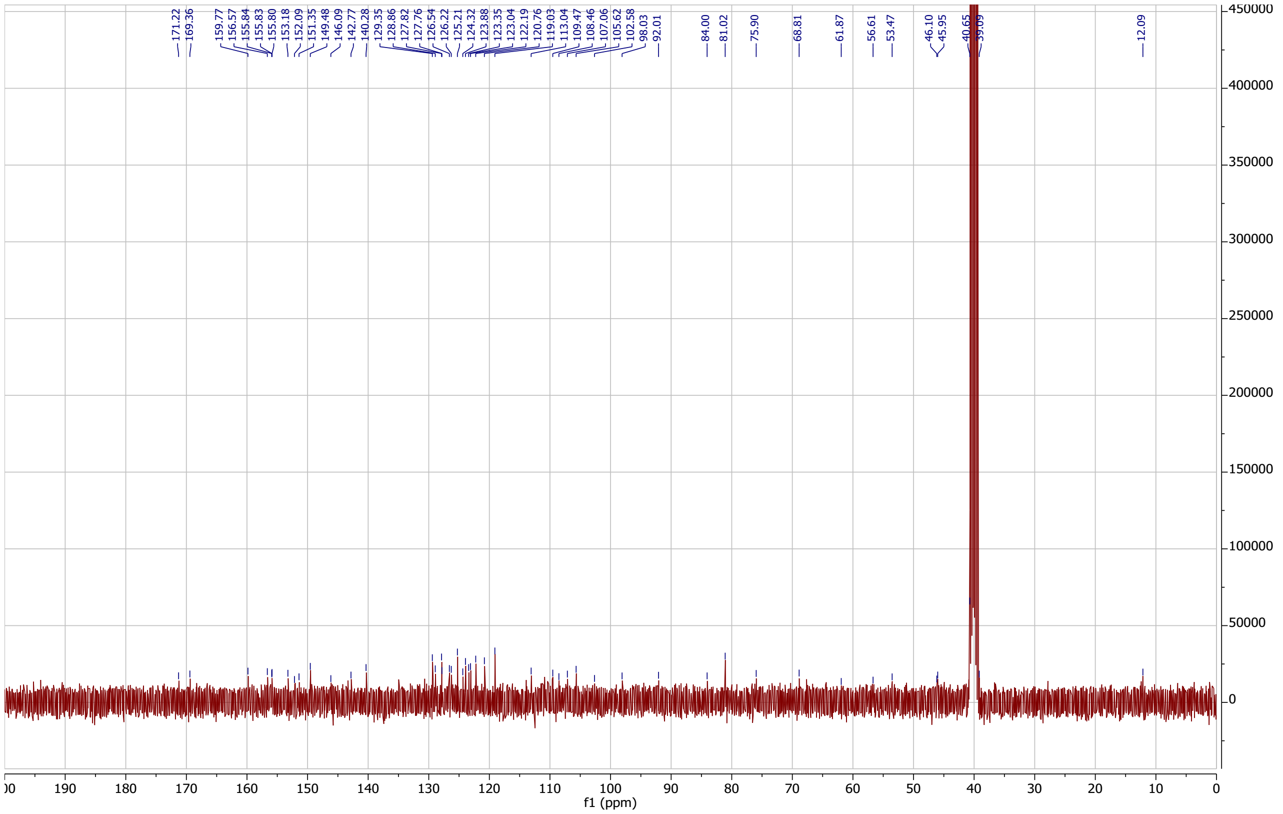

Preparation of **DIBO-ELA**

Scheme S4. Synthetic route towards **DIBO-ELA**

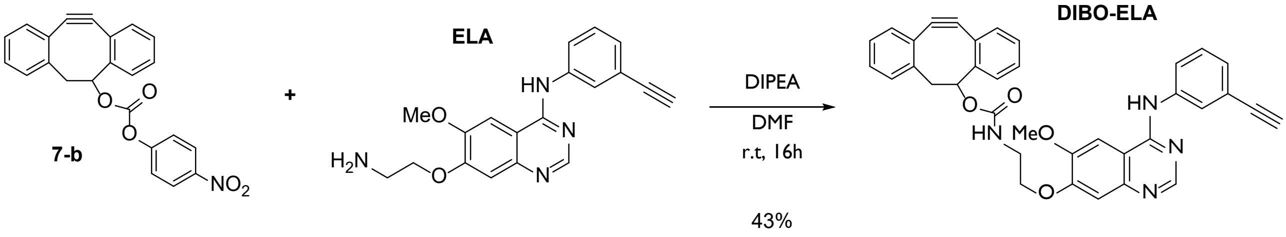

ELA (25 mg, 0.05 mmol, 1.0 eq) and compound **7-b** (20 mg, 0.05 mmol, 1.0 eq) were dissolved in dry DMF (2 mL). DIPEA (44 µL, 0.25 mmol, 5 eq) was added and the reaction was stirred at r.t for 16h under argon. The solvent was removed under reduced pressure and the crude was purified by FCC (DCM / MeOH, 30:1) to afford the **DIBO-ELA** as a white solid (13 mg, 0.022 mmol, 43%).

IR (neat, cm^{-1}): 2954, 2925, 2854, 1718, 1633, 1569, 1511, 1450, 1279, 1158, 1075, 760.

HRMS (nanochip-ESI/LTQ-Orbitrap) m/z: [M + H]+ Calcd for $C_{36}H_{29}N_4O_4^+$ 581.2183; Found 581.2189.

^{1}**H NMR** (400 Hz, $CDCl_3$): δ 8.50 (s, 1H, $H_{Ar, ELA}$), 7.89 (d, J = 6.4 Hz, 1H, $H_{Ar, ELA}$), 7.80 (d, J = 7.4 Hz, 1H, NH_{ELA}), 7.48 (d, J = 7.3 Hz, 1H, $H_{Ar, DIBO}$), 7.41 – 7.19 (m, 11H, 7x $H_{Ar, DIBO}$, 4 x $H_{Ar, ELA}$), 5.65 (t, J = 6.0 Hz, 1H, $H_{Ar, ELA}$), 5.51 (s, 1H, $NH_{carbamate}$), 4.22 – 4.09 (m, 2H, CH_2), 4.02 (s, 3H, CH_3), 3.93 (s, 1H, CH_{DIBO}), 3.69 (d, J = 5.7 Hz, 2H, CH_2), 3.17 (d, J = 14.9 Hz, 1H, CH_{DIBO}), 2.98 – 2.82 (m, 1H, CH_{DIBO}).

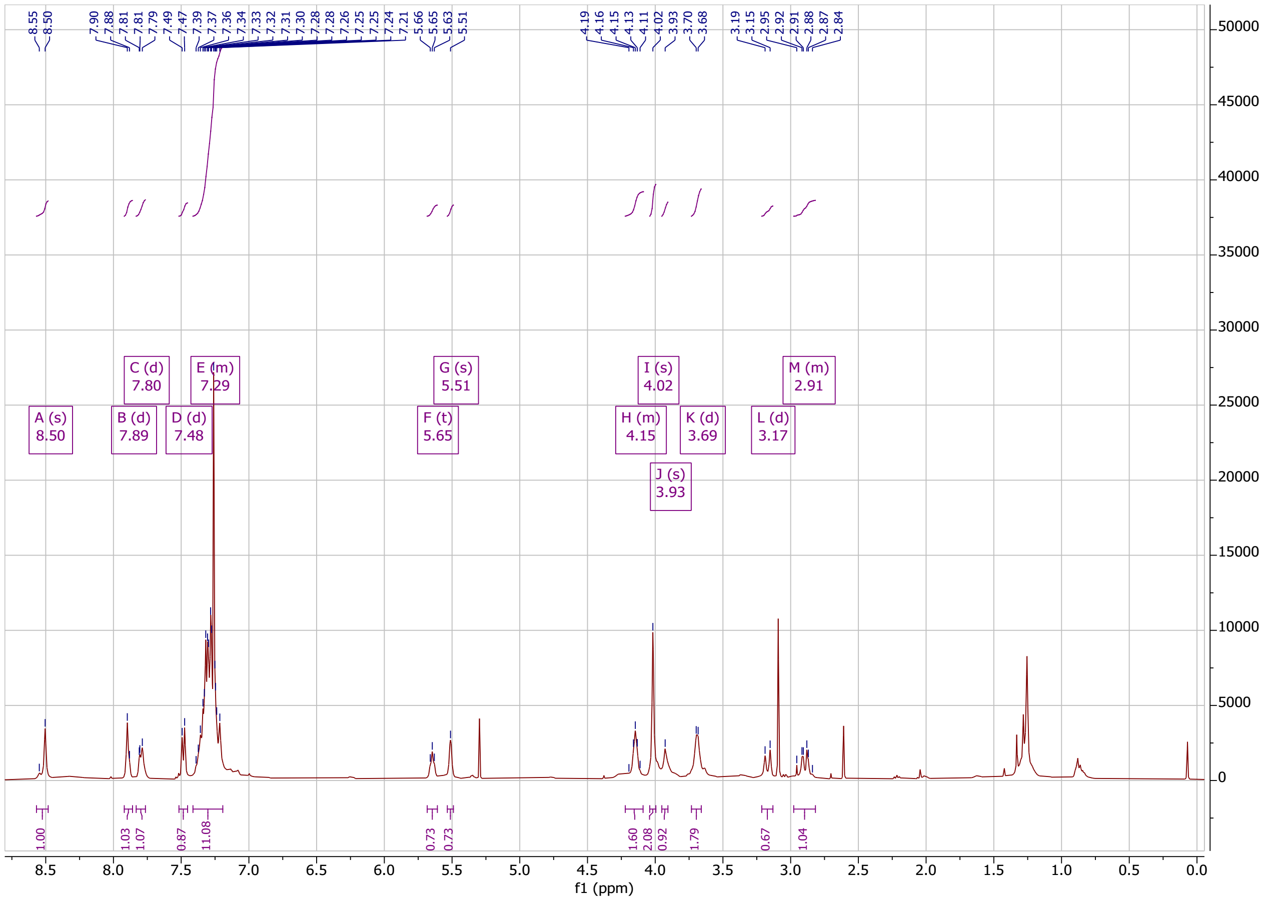

^{13}C NMR (101 Hz, $CDCl_3$): 182.2 (C_{Ar}), 177.3 (C_{Ar}), 155.7 ($C_{carbamate}$), 154.6 (C_{Ar}), 151.0 (C_{Ar}), 150.1 (C_{Ar}), 130.1 (C_{Ar}), 129.2 (C_{Ar}), 128.6 (CH_{Ar}), 128.2 (CH_{Ar}), 128.1 (CH_{Ar}), 127.3 (CH_{Ar}), 127.2 (CH_{Ar}), 126.4 (CH_{Ar}), 126.1 (CH_{Ar}), 125.7 (CH_{Ar}), 123.9 (C_{Ar}), 123.8 (C_{Ar}), 123.0 (C_{Ar}), 121.4 (C_{Ar}), 110.1 (C_{Ar}), 83.3 (C_{alkyne}), 77.9 (CH_{alkyne}), 56.7 (CH_3), 46.2 (CH_2), 41.4 (CH_2), 30.1 (CH_2), 14.4 (CH_2).

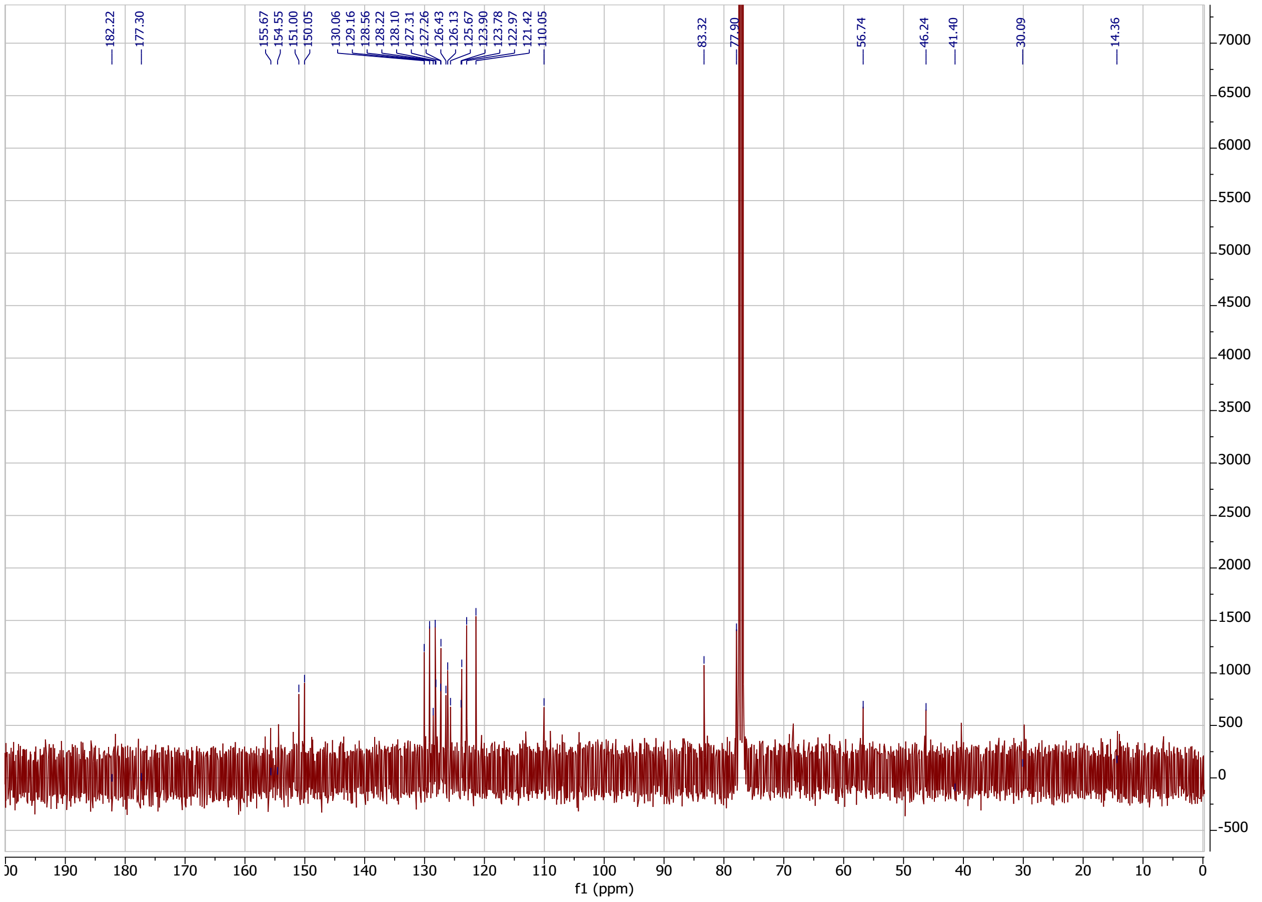

XRD and TEM characterization of LNO HNPs

LNO HNPs were produced under solvothermal conditions (T = 235°C, 24 h) at low hydrolysis rate of a mixture of lithium and niobium alkoxides dissolved in ethanol and butane-1,4-diol. This protocol was adapted from Urbain, M.; Riporto, F.; Beauquis, S.; Monnier, V.; Marty, J. –C.; Galez, C.; Durand, C.; Chevolot, Y.; Le Dantec, R.; Mugnier, Y. On the Reaction Pathways and Growth Mechanisms of $LiNbO_3$ Nanocrystals from the Non-Aqueous Solvothermal Alkoxide Route. *Nanomaterials* **2021**, *11*:154.

Figure S1. XRD diffraction pattern of LNO NPs. The mean nanocrystal size derived at 75 nm from Le Bail refinement is consistent with the representative TEM image given in inset.

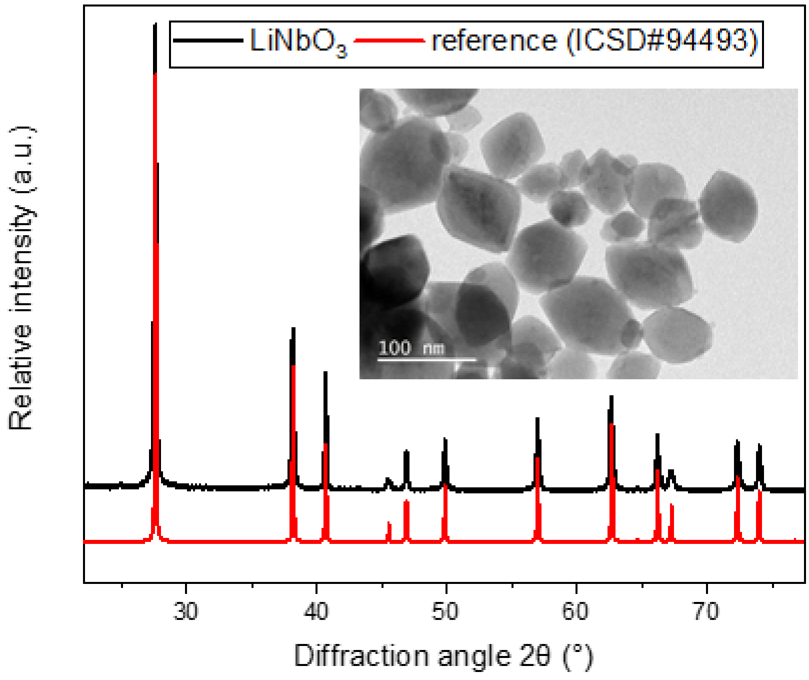

Preparation of LNO-N_3 NPs

Adapted from protocols described in:

Vuilleumier, J.; Gaulier, G.; De Matos, R.; Ortiz, D.; Menin, L.; Campargue, G.; Mas, C.; Constant, S.; Le Dantec, R.; Mugnier, Y.; Bonacina, L.; Gerber-Lemaire, S. Two-Photon-Triggered Photorelease of Caged Compounds from Multifunctional Harmonic Nanoparticles. *ACS Appl. Mater. Interfaces*. **2019**, *11*, 27443-27452.

Scheme S5. Surface coating of **LNO** NPs

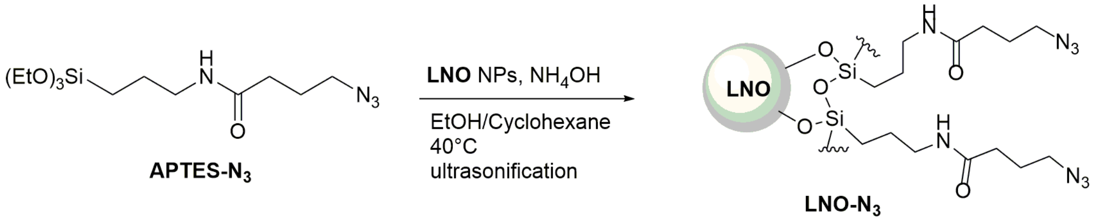

To a suspension of **LNO** NPs (2 mg) in EtOH (1 mL) was added cyclohexane (1 mL). The mixture was ultra-sonicated for 30 min. A solution of 4-azido-*N*-(3-(triethoxysilyl)propyl)butanamide (**APTES-N_3**, prepared according to the protocol reported in Vuilleumier et al.) (1.7 mg, 5 µmol, 1.0 eq.) in EtOH/cyclohexane (1:1,

100 μL) was added and the suspension was ultrasonicated for 30 min. Aqueous NH_3 25 % (100 μL) was added and the suspension was ultrasonicated for 16 h (40-60°C). The suspension was centrifuged (10 min, 4 700 rpm). The supernatant was discarded and the NPs were suspended in EtOH (1 mL). The suspension was shaken until emulsification and centrifuged (10 min, 4 700 rpm). The procedure was repeated 4 times. **LNO-N_3** NPs were stored in EtOH at a concentration of 2 mg/mL. The nanoparticles were characterized by dynamic light scattering and scanning transmission electron microscopy with energy-dispersive X-ray elemental mapping.

Figure S2. **A**: Representative STEM images of an ensemble of **LNO-N_3** NPs. High-angle annular dark-field image; Nb EDX map; O EDX map; Si EDX map; N EDX map; scale bar: 300 nm. **B**: EDX elemental mapping spectrum.

A

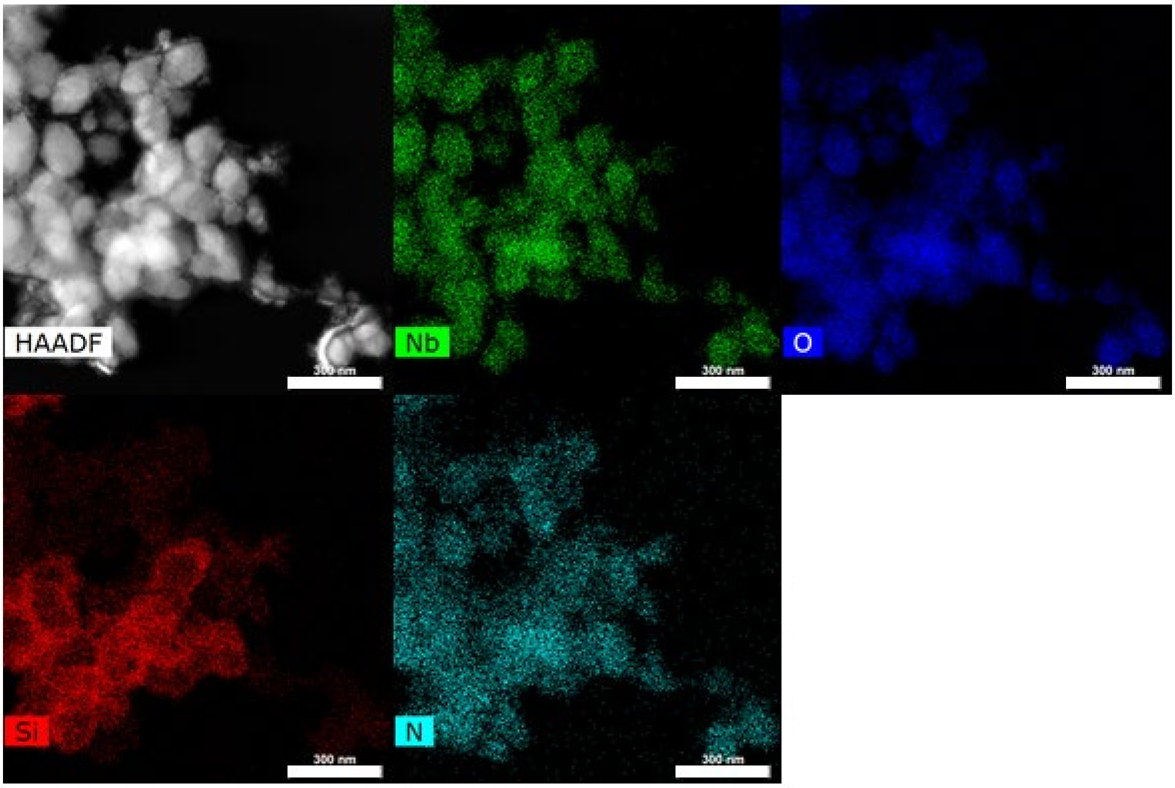

B

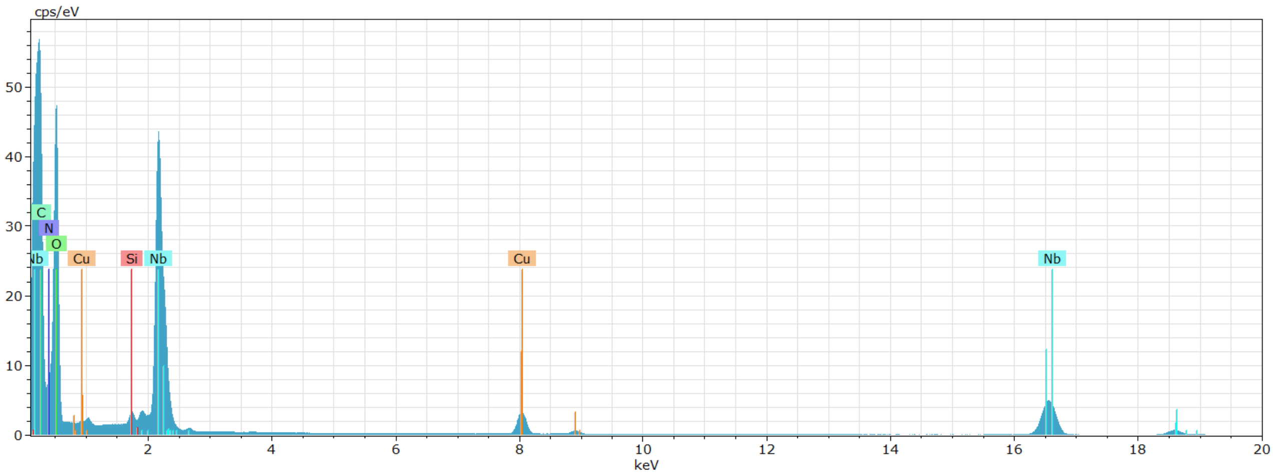

Determination of ELA loading at the surface of LNO-CM-ELA NPs

The grafting of **ELA** on **LNO-N_3** NPs was estimated indirectly by measuring the amount of unreacted **DIBO-CM-ELA** in the supernatant after the functionalization procedure. The recovered supernatant was evaporated *in vacuo*, dissolved in EtOH (1mL) and diluted 10 times in EtOH. The UV-Visible absorption of the as prepared supernatant was then measured using a BioTek® Synergy 2 multi-mode reader between 250 and 450 nm with a 1 nm increment in a 96-well plate (Corning® UV-Transparent microplate) and compared to a set of **DIBO-CM-ELA** standards in EtOH at concentrations ranging from 1 to 250 µM (volume of 150 µL per well). The concentration of **ELA** was linked to the integral of the absorbance spectrum between 250 and 450 nm. The total amount of **ELA** grafted was estimated to 27 nmol/mg of LNO NPs.

Figure S3. Quantification of unreacted **CM-ELA** in the supernatant from the conjugation reaction of **CM-ELA** to **LNO-N_3** NPs. **A:** UV-vis absorbance spectra between 250 and 450 nm at different **CM-ELA** concentrations; **B:** Calibration curve for the indirect determination of conjugated **CM-ELA**.

A

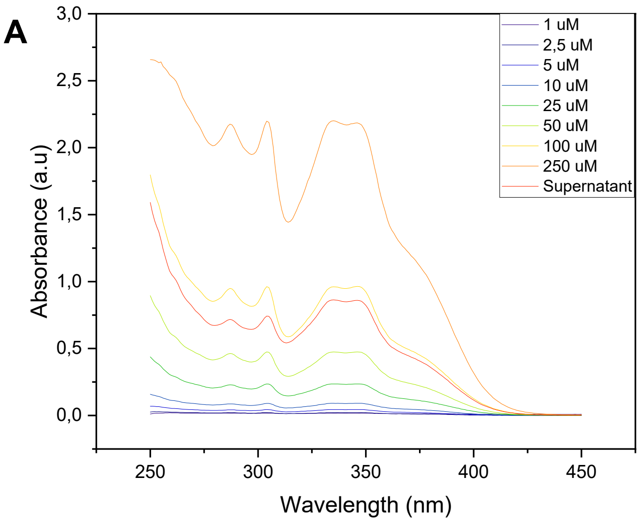

B

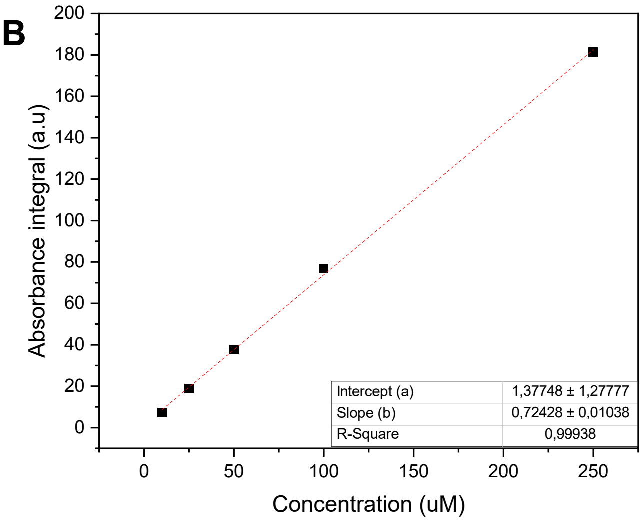

EGFR expression in DU145 cells

Determination of the mRNA levels of EGFR was carried out by real-time quantitative (q)-PCR analysis using a QuantStudio™ 6 Flex Real-Time PCR system (Thermofisher). Total RNA from DU145 and HEK-293 cells was extracted using the RNeasy® Mini Kit (Qiagen) according to the manufacturer's protocol. cDNA synthesis was performed using the PrimeScript™ RT Master Mix (Takara) from 2 µg of total RNA. q-PCR mixtures were prepared in 1× Taqman universal Mastermix (Applied Biosystems) using 50 ng of the reverse-transcribed RNA and EGFR TaqMan gene expression assays (Thermofisher). Glyceraldehyde-3-phosphate dehydrogenase (GAPDH) mRNA was used as an invariant internal control. EGFR expression was normalized to *Gapdh* expression and quantified using the ΔΔCT method.

Figure S4. EGFR expression in HEK-293 and DU145 cells. The relative expression of EGFR mRNA in HEK-293 and DU145 cells was assessed by q-PCR. Values were normalized to the expression of GAPDH. Results are expressed as the mean ± SD of 3 independent experiments.

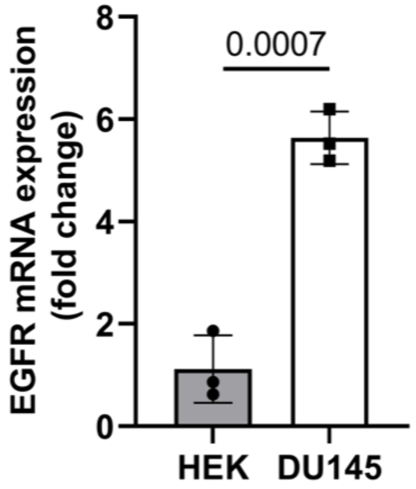

Characterization of UV light- and NIR excitation-triggered release of ELA from LNO-CM-ELA NPs

The amount of **ELA** released upon UV-A irradiation or NIR excitation was plotted as a function of irradiation time and fitted with a mono-exponential function according to equation (1). Equation (1) was differentiated with respect of time (t) to get the initial release rate constant at a given time point (equation (2)).

$$[ELA] = A(1 - \exp(-k * t)) \quad (1)$$

$$\frac{d}{d(t)}[ELA]_t = A * k(\exp(-k * t)) \quad (2)$$

A: pre-exponential factor
k: rate constant
t: time

Release of **ELA** as a result of UV-A irradiation

UV-induced photolysis experiments were performed with a Sylvania UV-light tube (366 nm, 8W), and **ELA** release was evaluated by UHPLC-ESI-HRMS.

The UV flux was measured using a photodiode power-meter (S120C – Thorlabs). When set under the UV lamp at the sample position, the measured value was 281.7 µW, which corresponds to an intensity of 0.38 mW/ cm^2 by taking into account the dimensions of the active sensor surface (0.73 cm^2). Considering that the dimensions of each square multi-well compartment used for the irradiation is $(12\ mm)^2$, the corresponding flux per well can be estimated to 265 µW.

The **ELA** concentration values reported are measured in aliquots taken and diluted to a 1:5 ratio in PBS. A time-dependent increase of released **ELA** was measured upon irradiation of **LNO-CM-ELA** NPs (0.15 mg/mL in PBS). The initial release rate (k_0), calculated at 5 min and normalized from fitted parameters, was estimated at 381 $nM \cdot min^{-1}$.

Table S1. Ratio and percentage of **ELA** released upon irradiation of **LNO-CM-ELA** NPs (loading 0.15 mg/mL) at 366 nm (UV lamp) in PBS 1x. Aliquots of the suspension were withdrawn at the indicated time points, diluted 1:5 in PBS and centrifuged (10 min, 13'000 rpm). Quantification of **ELA** in the supernatant was performed by UHPLC-MS on triplicates.

Irradiation time (min)	**ELA** concentration (nM)	**ELA** Release (%)
0	30.8 ± 5.7	3.8 ± 0.7
1	167.5 ± 19.0	20.7 ± 2.3
2	241.3 ± 8.9	29.8 ± 1.1
5	341.1 ± 27.7	42.1 ± 3.4
10	454.1 ± 13.4	56.1 ± 1.7
15	453.3 ± 4.9	56.0 ± 0.6
20	532.8 ± 13.0	65.8 ± 1.6

Figure S5. Progress of the release of the caged **ELA** upon irradiation at 366 nm / 8W.

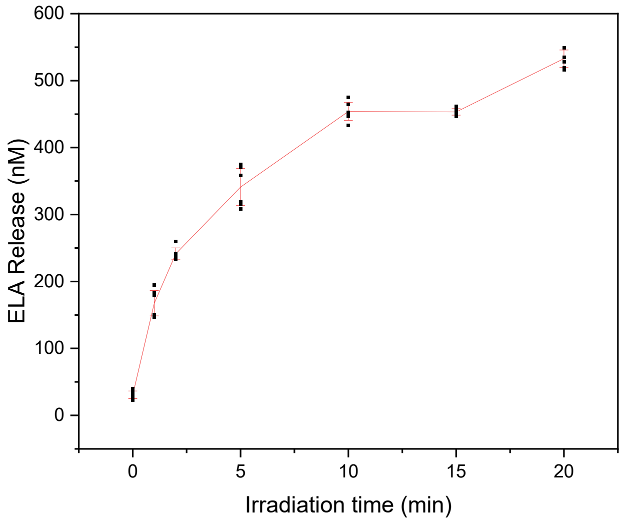

<u>Release of **ELA** as a result of NIR excitation</u>

NIR-induced photolysis experiments were performed upon irradiation by the amplified Ti:Sapphire laser system (Astrella, Coherent) at 790 nm, and **ELA** release was evaluated by UHPLC-ESI-HRMS. The **ELA** concentration values reported are measured in aliquots taken and diluted to a 1:5 ratio in PBS. A time-dependent increase of released **ELA** was measured upon irradiation of **LNO-CM-ELA** NPs (0.15 mg/mL in PBS). The initial release rate (k_0), calculated at 5 min and normalized from fitted parameters, was estimated at 271 $nM \cdot min^{-1}$.

Table S2. Ratio and percentage of **ELA** released upon irradiation of **LNO-CM-ELA** (loading 0.15 mg/mL) at 790 nm (Ti:sapphire pulsed laser) in PBS 1x. Aliquots of the suspension were withdrawn at the indicated time points, diluted 1:5 in PBS and centrifuged (10 min, 13’000 rpm). Quantification of **ELA** in the supernatant was performed by UHPLC-MS on triplicates.

Irradiation time (min)	**ELA** concentration (nM)	**ELA** Release (%)
0	14.0 ± 1.4	1.7 ± 0.2
1	88.4 ± 7.5	10.9 ± 0.9
2	162.3 ± 9.7	20.0 ± 1.2
5	272.9 ± 26.4	33.7 ± 3.3
10	318.1 ± 6.3	39.3 ± 0.8
15	333.9 ± 15.4	41.2 ± 1.9
20	389.1 ± 30.6	48.0 ± 3.8

Figure S6. Progress of the release of caged **ELA** upon irradiation with Ti:sapphire pulsed laser system at 790 nm.

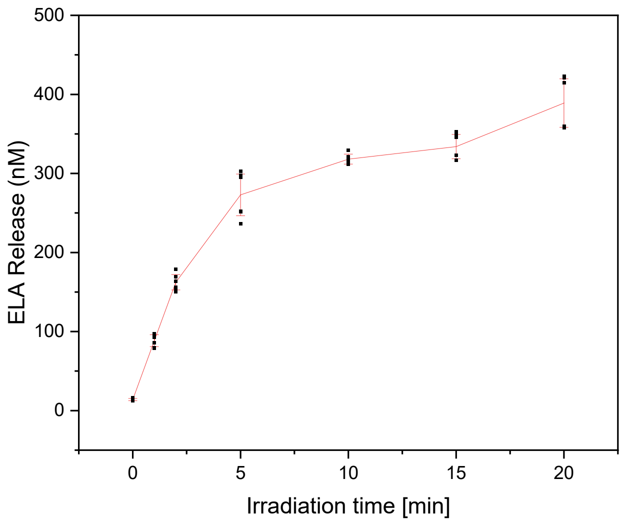

Figure S7. Comparison of the release profiles upon irradiation of **LNO-CM-ELA** at concentrations of 0.075 and 0.15 mg/mL in PBS 1x. **A**: Release upon irradiation at 366 nm. **B**: Release upon irradiation at 790 nm.

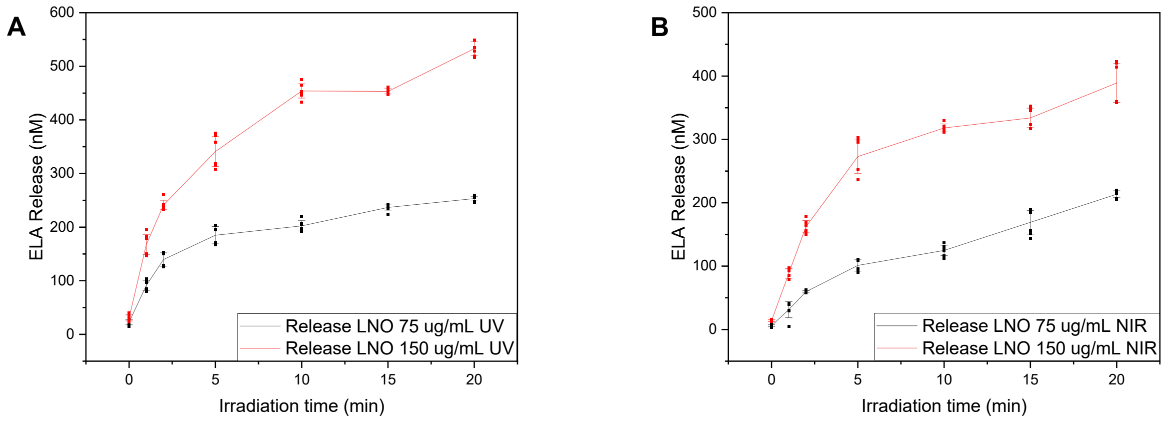

Quantitative analysis by UHPLC-ESI-HRMS

MS spectra were acquired using the 6530 Accurate Mass Q-TOF LCMS mass spectrometer coupled to the 1290 Infinity UHPLC system (Agilent Technologies, USA). Analysis was performed on an ACQUITY UPLC® BEH C18 1.7µm column, 2.1 mm x 50 mm (Waters) heated at 30°C. The mobile phase was maintained at a flow rate of 0.4 mL/min and contained 0.1% (v/v) formic acid water solution (A), and 0.1% (v/v) formic acid acetonitrile solution (B). Over a 4 min total run, the gradient was: 0-0.5 min, 1-5% B; 0.5-2 min, 5-95% B; 2-2.1 min, 95-1% B; 2.1-4 min, 1% B to re-equilibrate the system in initial conditions. The sample manager system temperature was fixed at 15°C and the injection volume was 5 µL. The ESI source was set in positive ionization mode ionization using the Dual AJS Jet stream ESI Assembly. The QTOF instrument was operated in the 4 GHz High Resolution mode in profile mode. The Instrument was calibrated in positive full scan mode using ESI-L+ solution (Agilent Technologies, USA). The TOF mass spectra were acquired over the range of m/z 100-600 at an acquisition rate of 3 spectra/s. ESI AJS settings were as follows RF drying gas flow, 8 L/min; drying gas temperature, 300 °C; nebulizer pressure, 35 psi; capillary voltage, 3500 V; nozzle voltage, 1000 V; fragmentor voltage, 175 V; skimmer voltage, 65 V; octopole 1 RF voltage, 750 V; Sheath gas temperature, 350 °C; Sheath gas low; 11 L/min . Data were processed using the MassHunter Workstation (Agilent Technologies, USA). Extracted ions chromatograms (XIC) were calculated with a window of ±0.5 min using a mass-extraction-window (MEW) of ±50 ppm. The average peak area of three replicate injections at each concentration was used for each data point.

A stock solution of **ELA** was prepared at 1 mM in DMSO. Working standards were prepared in PBS 1x at concentrations of 2500 nM, 1000 nM, 500 nM, 250 nM, 100 nM, 50 nM, 25 nM and 10 nM by using serial dilutions of the stock solution. Extracted ions chromatograms (XIC) were based on a retention time (RT) of 2.01 min with a window of ±0.5 min using a mass-extraction-window (MEW) of ± 50 ppm centered on the m/z_{theor} 335.1502. The average peak area of three replicate injections at each concentration was used for each data point. Calibration curve were fitted with a polynomial order 2 equation, with $R^2 > 0.99$.

Figure S8. **A**: Typical XIC (335.1502 MEW ± 50 ppm) of an **ELA** standard at 250 nM eluted after 2.01 min. **B**: Typical MS of **ELA** standard. **C**: Typical calibration curve for **ELA**.

A

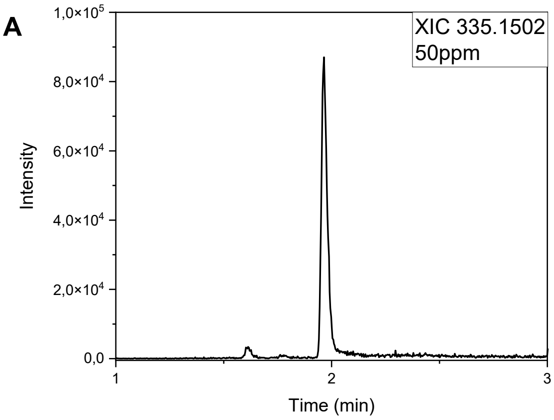

B

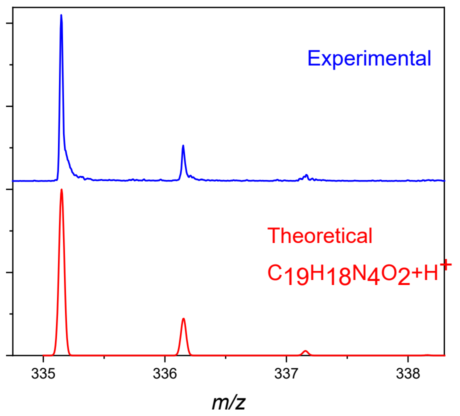
Experimental
Theoretical
C19H18N4O2+H+
335
336
337
338
m/z

C

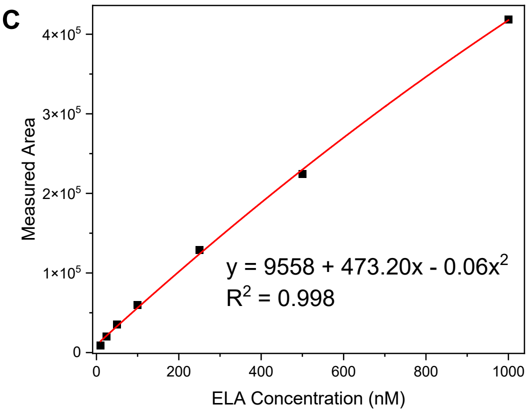
4×10^5
3×10^5
2×10^5
1×10^5
0
Measured Area
0
200
400
600
800
1000
ELA Concentration (nM)
y = 9558 + 473.20x - 0.06x^2
R^2 = 0.998

Irradiation intensity distribution in cell plates

Figure S9. Calculated intensity distribution of the femtosecond beam on the sample during the irradiation protocol. The dashed line corresponds to the well dimensions.

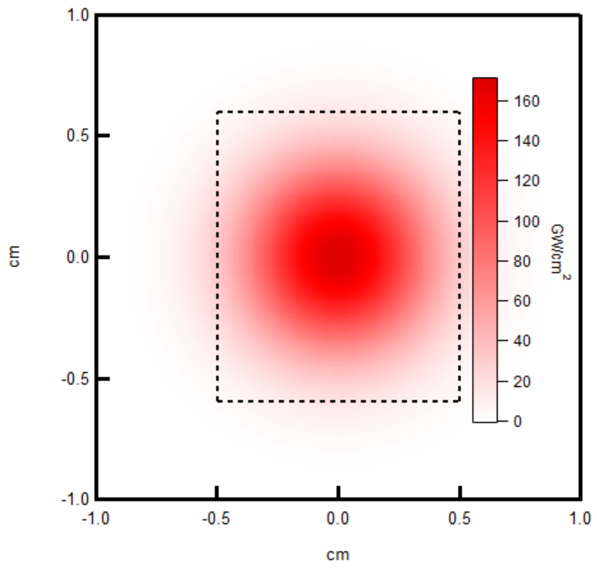

Temperature increase upon NIR laser irradiation

A thermal image of the sample was acquired with an infrared camera (FLIR E8), after a 15 min kHz laser irradiation of laser at 87 GW/ cm2. The maximum temperature observed was < 40° C. For a comparison, the control experiment for the quantification of release upon heating reported in Table 2 was carried out at 50°C.

Figure S10. Thermal image of the irradiated sample.

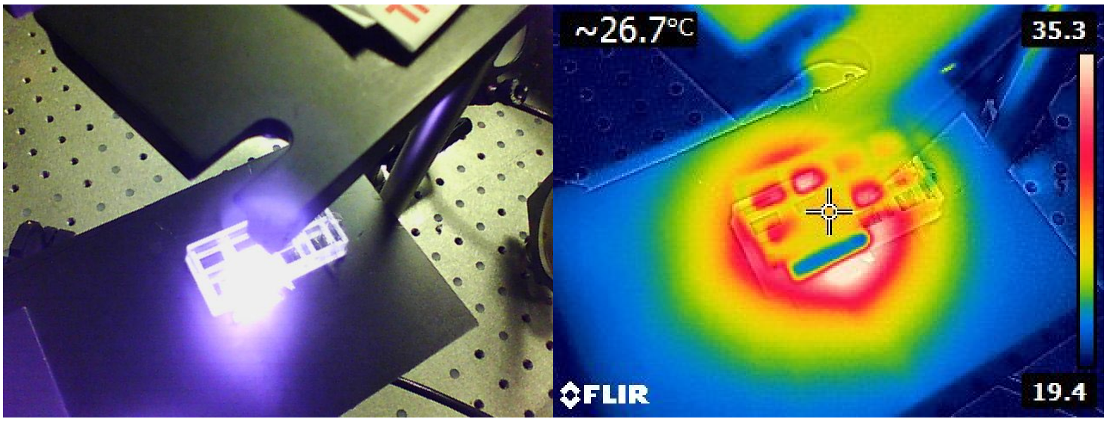

Detection of ELA release upon irradiation on a tunable MHz laser (setup 2)

Suspension of **LNO-CM-ELA** NPs (150 μg) in PBS (1 mL; pH = 7.4, 144 mg/L KH_2PO_4, 9000 mg/L NaCl, 795 mg/L $Na_2HPO_4 \cdot 7H_2O$) were irradiated for 1 h at 800, 1000 and 1100 nm using the irradiation setup 2. Analysis of the supernatants collected from the three different irradiation conditions was performed by UHPLC-MS. **ELA** was only detected in the sample which underwent irradiation at 800 nm (Figure S10).

Figure S11. XIC (335.1504 MEW ± 50 ppm) of the samples irradiated at 800 nm (black curve), 1000 nm (red curve) and 1100 nm (blue curve).

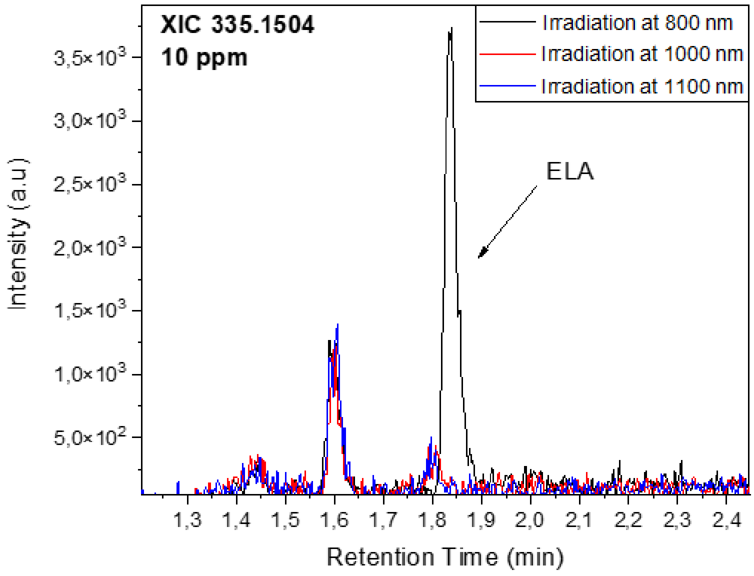

DLS characterization of LNO NPs, LNO-N_3 NPs and LNO-CM-ELA NPs

Figure S12. Size distribution (A) and zeta potential distribution (B) of **LNO**, **LNO-N_3** and **LNO-CM-ELA** NPs measured in PBS buffer (pH 7.4) at 22°C (corresponding to the three first rows of Table 1)

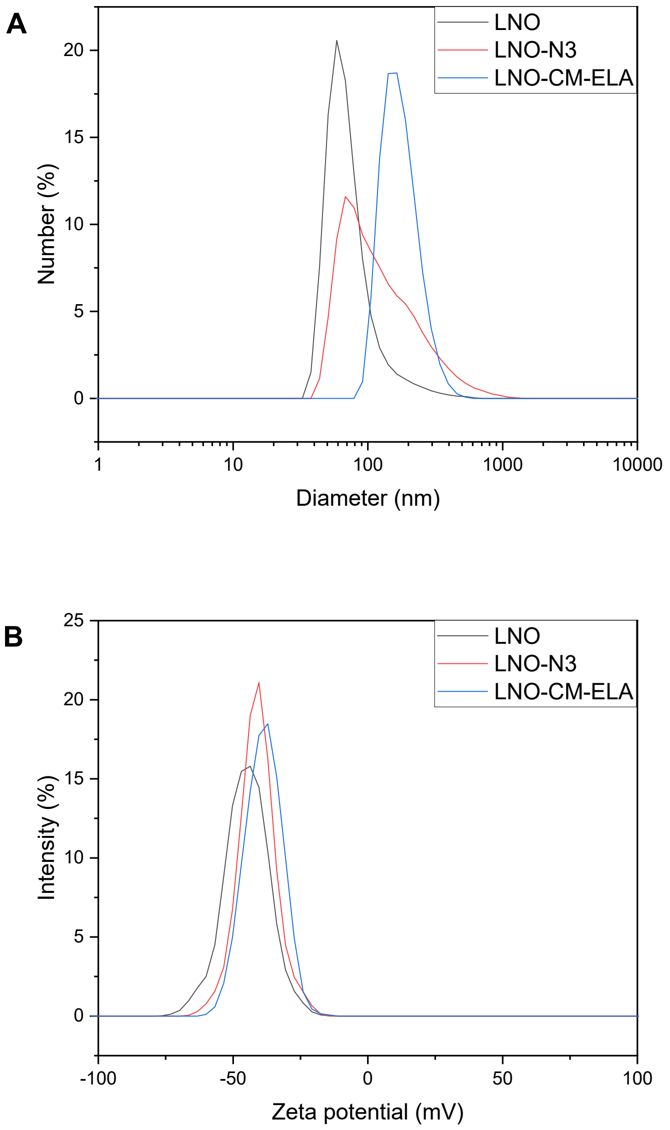

<u>Emission of aggregated LNO-CM-ELA NPs</u>

Whereas isolated objects display pristine SHG spectra upon NIR excitation (see Fig. 2 main text), we report that large particle aggregates might also show a broad luminescence emission, similar to what we previously observed under similar aggregation conditions (Vuilleumier, J.; Gaulier, G.; De Matos, R.; Mugnier, Y.; Campargue, G.; Wolf, J.- P.; Bonacina, L.; Gerber-Lemaire, S. Photocontrolled Release of the Anticancer Drug Chlorambucil with Caged Harmonic Nanoparticles. *Helv. Chim. Acta* **2020**, *103*, e1900251.)

Figure S13. Image and spectral emission profile of the region of aggregated nanoparticles indicated by the dashed outline obtained upon excitation at 800 nm on the Nikon set-up. Scale bar 10 µm.

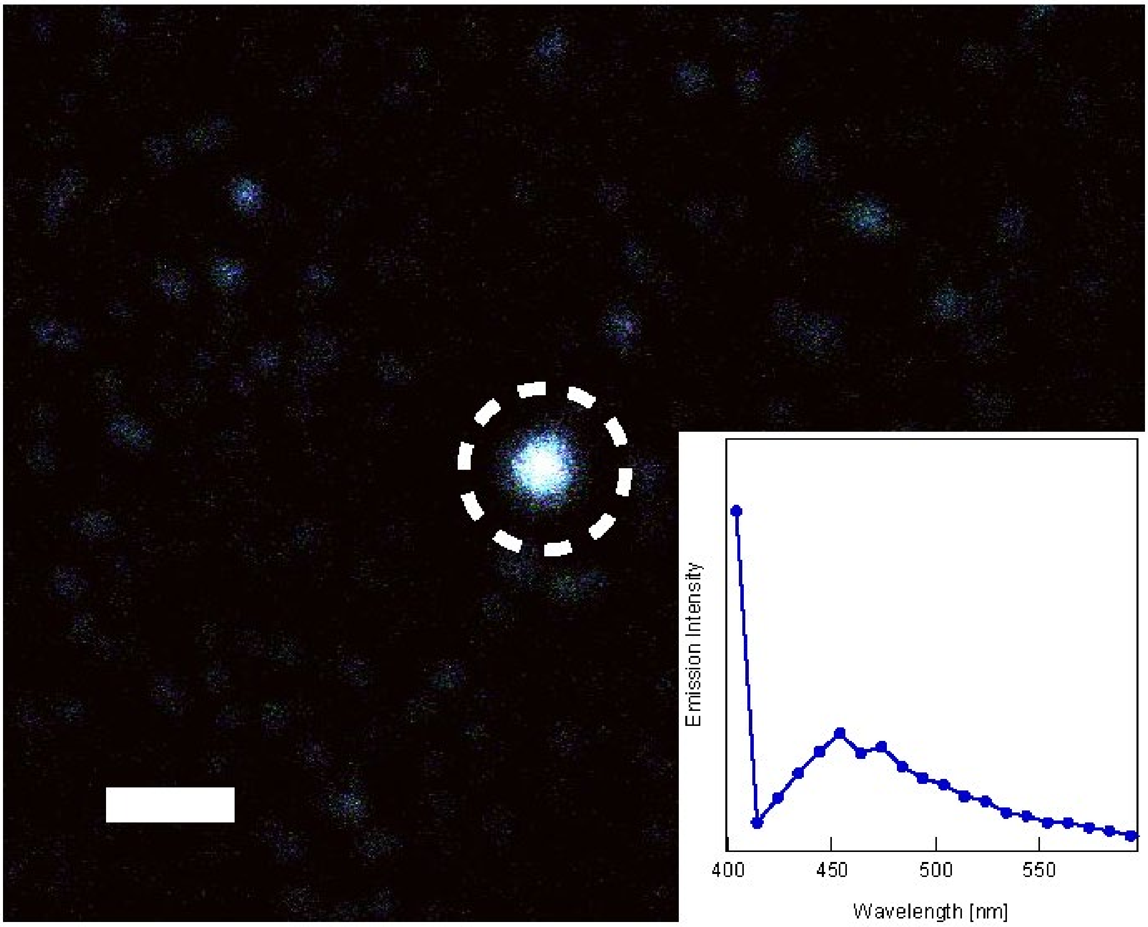

Supplement: Supplementary file 1 — ng1c00044_si_001.pdf [file ng1c00044_si_001.pdf]
